# Social Support in the Perinatal Period: A Systematic Review and Meta‐Analysis of Life Course Antecedents

**DOI:** 10.1111/jan.70677

**Published:** 2026-07-28

**Authors:** Ebony J. Biden, Elizabeth A. Spry, Christopher J. Greenwood, Primrose Letcher, Jacquelyn A. Harverson, Amira Sabrova, Susan Hoffman, Monique N. Gandhi, Lauren M. Francis, Kayla A. Mansour, Liam G. Graeme, Sharon Y. Zhang, Craig A. Olsson, Jacqui A. Macdonald

**Affiliations:** ^1^ Deakin Lifespan Institute, School of Psychology, Faculty of Health Deakin University Burwood Victoria Australia; ^2^ LifeCourse, Murdoch Children's Research Institute Parkville Victoria Australia; ^3^ School of Educational Psychology and Inclusion, Faculty of Education Monash University Clayton Victoria Australia

**Keywords:** interconception, life course, meta‐analysis, midwifery, nursing, perinatal, preconception, social support, systematic review, transition to parenthood

## Abstract

**Background:**

Social support facilitates physical and mental health in the perinatal period for parents and their children. Yet, little is known about what predicts who will have access to support during this uniquely challenging time. From a life course perspective, the foundations of perinatal social support are likely to be evident well before parenthood.

**Aim:**

To systematically review published and grey literature reporting studies that examined associations between parental perinatal social support (pregnancy to 18 months postpartum) and preconception antecedents.

**Methods:**

We searched PsycINFO, Medline Complete, CINAHL Complete, Embase, PsycEXTRA, Open Dissertations, OPENGREY and the first 10 pages of Google and Google Scholar. Effects were meta‐analysed using univariate random effects models.

**Results:**

In 143 included studies published between 1986 and 2023, 46 potential preconception antecedents of parents' perinatal social support were reported. Meta‐analyses revealed lower support was associated with a history of adverse childhood experiences (ACEs), mental health disorder/s, migration, having a subsequent child, and unplanned pregnancy. Evidence of differences by structural and functional aspects of social support was limited. Most studies assessed parents' life histories retrospectively. Few studies included fathers or relational antecedents.

**Conclusions and Implications:**

Evidence supports a life course perspective to enhance social support in the perinatal periods. Parents who experienced disruption to their developmental environment preconception, as early as childhood, may experience inadequate support in early parenthood, potentially perpetuating intergenerational cycles of psychosocial risk. When making referrals, nursing and other healthcare practitioners should consider, in collaboration with patients, whether supports are appropriate and responsive to parents' broader developmental contexts.

**Patient or Public Contribution:**

No patient or public involvement.

## What Does This Paper Contribute to the Wider Global Clinical Community?

- Many parents may lack adequate social support in the perinatal period, a vital resource for healthy family functioning. This review synthesises the existing evidence of the life course antecedents of perinatal social support, so that healthcare practitioners may be better able to work collaboratively with their patients to tailor their care and recommended supports in ways that consider parents' broader developmental contexts.
- Parents' experiences of perinatal social support are associated with several factors across the preconception and interconception developmental ecologies. The greatest risk factor identified for lower perinatal social support was a history of adverse childhood experiences.
- Evidence highlights that those with disrupted developmental environments prior to becoming a parent may experience lower social support in early parenthood and may benefit from additional supports. Nursing and other healthcare practitioners could integrate information about parents' preconception histories, beyond the immediate pre‐pregnancy period, and interconception histories to inform referrals to appropriate supports.

## 1 Introduction

### 1.1 The Importance of Perinatal Social Support

In the perinatal period, defined here from conception through to 18 months postpartum, parents typically experience increased stress and demands, new challenges and routines, and changing roles, goals and identities (Booth et al. 2018; Borschmann et al. 2019; Bost et al. 2002; Livingston et al. 2021; O'Dea et al. 2023; Westrupp et al. 2022). Supporting parents across this uniquely demanding period of the life course has long been a priority area for both public health policy and health service delivery internationally (Buultjens et al. 2013; Zisman‐Ilani et al. 2023; World Health Organization 2025). Reports from the World Health Organization (2025) and the OECD (2025) affirm the central role of social connection in the health and wellbeing of populations around the world, but do not draw attention to the perinatal period. Adequate perinatal social support plays a central role in buffering the negative effects of stress and promotes positive adjustment to pregnancy and infant caregiving, both in new and experienced parents (Battulga et al. 2021; Bedaso et al. 2021; Brown et al. 2025; Cobb 1976; Cohen and Wills 1985; Thoits 1982).

Social support is a multidimensional and complex construct variously defined and measured (Williams et al. 2004). We employ a definition commonly adopted within the context of early parenthood that characterises social support as an individual's belief that they are cared for, loved, esteemed, and belong to a network of mutual obligation (Cobb 1976; Williams et al. 2004). This conceptualisation allows an investigation of parents' global experiences of social support, and also its distinct structural and functional aspects pertinent to the perinatal period, such as sources and functions of support. Several key *sources of support* include partners, family, friends, and health professionals (Antonucci and Akiyama 1987; Battulga et al. 2021; Bost et al. 2002; Gariépy et al. 2016; Leahy‐Warren et al. 2023; Vaux 1990; Wrzus et al. 2013; Yim et al. 2015). Important *support functions* include emotional support (the expression of care, concern, understanding, inclusion), instrumental support (provision of tangible aid), informational support (communication of knowledge, facts, advice), and appraisal support (feedback about behaviours or personal qualities relevant for self‐evaluation; House 1981; Shumaker and Brownell 1984).

Empirical evidence attests to wide‐ranging benefits of social support for parents during the perinatal period, from reduced depression, anxiety and stress to increased role adaptation and confidence in infant care (Edward et al. 2019; Hetherington et al. 2018; Leahy‐Warren 2005; Leal et al. 2021; Ni and Siew Lin 2011; Philpott et al. 2019; Racine et al. 2019; Radey 2018; Sufredini et al. 2022; Vallin et al. 2019; Zamani et al. 2019), benefitting family dynamics and parenting practices (Brough and Sheppard 2022; Chang 2017; Daniele 2021; Fang et al. 2021; Fisher et al. 2021; Leahy‐Warren et al. 2023; Kirova and Snell 2019; Radey 2018; Vallin et al. 2019), and ultimately showing long‐lasting and intergenerational effects (Biden et al. 2021; Poggi and Kalb 2020; Schuijers et al. 2024; Thomas et al. 2017; Tsvetkova and Macy 2014). Of note, perceived social support is more reliably associated with health and well‐being compared to actual received support (Thoits 2011; Uchino 2009). Social support not only prevents and buffers the negative effects of stress, it provides parents with existential affirmation, adding meaning to life through shared positive experiences and emotions within close relationships (Feeney and Collins 2015; Lakey and Cohen 2000; Ramsey and Gentzler 2015; Thoits 1982; Vaux 1990). Parents with access to social support can draw on this store of resources to cope with major and minor life stressors, meet the demands and their goals of parenthood, and thrive during this life phase (Feeney and Collins 2015; Vacchiano and Spini 2021; Vaux 1990).

### 1.2 Intervening on Perinatal Social Support

There is longstanding recognition of the need to intervene on and improve social support for parents (Buultjens et al. 2013; Zisman‐Ilani et al. 2023). Many parents report having low or inadequate levels of perceived and received social support (Australian Institute of Health and Welfare 2020; Leahy‐Warren et al. 2023). Perinatal social support interventions generally target the pregnancy or postpartum periods, addressing proximal factors and vulnerable groups to varying degrees of success (Balaam et al. 2022; Geens et al. 2014; Kirubarajan et al. 2022; Leal et al. 2021; McLeish and Redshaw 2017; Misra et al. 2003; Reyes et al. 2021).

There is evidence that such perinatal social support interventions can be successful in promoting parents' short‐term mental health, wellbeing and confidence in parenting (Balaam et al. 2022; Bennett et al. 2017; McLeish and Redshaw 2017; Reyes et al. 2021; Sufredini et al. 2022). However, it is unclear how effective these interventions are for promoting social support itself, and for how long, as it is often not measured as an outcome (see Balaam et al. 2022; Bennett et al. 2017; McLeish and Redshaw 2017; Reyes et al. 2021; Sufredini et al. 2022). There is some evidence to suggest that social support interventions may be limited in their capacity to mobilise and promote social support, most regrettably failing those who may need it most (e.g., economically disadvantaged parents, young parents, parents of children with complex needs; Reyes et al. 2021; Sartore et al. 2021; Sufredini et al. 2022).

The foundations of parents' social support systems take time to develop and fortify and are under formation in the developmental stages that precede parenthood (Holden et al. 2015; Langheit and Poulin 2022; Wrzus and Neyer 2016). Therefore, having access to secure, high‐quality social support in the perinatal period is likely to have origins in the years prior to conception and may have distinct predictors across development. Investigating the developmental antecedents of perinatal social support may help nursing and other healthcare practitioners identify early which parents may be at risk of experiencing lower social support. Identified antecedents could also be used to inform the design of support interventions and guide care providers in understanding parents' broader risk contexts during the transition to parenthood, enabling more tailored referrals.

### 1.3 A Life Course Perspective

Life course theory and research suggests that women's perinatal health, and increasingly that men's perinatal health, have their origins in the preconception period (Kotelchuck and Lu 2017; Misra et al. 2003; Patton et al. 2015, 2018; Wilson et al. 2026). Preconception is defined as occurring prior to the conception of offspring, or in between pregnancies (also known as the interception period; Misra et al. 2003). This can be just prior to conception (which is the common perspective) or extend well prior to parenthood, across the early life course of the parent (Stephenson et al. 2018). We take the broader perspective on the definition of preconception health and development, across which the accumulation of advantage or disadvantage compounds through a range of biopsychosocial processes including HPA axis dysregulation, heightened/dampened immune responses, neurocognitive capabilities, and their intersection with access to resources and learnt behaviours (Bernardi et al. 2019; Kane and Margerison‐Zilko 2017; Misra et al. 2003). These come to determine the perinatal ecology of the family microsystem in ways that shape parent and offspring perinatal and later life outcomes (Bernardi et al. 2019; Kane and Margerison‐Zilko 2017; Misra et al. 2003; Patton et al. 2018).

A life course approach provides the perspective needed to fully examine the developmental history of factors that might influence or modify social support in the perinatal period. However, life course antecedents of perinatal social support occurring preconception have not yet been systematically synthesised in the literature. We draw on Life Course Theory (e.g., Elder et al. 2003) and the Bioecological Model (e.g., Bronfenbrenner 1979, 2005) to guide our examination of the antecedents of perinatal social support. We focus on the dimensions of developmental timing and ecological level to organise our investigation.

#### 1.3.1 Developmental Timing

The social networks and skills that inform levels of social support build progressively across childhood, adolescence and young adulthood, with normative experiences such as attending school and entering the job market expediting this process and representing sensitive periods in social network development (Blakemore and Mills 2014; Langheit and Poulin 2022; Mitic et al. 2021; Wrzus et al. 2013). Based on Life Course Theory (e.g., Elder et al. 2003) and the Bioecological Model (Bronfenbrenner 2005), we posit that the developmental timing and accrual of certain preconception experiences may be associated with the development of social networks and perception of social support in later periods, including early parenthood.

#### 1.3.2 Ecological Level

Informed by the Bioecological Model (Bronfenbrenner 2005), we organised this review of the life course antecedents of perinatal social support around three levels of aetiologic influence within which an individual is a participant in: ‘individual’, ‘relational’ and ‘contextual’. These group together similar factors, both proximal and more distal, that influence individual development discretely and collectively (Bronfenbrenner 1979, 2005; Bronfenbrenner and Morris 2006; Tudge et al. 2009). Specifically, we consider how the development of perinatal social support might be influenced by preconception biographical factors, dispositions and biology (individual antecedents), the reciprocal nature of social interactions with others (relational antecedents), as well as the broader environment and community factors (contextual antecedents; Antonucci et al. 2019; Bernardi et al. 2019; Bronfenbrenner 1979, 2005; Tudge et al. 2009).

## 2 The Review

The benefits of social support in early parenthood have been well documented. In stark contrast, the antecedents of social support, across the life course before parenthood, are not well understood. In this review we set out to identify antecedents of perinatal social support which may have the potential to assist nursing and other healthcare practitioners in the early identification of parents who may benefit from appropriate referrals to supports that are responsive to their developmental contexts. This review is the first to systematically identify antecedents of perinatal social support in the literature and novelly with a life course lens. We theorise that antecedents at each age and stage of parents' preconception life course and across each level of the ecology are important for parents' perinatal social support.

## 3 Aims

We have three broad aims.

Aim 1: To (a) identify evidence for potential life course antecedents of perinatal social support, (b) describe the extent to which antecedents across the parental preconception developmental life course and levels of the ecology predict perinatal social support, and (c) investigate whether associations differ by parent and study characteristics.

Aim 2: To examine whether associations with preconception antecedents differ depending on the measured aspects of perinatal social support, including (a) structural aspects and (b) functional aspects.

Aim 3: To summarise gaps in the literature for future research coverage.

## 4 Methods

This systematic review and meta‐analysis was conducted in accordance with the Preferred Reporting Items for Systematic reviews and Meta‐Analyses 2020 (PRISMA 2020) guidelines (Page et al. 2021) and was pre‐registered with PROSPERO (CRD42020199848).

### 4.1 Search Methods

We searched PsycINFO, Medline Complete, CINAHL Complete and Embase for peer‐reviewed literature including the combined four concepts: social support; the perinatal period; parents; and preconception antecedents. We also searched PsycEXTRA, Open Dissertations, OPENGREY, as well as the first 10 pages (100 articles) of Google and Google Scholar (as per Dowling et al. 2017) for grey literature and canvassed researcher networks for relevant articles. Applying a pragmatic cut‐off reflects established practice in searching grey literature via large relevance‐ranked search engines, balancing yield with screening resources (Haddaway et al. 2015). We conducted the last search on the 5 of December 2023. We limited the search to articles available in English. There were no limits placed on publication date (see Supporting Information 1 for the full search strategy).

### 4.2 Study Selection

We included original research which reported an association between perinatal social support and any preconception antecedent (including retrospective measures), even when that association was not included in the primary aim or analysis of the study (see Supporting Information 1 for the full eligibility criteria). Using the web‐based collaboration software platform Covidence (Covidence Systematic Review Software 2022), articles' titles and abstracts were screened by reviewers E.J.B., K.A.M., L.G.G., L.M.F., A.S., E.A.S., P.L., C.J.G. and J.A.M., with a third of articles blind double screened. Candidate articles' full texts were screened by reviewers E.J.B., A.S., S.H., L.M.F., J.A.M., K.A.M., E.A.S., C.J.G., L.G.G. and P.L., with a quarter of articles blind double screened to manage the volume of results. Disagreements were resolved through a consensus process.

### 4.3 Data Extraction

Data were extracted into a standardised template by E.J.B., J.A.H., M.N.G., S.H., A.S. and S.Y.Z., with all extracted effects and analytic sample sizes cross‐checked. Unadjusted effects were prioritised to preserve disparities in perinatal social support between antecedent levels or groups as they exist in the population (Conroy and Murray 2020; Fox et al. 2022; see Supporting Information 1 for more information on data extraction).

### 4.4 Quality Appraisal

The quality of included studies was assessed by E.J.B. using an adapted version of the National Institutes of Health Quality Assessment Tool for Observational Cohort and Cross‐Sectional Studies (National Heart Lung and Blood Institute: National Institutes of Health 2014). This tool was adapted to 12 items (see Supporting Information 1) covering four domains of relevance to this review: generalisability, selection, measurement and power.

### 4.5 Statistical Analyses

Where effect size data were not reported as correlation coefficients, data were converted to Pearson correlation coefficients (*r*). All effect sizes are presented as correlations. We interpret effect sizes as small (≈0.10), moderate (≈0.20), and large (≈0.30) to acknowledge the importance of potential cumulative effects over time (Funder and Ozer 2019). The Summary Table and Data Frame and the meta‐analytic code can be found here: https://osf.io/AZVUE/.

To quantitatively synthesise the literature, we examined meta‐analytic associations between perinatal social support and each preconception antecedent in all included studies when the number of studies was ≥ 2 (Ryan et al. 2016). Antecedents were categorised according to ecological level and developmental timing (see Supporting Information 1 for more information on how antecedents were grouped for meta‐analyses). We meta‐analysed unadjusted estimates of the associations between antecedents and the outcome of perinatal support including all structural and functional aspects. The meta‐analyses were conducted in R version 4.2.1 with ‘robumeta’ (R Core Team 2022; Fisher et al. 2017), using univariate random effects models based on the assumption of between‐study heterogeneity. Specifically, we account for estimates nested within studies (i.e., when studies reported multiple effects) using robust variance estimation with small sample adjustment (Hedges et al. 2010; Tipton 2015). Each correlation was converted to Fisher's *z*‐scores for analysis, then back‐transformed for interepretability.

We conducted sensitivity analyses to determine whether meta‐analytic effects were robust to a wide range of assumed within cluster correlations (rho values). We used *p*‐values derived from Egger's tests to assess funnel plot asymmetry as an indicator of publication bias using the R package ‘metafor’, by first aggregating non‐independent effects (Egger et al. 1997; Viechtbauer 2010). We used *I* ^2^ to assess the ratio of true to total variance (i.e., what proportion of the variance in observed effects is due to variance in true effects, rather than sampling error) in each meta‐analysis (Borenstein et al. 2017; Higgins et al. 2003).

Meta‐regressions were used to examine the impact of each proposed moderator (parent and study characteristics, and aspects of social support; see Supporting Information 1) on the associations. For categorical moderators, we used *p*‐values derived from Wald tests to assess if the associations in levels of a moderator were different from one another using the R package ‘clubSandwich’ (Pustejovsky 2020) and presented the meta‐analytic associations within stratifications.

## 5 Results

### 5.1 Review Summary

The article selection process is described in the PRISMA flow diagram presented in Supporting Information 1. We identified 13,242 articles from the search of databases after duplicates were removed. Articles' titles and abstracts were screened, leaving 1597 unique candidate articles which were identified as potentially meeting the inclusion criteria. At the full text screening stage, 1455 articles were excluded as they did not meet the full inclusion criteria; the majority of articles (52%) were excluded as they did not report sufficient data to calculate relevant effect sizes. Following the screening of the full texts, 143 articles were identified as meeting the full inclusion criteria (see Supporting Information 1 for the reference list of included studies).

Studies meeting the inclusion criteria were conducted in 33 different countries. Approximately one third of studies were conducted in the United States of America (35%). Most studies were conducted in high income countries (78%) and upper‐middle income countries (15%; The World Bank 2022). Studies rarely examined parents' perinatal social support experiences in lower‐middle income countries (6%, e.g., India, Iran, Nigeria, Pakistan and Zimbabwe) and low income countries (1%, e.g., Ethiopia and Uganda; The World Bank 2022). Study data were collected between 1980 and 2022, and studies were published between 1986 and 2023. Approximately one fifth of included articles were published since 2021 (19%). The majority of included studies were journal articles (96%), with remaining studies being conference abstracts (2%) and theses (2%).

A large majority of studies examined the perinatal social support experiences of parents between the sample mean ages of 20 and 34 years (89%; 6% age not reported). Studies meeting the inclusion criteria rarely examined younger (mean age ≤ 19 years = 3%) or older (mean age ≥ 35 years = 2%) parents' perinatal experiences (OECD 2022; Saloojee and Coovadia 2015). Fewer than one in 10 studies recruited and reported data separately for fathers (8%).

Very little research (2%) was based on multi‐year prospective longitudinal study data with preconception assessment. For example, substance use was assessed prospectively several years before pregnancy in the Australian Longitudinal Study of Women's Health (Powers et al. 2013), and preconception mental health and personality were assessed prospectively during adolescence and young adulthood in the Victorian Intergenerational Health Cohort Study (Spry et al. 2021, 2023). Other longer term prospective cohort studies were also included, however, the preconception antecedents of interest were assessed retrospectively (e.g., being adopted and living in out of home care in childhood/adolescence in the Avon Longitudinal Study of Parents and Children; Teyhan et al. 2018). For further descriptive information regarding individual studies, including study characteristics and results, see the Summary Table and Data Frame.

### 5.2 Quality Appraisal

The mean study quality score was 7.45 (SD = 1.40, range = 3–11) out of a possible range of 0–12, where higher scores indicate better study quality. Regarding *generalisability*, most studies (95%) clearly specified and defined the study population. There was some evidence of *selection* bias (total *M* = 2.62, SD = 0.90, possible range of 0–4) as many studies (~60%) did not report the participation rate of eligible people invited to participate or reported a participation rate of less than 50% of people approached. There was some evidence of *measurement* bias (total *M* = 2.57, SD = 0.82, possible range of 0–5) due to most studies using retrospective measures of antecedents. Studies performed well on indicators of measurement bias for the outcome of social support, with most studies (92%) assessed as using clearly defined, valid and reliable measures implemented consistently across study participants. Regarding *power*, approximately two‐thirds of studies did not provide a justification of sample size or description of power. All included studies clearly stated a research question or objective.

### 5.3 Aim 1: Life Course Antecedents of Perinatal Social Support

#### 5.3.1 Identify Evidence for Potential Life Course Antecedents of Perinatal Social Support

Operationalisation of ecological levels and developmental stages is detailed in Boxes 1 and 2 respectively. Forty‐six potential preconception antecedents of perinatal social support were found in the literature. They are summarised by developmental timing and ecological level in Box 3. Included studies rarely specified the age of participant preconception exposure, instead retrospectively identifying the broad developmental stage during which the antecedent occurred.

BOX 1 **Operationalisation of ecological levels of life course antecedents.**

**Table:** 

Individual level	*Individual level* antecedents describe the parents' biographical characteristics, internal states (biological and psychological), and behaviours (as informed by Bernardi et al. 2019; Bronfenbrenner 1979, 2005).
Relational level	*Relational level* antecedents refer to constructs regarding interpersonal relationships in which the focal parent is directly involved (as informed by Bronfenbrenner 1979, 2005)
Contextual level	*Contextual level* antecedents capture the external socio‐cultural environments with which the focal parent interacts and is situated in (as informed by Bronfenbrenner 1979, 2005).

BOX 2 **Operationalisation of developmental stages of life course antecedents.**

**Table:** 

← Distal antecedents		Proximal antecedents →	Lifetime history
Childhood and adolescence	Adulthood	Pre‐pregnancy	
*Childhood and adolescent* antecedents were combined as studies did not typically differentiate antecedents occurring before 18 years of age. No studies used an expanded definition of adolescence beyond 18 years of age (see Sawyer et al. 2018). This stage represents the most distal antecedents to the perinatal period.	Antecedents during young or emerging adulthood were few, hence they were grouped as *adult* antecedents more broadly, occurring any time after 18 years of age. No studies described antecedents as occurring in alternative stages of adulthood (i.e., established or middle adulthood as per Mehta et al. 2020).	A number of studies specified antecedents as occurring in the months to years immediately preceding conception and were grouped as *pre‐pregnancy* antecedents (as informed by Stephenson et al. 2018). This stage represents the most proximal antecedents to the perinatal period.	Several studies examined antecedents that occurred any time before next generation conception, which we describe as *lifetime history*. This category also includes particular antecedents which have been measured in varying developmental stages across multiple studies.

BOX 3 **A summary of the potential life course antecedents of perinatal social support reported in the literature, summarised by ecological level and developmental timing.**

**Table:** 

	← Distal antecedents		Proximal antecedents→	Lifetime history
	Childhood and adolescence	Adulthood	Pre‐pregnancy	
Individual level	Perceived stress Anxiety^a^ Perceived socioeconomic position	Personality traits Depression during a previous pregnancy^a^ **History of adverse obstetric outcomes** **Parity** Previous abortion Previous preterm birth **Previous caesarean section** Gestational diabetes in a previous pregnancy^b^ Perception of previous birth experience/s Sex of first‐born child	**Substance use** **Weight/body mass index (BMI)** Anaemia Iron use Folic acid use Mental health service use Hospitalisation **Preconception care engagement** **Pregnancy intention** **Method of conception** Inter‐pregnancy/birth interval Number of in vitro fertilisation (IVF) treatments	**History of mental health disorder/s** **Living with diabetes**
Relational level	Attachment Parenting Family relationship quality Social support	Personal divorce/separation (of focal parent) Intimate partner violence (IPV) Previous custody loss of child/ren	Marital status	
Contextual level	**Adverse childhood (and adolescent) experiences (ACEs)** Adopted Lived in out‐of‐home care Financial and family stressors Socioeconomic position (objective) Parent divorce/separation Older than expected for school grade level Country of birth different from current country of residence^c^ Migration to a new country^c^	Migration to a new country^c^		**Migration history**

*Note:* Bold indicates sufficient data for meta‐analysis. ^a^Included in ‘History of mental health disorder/s’ meta‐analysis, ^b^Included in ‘Living with diabetes’ meta‐analysis, ^c^Included in ‘Migration history’ meta‐analysis.

#### 5.3.2 Describe the Extent to Which Antecedents Across the Parental Preconception Developmental Life Course and Levels of the Ecology Predict Perinatal Social Support

Meta‐analyses were conducted to investigate associations between 12 preconception antecedents and perinatal social support. Insufficient studies were found for the remaining preconception antecedents (i.e., number of studies < 2 for each construct), thus they were not able to be meta‐analysed. Individual estimates between a preconception antecedent and perinatal social support reported in each study can be found in the Summary Table and Data Frame.

Meta‐analytic findings are presented within ecological levels in Table 1 and described in text with reference to developmental timing. The number of studies included in the meta‐analyses (*k*) ranged from 2 to 58 and the number of included effects (*e*) ranged from 3 to 142. The combined number of participants from articles included in each meta‐analysis (*n*) ranged from 389 to 87,971. The ratio of true to total variance in each meta‐analysis ranged from 97% (sampling error can explain 3% of the observed variance) to 35% (sampling error can explain 65% of the observed variance). As indicated by Egger's test (*p*), no publication bias was found for most meta‐analysed antecedents, except for ACEs (*k* = 19, *p* = 0.025) and history of adverse obstetric outcomes (*k* = 3, *p* = 0.002) which showed some evidence of publication bias. Forest plots and funnel plots for each meta‐analysis are presented in Supporting Information 1.

**TABLE 1:** Meta‐analytic correlations between life course antecedents and perinatal social support.

Preconception antecedents	*k*	*e*	*n*	*r*	95% CI	*p*	*I* ^2^	Egger's test (*p*)
*Individual level antecedents*								
History of adverse obstetric outcomes	3	4	19,036	−0.02	−0.17, 0.13	0.621	81.19	0.002
Parity	58	142	49,493	−0.11	−0.16, −0.06	< 0.001	96.20	0.759
Previous caesarean section	2	4	11,177	−0.03	−0.67, 0.61	0.676	95.22	NA*
Pre‐pregnancy substance use	4	5	3,603	−0.04	−0.21, 0.13	0.449	82.26	0.337
Pre‐pregnancy weight/body mass index (BMI)	10	14	23,371	−0.06	−0.17, 0.06	0.282	93.31	0.285
Pregnancy intention (Unplanned)	27	54	32,672	−0.13	−0.18, −0.07	< 0.001	92.18	0.438
Preconception care engagement	2	3	834	0.01	−0.79, 0.80	0.926	65.59	NA*
Method of conception (Assisted reproduction)	5	69	389	−0.14	−0.33, 0.05	0.106	54.65	0.909
History of mental health disorder/s	6	34	3,201	−0.17	−0.31, −0.02	0.030	88.40	0.838
Living with diabetes	2	6	394	−0.03	−0.77, 0.71	0.703	34.67	NA*
*Relational level antecedents*								
None	—	—	—	—	—	—	—	—
*Contextual level antecedents*								
Adverse childhood (and adolescent) experiences (ACEs)	19	46	8,611	−0.22	−0.30, −0.15	< 0.001	83.76	0.025
Migration history	22	61	87,971	−0.11	−0.16, −0.05	< 0.001	97.32	0.644

*Note: k* = number of studies, *e* = number of effects, *n* = combined number of participants from articles. NA* = *k* ≥ 3 required. The following preconception antecedents were commonly coded as binary variables: History of adverse obstetric outcomes = 1, no history of adverse obstetric outcomes = 0; Parity: multiparous = 1, primiparous = 0; Pre‐pregnancy substance use = 1, no pre‐pregnancy substance use = 0; Pregnancy intention: unplanned = 1, planned = 0; Method of conception: assisted reproduction = 1, spontaneous conception = 0; History of mental health disorder/s = 1, no history of mental health disorder/s = 0; Migration history = 1, no history of migration to a new country = 0. Pre‐pregnancy weight/BMI and ACEs were commonly measured as continuous variables, where higher values mean greater weight/BMI and greater exposure to ACEs. Negative correlations indicate that as preconception exposure increases, perinatal social support tends to decrease.

Several individual level preconception antecedents were related to perinatal social support, exhibiting small to moderate effects. In adulthood, existing parents reported lower social support in the perinatal period compared to first‐time parents, *r* = −0.11 (95% CI −0.16, −0.06). In the immediate pre‐pregnancy period, parents with unplanned pregnancies reported lower social support in the perinatal period compared to parents with planned pregnancies, *r* = −0.13 (95% CI −0.18, −0.07). A preconception lifetime history of mental health disorder/s was also associated with lower levels of reported perinatal social support, *r* = −0.17 (95% CI −0.31, −0.02). Evidence did not support associations between perinatal social support and history of adverse obstetric outcomes, previous caesarean section, pre‐pregnancy substance use, pre‐pregnancy weight/body mass index (BMI), preconception care engagement, the use of assisted reproductive technology techniques to conceive or living with diabetes.

No meta‐analyses were able to be conducted within the relational level as data were insufficient. Two contextual level preconception antecedents were meta‐analysed, exhibiting small to moderate effects. In childhood and adolescence, parents who reported higher levels of ACEs reported lower levels of social support in the perinatal period, *r* = −0.22 (95% CI −0.30, −0.15). Parents who had migrated to a new country any time before next generation conception reported lower social support in the perinatal period compared to those who had not migrated, *r* = −0.11 (95% CI −0.16, −0.05). Sensitivity analyses revealed that all meta‐analytic effects were robust to a wide range of assumed within cluster correlations (rho values).

#### 5.3.3 Investigate Whether Meta‐Analytic Associations Between Preconception Antecedents and Perinatal Social Support Differ by Parent and Study Characteristics

Moderation analyses are presented in Supporting Information 2 (Tables S1–S12). Overall, Wald tests indicated no differences in the meta‐analytic findings according to parent gender, perinatal timing of social support measurement, parity of the sample, sample type (general population compared to selective/risk samples), or parent resident country income classification level. Moderation analyses were often limited by small cell sizes due to little available evidence. Furthermore, meta‐analytic associations were not found to differ by parent age at perinatal assessment or quality appraisal score, except for where increasing age strengthened the negative relationship between mental health disorders and perinatal social support (*β* = −0.07, 95% CI −0.11, −0.03, *p* = 0.016).

### 5.4 Aim 2: Aspects of Perinatal Social Support

Perinatal social support was commonly measured using validated questionnaires such as the Multidimensional Scale of Perceived Social Support (MSPSS; Zimet et al. 1988), Medical Outcomes Study (MOS) Social Support Survey (Sherbourne and Stewart 1991), and the Interpersonal Support Evaluation List (ISEL) and ISEL‐12 (Cohen and Hoberman 1983; Cohen et al. 1985). A number of studies used translations of existing measures (12%), adapted existing measures (17%), or devised items (28%) to measure perinatal social support. Few studies measured ‘actual received support’ (e.g., support network size, frequency of contact with support network; 10%). Most studies measured dimensions of ‘perceived’ support and common structural and functional aspects described below.

#### 5.4.1 Examine Whether Associations Between Preconception Antecedents and Perinatal Social Support Differ Depending on Measured Structural Aspects of Social Support

Data were sufficient to conduct moderation analyses for four individual and two contextual level antecedents. Results are presented in Table 2. Overall, Wald tests indicated no differences between partner, family, friends, health professionals, neighbourhood, and network sources of support in the meta‐analytic findings. Nevertheless, when we examined the associations within stratifications, social support from partners, family, and network support tended to be lower in parents having their second or subsequent child, *r* range = −0.07 to −0.17. Social support from partners also tended to be lower when pregnancy was unplanned, *r* = −0.22 (95% CI −0.31, −0.13). Moderation analyses were often limited by small cell sizes due to little available evidence.

**TABLE 2:** Moderation analyses examining preconception antecedents and common structural aspects of perinatal social support.

Sources of support	*k*	*e*	*r*	95% CI	*p*	Wald test (*p*)
Individual level antecedents						
*Parity*						
Partner	15	22	−0.07	(−0.14, 0.00)	0.049	0.076
Family	8	14	−0.17	(−0.24, −0.11)	0.001	
Friends	5	11	−0.08	(−0.18, 0.01)	0.072	
Health professionals	10	21	−0.04	(−0.10, 0.02)	0.201	
Neighbourhood	1	2	NA			
Network	6	11	−0.10	(−0.18, −0.03)	0.018	
*Pre‐pregnancy weight/BMI*						
Partner	1	1	NA			NA
Family	0	0	NA			
Friends	0	0	NA			
Health professionals	2	3	0.03	(−0.44, 0.51)	0.528	
Neighbourhood	0	0	NA			
Network	1	1	NA			
*Pregnancy intention (Unplanned)*						
Partner	8	10	−0.22	(−0.31, −0.13)	0.001	0.162
Family	5	8	−0.12	(−0.46, 0.21)	0.340	
Friends	3	4	−0.13	(−0.35, 0.08)	0.097	
Health professionals	3	4	−0.04	(−0.28, 0.20)	0.549	
Neighbourhood	0	0	NA			
Network	3	4	−0.05	(−0.09, 0.00)	0.056	
*Method of conception (Assisted reproduction)*						
Partner	1	6	NA			0.136
Family	2	24	0.00	(−0.53, 0.53)	0.997	
Friends	2	20	−0.07	(−0.30, 0.17)	0.177	
Health professionals	0	0	NA			
Neighbourhood	0	0	NA			
Network	1	16	NA			
Contextual level antecedents						
*ACEs*						
Partner	3	6	−0.06	(−0.19, 0.07)	0.116	0.286
Family	3	10	−0.30	(−0.78, 0.18)	0.095	
Friends	1	2	NA			
Health professionals	0	0	NA			
Neighbourhood	0	0	NA			
Network	2	2	−0.07	(−0.16, 0.01)	0.060	
*Migration history*						
Partner	7	9	−0.02	(−0.11, 0.07)	0.608	0.456
Family	4	7	−0.21	(−0.56, 0.15)	0.143	
Friends	3	4	−0.07	(−0.55, 0.40)	0.440	
Health professionals	3	7	0.00	(−0.06, 0.06)	0.829	
Neighbourhood	1	1	NA			
Network	2	2	−0.23	(−0.93 0.47)	0.148	

*Note: k* = number of studies, *e* = number of effects. Moderation analyses were conducted using studies included in the main meta‐analyses. Network support includes support network size and frequency of contact with support network. There was insufficient evidence to examine differences between structural aspects of perinatal social support and history of adverse obstetric outcomes, previous caesarean section, pre‐pregnancy substance use, preconception care engagement, history of mental health disorder/s, and living with diabetes.

#### 5.4.2 Examine Whether Associations Between Preconception Antecedents and Perinatal Social Support Differ Depending on Measured Functional Aspects of Social Support

Data were sufficient to conduct moderation analyses for three individual and two contextual level antecedents. Results are presented in Table 3. Overall, Wald tests indicated no differences between emotional, instrumental, informational, and appraisal support in the meta‐analytic findings. When we examined the associations within stratifications, there were no statistically significant associations between specific support functions and preconception antecedents. Moderation analyses were often limited by small cell sizes due to little available evidence.

**TABLE 3:** Moderation analyses examining preconception antecedents and common functions of perinatal social support.

Support functions	*k*	*e*	*r*	95% CI	*p*	Wald test (*p*)
Individual level antecedents						
*Parity*						
Emotional	5	6	−0.04	(−0.14, 0.07)	0.365	0.743
Instrumental	4	4	−0.05	(−0.25, 0.16)	0.457	
Informational	2	4	−0.24	(−1.83, 1.35)	0.305	
Appraisal	3	5	−0.09	(−0.38, 0.21)	0.291	
*Pregnancy intention (Unplanned)*						
Emotional	1	2	NA			NA
Instrumental	5	7	−0.18	(−0.48, 0.12)	0.173	
Informational	0	0	NA			
Appraisal	0	0	NA			
*Method of conception (Assisted reproduction)*						
Emotional	2	22	−0.03	(−0.53, 0.47)	0.583	0.325
Instrumental	2	22	−0.04	(−0.50, 0.43)	0.507	
Informational	0	0	NA			
Appraisal	0	0	NA			
Contextual level antecedents						
*ACEs*						
Emotional	3	3	−0.26	(−0.74, 0.23)	0.121	0.298
Instrumental	2	2	−0.41	(−0.93, 0.11)	0.064	
Informational	0	0	NA			
Appraisal	2	3	−0.21	(−0.78, 0.37)	0.138	
*Migration history*						
Emotional	5	10	−0.10	(−0.29, 0.09)	0.204	0.460
Instrumental	7	8	−0.04	(−0.22, 0.14)	0.587	
Informational	1	1	NA			
Appraisal	0	0	NA			

*Note: k* = number of studies, *e* = number of effects. Moderation analyses were conducted using studies included in the main meta‐analyses. There was insufficient evidence to examine differences between emotional, instrumental, informational, and appraisal support for history of adverse obstetric outcomes, previous caesarean section, pre‐pregnancy substance use, pre‐pregnancy weight/BMI, preconception care engagement, history of mental health disorder/s, and living with diabetes.

### 5.5 Aim 3: Summary of Evidence Gaps and Future Research Areas

Several evidence gaps, limitations, and opportunities for future research are considered in more detail in the discussion section. In summary, the literature to date examining preconception antecedents of perinatal social support has not yet substantially examined relational antecedents, protective/promotive antecedents, the potentially unique associations between antecedents and particular structural and functional aspects of perinatal social support, drawn on the methodological strengths of long‐term prospective cohort studies, or examined possibly distinct antecedents in particular sub‐populations of parents such as fathers, younger and older parents, and parents in Low‐ and Middle‐Income Countries.

## 6 Discussion

In taking a life course perspective to systematically identify preconception antecedents of perinatal social support, we identified 46 factors that had been the subject of empirical investigations published since 1986. In meta‐analyses of 12 of these antecedents, we found that lower levels of perinatal social support were associated with contextual level antecedents including a history of adverse childhood (and adolescent) experiences (ACEs) and lifetime history of migration to a new country. Lower levels of perinatal social support were also associated with individual level antecedents including lifetime history of mental health disorder/s, unplanned pregnancies and being an existing parent. We start by discussing ACEs, which showed the strongest association with perinatal social support and progress inwards through the ecological levels of influence from contextual to individual level antecedents. Overall, evidence synthesised in this review found that parents who experienced disruption to their developmental environment preconception, as early as childhood, may be at particular risk of inadequate social support perinatally; public health resourcing could be directed to those who have experienced such disruptions. Additionally, future perinatal interventions designed to strengthen social support could be responsive to parents' broader preconception histories.

### 6.1 Contextual Level Life Course Antecedents

#### 6.1.1 Adverse Childhood (And Adolescent) Experiences

Contextual level antecedents were among the most studied and showed the clearest effects with perinatal social support. ACEs are most strongly associated with lower perinatal social support, demonstrating a moderate effect, despite being the most distal preconception antecedent meta‐analysed. As most included studies did not distinguish between types of ACEs in analyses (e.g., physical, sexual or emotional abuse, neglect, exposure to family member substance use, death or incarceration, witnessing violence), in combination we deemed them to sit at the contextual level, but we acknowledge that many ACEs have relational components. Future research could consider the potentially differential effects of certain types of ACEs on social support in early parenthood.

Although ACEs were reported retrospectively in all included studies, other meta‐analytic evidence suggests that retrospective measures of ACEs may be more sensitive than prospective measures in capturing cases of childhood maltreatment (Baldwin et al. 2019). Broader contextual challenges may increase the risk of ACEs in children, such as lower childhood socioeconomic position and parent factors such as parent experience of ACEs and trauma (Narayan et al. 2021; Walsh et al. 2019). There is substantial evidence of the immediate and longer‐term developmental harms for children and young people who have experienced ACEs (Kerker et al. 2015; Lorenc et al. 2020). Experiencing multiple or chronic exposure to ACEs tends to have particularly cumulative negative effects on developmental outcomes (Hughes et al. 2017; Schneider et al. 2020).

The harmful role of ACEs continues well into adulthood as people transition into parenting the next generation (Spry et al. 2026). Early experiences of distress and trauma can disrupt neurodevelopment, escalating risk of structural and social disadvantage across the life course including material disadvantage, unhealthy behaviours, increased sensitisation to stress, increased chronic illnesses, reduced psychosocial resources, mental health problems, social isolation, and unstable and stressful relationships (Deighton et al. 2018; Giovanelli et al. 2020; Horan and Widom 2015; Nurius et al. 2016; Radey and Mcwey 2025; Schneider et al. 2020; Soares et al. 2021). Crucially, the negative flow on effects of ACEs presents challenges for sustained interpersonal relationships within which social support is provided. An increased likelihood of insecure attachments and negative cognitive distortions such as maladaptive schemas, shame, self‐criticism, hopelessness, and negative inferential style evident in many who have experienced ACEs, may negatively impact an individual's interpersonal orientation and subsequently connections with others (Aafjes‐Van Doorn et al. 2020; Pilkington et al. 2021; Ye et al. 2023; Yoo et al. 2014). Our meta‐analysis demonstrates that the ‘long reach’ of ACEs extends to the perinatal period and places individuals at risk of experiencing lower levels of social support during this time.

Moreover, the perinatal period may represent a critical period of vulnerability for people who have experienced early life trauma. Trauma symptoms may be triggered by changes experienced during pregnancy, childbirth, breastfeeding and coming into contact with the healthcare system (Chamberlain et al. 2019). Perceptions of social support may be negatively affected by feelings of fear, distrust and unsafety that may have been internalised preconception in the context of a lack of security in the developing years (Chamberlain et al. 2019). Those who have experienced ACEs may have complex and additional support needs in the perinatal period. Our findings support the need for trauma‐informed pregnancy and postpartum care (Clancy et al. 2020).

#### 6.1.2 Lifetime History of Migration

Our meta‐analyses suggested that lifetime history of migration to a new country is associated with lower perinatal social support. The number of people migrating worldwide continues to increase each year (International Organization for Migration and I. Organization 2024). Migrating may mean leaving behind existing social support networks and navigating structural challenges to forming new networks in a new country, with potential barriers including language differences, financial limitations, prejudice, discrimination, social isolation and exclusion (Boccagni 2015; Guruge et al. 2015; Urquia et al. 2012). Cultural differences in expectations around when, how and from whom to seek support may exist, and stigma may further prevent individuals from seeking support when needed (Guruge et al. 2015). Family members who remain in the country of origin may continue to be a source of support for individuals who have migrated, however, these relationships can become strained over time (Boccagni 2015). Migrants may require more support to deal with specific needs related to moving to a new country such as acculturation stress, language difficulties, navigating new healthcare and education systems, securing employment, and coping with discrimination (Guruge et al. 2015). Some migrant mothers report having to rely heavily on their partners and health professionals for support in the absence of having family and friends locally, with some migrant fathers acknowledging that they likely play a more active support role in the perinatal period than if they had not migrated (Owens et al. 2016; Renzaho and Oldroyd 2014; Riggs et al. 2016). Many parents report that early parenthood can be lonely, particularly when there are language barriers, conflicting cultural identities and geographical distance from friends and family (Owens et al. 2016; Renzaho and Oldroyd 2014; Riggs et al. 2016).

Improving access to and the availability of social support in migrant populations is important as it buffers acculturation stress and is protective of mental health and wellbeing from adolescence through to the transition to parenthood (Anderson et al. 2017; Fellmeth et al. 2017; Sirin et al. 2013). Our findings support the need for culturally sensitive and competent perinatal healthcare to support new migrant parents (Forbes et al. 2021, 2023). While not explored here, age of migration, country of origin, country migrated to, time since migration, reasons for migrating or seeking asylum, and refugee status may exacerbate or attenuate this relationship, representing different support needs in the perinatal period across levels of social advantage and disadvantage (Elshahat et al. 2022; Urquia et al. 2012; Wong and Edwards 2023).

### 6.2 Relational Level Life Course Antecedents

Very few studies examined associations between relational level antecedents and perinatal social support. No relational antecedents were able to be meta‐analysed due to a lack of available evidence. However, contextual antecedents captured in this review overlap with and have relational components. For example, many experiences of ACEs include inherently relational aspects, such as abuse or neglect by a caregiver or trusted adult. Nevertheless, the lack of investigations into relational antecedents is surprising given that perinatal social support is itself a relational construct. In the perinatal period, examples of social support may include an attentively listening and reassuring partner or receiving a pre‐cooked meal from a family member or friend. The quantity, quality, and strength of pre‐existing social ties is likely to inform parents' experiences of social support during early parenthood. Preconception relational antecedents that have shown long‐lasting impacts warrant further investigation. For example, evidence suggests the quality of relationships adolescent girls and boys have with their parents and peers is associated with perinatal outcomes (Aarsman et al. 2026; Macdonald et al. 2018, 2021).

### 6.3 Individual Level Life Course Antecedents

#### 6.3.1 Lifetime History of Mental Health Disorder/s

Most preconception antecedents meta‐analysed were individual level factors, and several were associated with lower perinatal social support. A history of experiencing mental health disorder/s any time before next generation conception is associated with lower perinatal social support. We also found some evidence that this association may be exacerbated as parents age. Mental health has long‐term effects over development, including the transition to parenthood and into the next generation (Letcher et al. 2009, 2020; Macdonald, Greenwood, et al. 2022; Spry et al. 2021). Symptoms of mental health disorders, including negative attribution bias, may place significant strain on individuals' interpersonal relationships, limit motivation and opportunities for socialising, and increase feelings of social isolation across the life course (Chopik et al. 2023; Mansour et al. 2021; Xu et al. 2018). Additionally, individuals experiencing mental health problems may have difficulties in eliciting and activating support and may have greater support needs. More work is needed to understand the associations between perinatal social support and lifetime history of mental health disorders beyond depression and anxiety (Asselmann et al. 2016; Spry et al. 2021). Our findings support the ongoing priority of mental health in public health interventions and programs, at all ages and stages of the life course.

#### 6.3.2 Unplanned Pregnancy

Pre‐pregnancy individual level antecedents were the most studied. Our meta‐analysis indicates that unplanned pregnancy is associated with lower perinatal social support. We found that support from a partner in particular during the perinatal period tended to be lower when the pregnancy was unplanned. Unplanned pregnancies represent a significant proportion of pregnancies worldwide and are linked to higher rates of postpartum mental health problems in both mothers and fathers (Bearak et al. 2022; Smith et al. 2023; Qiu et al. 2020). The unplanned nature of the pregnancy may mean that individuals have not had the opportunity to build and activate their social support systems in preparation for the perinatal period when support is in high demand. Unplanned pregnancy may also induce feelings of resentment within intimate partner relationships, which may compound a normative decline in relationship quality over the transition to parenthood, negatively affecting perceptions and availability of partner support (Doss et al. 2009; Smith et al. 2022).

Moreover, unintended pregnancy may be a response to a lack of social support before next generation conception. Prospective longitudinal research suggests that parents' reproductive and parenting identity is predicted by their social ecology before parenthood (Macdonald et al. 2023). Young adult social support and relationship quality with family and peers inform whether individuals become parents and the timing of parenthood (Macdonald et al. 2023). Young people with sub‐optimal preconception social ecologies may be more likely to have unplanned pregnancies (Macdonald et al. 2023). Moreover, individuals whose preconception social networks mainly consist of weak ties, characterised by low emotional closeness and short duration of relationships, are more likely to unintentionally become parents compared to those with stronger ties (Lois 2016). Social support may also play a role in contraception use (Moseson et al. 2018). Our findings support the importance of investment in preconception care and family planning programs and interventions.

#### 6.3.3 Parity

Finally, meta‐analysis demonstrated that having a second or subsequent child is associated with lower perinatal social support. In particular, we found that support from partners and family, and network support, during the perinatal period tended to be lower for existing parents. Social networks tend to shrink around the transition to parenthood, perhaps due to reduced time or energy to foster these relationships as well as shifting priorities towards raising a family (Asselmann et al. 2016; Bost et al. 2002; Wrzus et al. 2013). The size of parents' social networks continues to reduce over time, following a normal developmental path where emotionally meaningful relationships are prioritised over more peripheral relationships (Lang and Carstensen 2002; Wrzus et al. 2013). Thus, multiparous parents may have reduced access to informal social support. Similarly, existing parents may be often overlooked in healthcare systems which focus on formally supporting new parents in the transition to parenthood (Smith et al. 2023). Family and friends may assume that since parents have ‘done this before’ they may require less support. However, parents with multiple children, including parents with multiple births, may require more support due to the additional emotional, financial, and practical challenges of raising more than one child. Our findings highlight that existing parents report feeling less supported than first time parents. Parents may benefit from ongoing and increased support in their subsequent perinatal periods (McLeish and Redshaw 2017).

### 6.4 Limited Evidence and Evidence Gaps

Based on evidence reported from 10 studies, our meta‐analytic findings suggest that pre‐pregnancy weight/BMI is not associated with social support experiences in the perinatal period. The remaining meta‐analyses, based on five studies or less, did not support that a history of adverse obstetric outcomes, previous caesarean section, pre‐pregnancy substance use, preconception care engagement, method of conception, or living with diabetes are associated with social support in the perinatal period. Several other individual (e.g., medical and health factors, personality, specific mental health problems), relational (e.g., attachment, social support, relationship quality), and contextual (e.g., socioeconomic position, family environment) level preconception antecedents included in this review may be associated with perinatal social support but were unable to be meta‐analysed due to insufficient data. More evidence is needed to determine what role, if any, these antecedents play in parents' social support during the perinatal period (Biden et al. 2026).

Furthermore, the antecedents of perinatal social support captured in this review tended to represent risk. In many cases, the absence of a risk factor does not indicate the presence of a protective factor. To inform strength‐based interventions, research is needed to understand the modifiable life course factors which might specifically promote perinatal social support (e.g., protective and compensatory childhood experiences [PACEs] including community engagement, physical activity, empathy and school bonding; Mitic et al. 2021; Morris and Hays‐Grudo 2023).

### 6.5 Strengths and Limitations

A key strength of this review is its scope. We placed no limits on the life course antecedents of interest and thus were able to include a broad range of constructs spanning developmental timing preconception, from childhood to pre‐pregnancy. This also allowed us to capture antecedents from individual, relational and contextual ecological levels, together representing an array of factors which influence social development proximally and distally over both developmental timing and ecological level. This review is further strengthened by adhering to principles of open science, including preregistration of the review protocol and provision of open access data and meta‐analytic code.

We only found a small amount of evidence of publication bias. This is perhaps due to a large number of studies incidentally reporting the sometimes small or non‐significant associations between perinatal social support and a preconception antecedent, whilst focusing on another outcome. That being said, a number of studies were excluded because whilst they measured perinatal social support and a preconception antecedent, they did not report enough information for us to determine the relationship between the two or they did not report estimates for non‐significant associations. We did not contact authors due to the large number of articles where this was the case. The impact of excluding this literature on the representativeness of the evidence base is unknown. We also aimed to minimise publication bias by including grey literature. Nevertheless, it is likely that some publication bias may remain, particularly in the group of studies that investigated ACEs, therefore we advise that this effect size be interpreted with caution as it may be inflated. Although we accounted for estimates nested within studies (i.e., when studies reported multiple effects) in each of the meta‐analyses, some participant overlap remained across manuscripts.

Included studies varied on indicators of study quality. Many included studies performed poorly on indicators of measurement bias due to their reliance on retrospective assessment of antecedents. When preconception antecedents were measured prospectively, data were mostly collected from medical records. Data linkage methods and retrospective measures are limited in their ability to capture psychosocial processes over time which are likely to underpin later experiences of social support (Kretschmer 2021). Multi‐generation prospective cohort studies are uniquely placed to investigate social development across the life course and transition to parenthood and could be drawn on in future research (Kretschmer 2021; Olsson et al. 2022; Spry et al. 2020).

This review was unable to determine the cumulative effects or the interaction of preconception antecedents (such as experiencing mental health problems, unplanned pregnancy and already having children; Smith et al. 2023) on later experiences of social support during parenthood. Future work could consider the interdependence of life course experiences at the intersections of developmental timing and ecological level (Bernardi et al. 2019). Qualitative research could also help to capture the complexities of preconception experiences preceding perinatal social support.

In regard to perinatal social support, the included studies reflect the predominant focus on self‐report measures of perceived support in the literature in comparison to more objective measures of ‘actual received support’. Although heterogeneous, the vast majority of measures were assessed as being clearly defined, valid and reliable, increasing our confidence that they measured the same underlying construct of perceived social support. We found little evidence of differences in the meta‐analytic associations by structural and functional aspects of social support; however, many of the moderation analyses had limited statistical power due to small cell sizes and little existing evidence. The antecedents of certain types of social support specific to early parenthood, such as support for breastfeeding, caregiving and childbirth, warrant further investigation given their potential impact on postpartum wellbeing (Wadephul et al. 2020).

Importantly, the majority of included studies sampled mothers, aged between 20 and 34 years, residing in high income countries. The findings of our review may not be generalisable beyond this demographic. This review was unable to detect any gender differences in antecedents of perinatal social support as fewer than 10% of included studies sampled and reported effects separately for fathers. Further, no unique preconception antecedents were investigated in the literature in samples of fathers. Young men are not generally socialised as future parents and tend to report little consideration of their potential parenting role (Kings et al. 2017). There are likely to be considerable gender differences in perinatal social support experiences given the substantially reduced levels of social connections of men as they enter the peak age for parenthood (Mansour et al. 2023; Marabel‐Whitburn et al. 2023). Moreover, formal support and healthcare structures typically do not provide adequate care for men in the perinatal period despite their motivation (Macdonald, Mansour, et al. 2022; Wynter et al. 2024; Xavier et al. 2024). Similarly, younger and older parents were rarely sampled in included studies, but may have distinct antecedents of perinatal social support given their own developmental timing of parenthood (Frewin et al. 2007; McMahon et al. 2011; Mehta et al. 2020; Saxbe et al. 2018). More research is also needed to determine the antecedents of perinatal social support in Low‐ and Middle‐Income Countries (LMIC; The World Bank 2022), and in other priority populations who may face challenges or require additional support during early parenthood.

### 6.6 Implications and Translation

We found evidence that parents' developmental life histories play a role in their experiences of social support during pregnancy and the early postpartum periods, particularly adverse preconception and interconception experiences in increasing later vulnerability. Antecedents from across the life course, from as early as childhood to as proximally as pre‐pregnancy, were associated with lower levels of social support in early parenthood. Given that several antecedents occurred distally to the perinatal periods, small to moderate effect sizes found have meaningful implications for public health (Götz et al. 2022). Observed effects may have an even larger impact within perinatal care contexts when scaled up over time and to the population level, particularly for those most at risk of inadequate social support (Carey et al. 2023; Funder and Ozer 2019). Overall, our findings support that social support in the perinatal period is likely multidetermined, by diverse accumulating and cascading influences across the life course well before parenthood.

Our meta‐analytic findings point to ‘who’ may be in most need of assistance in accessing social support during the perinatal period. Expecting parents who have previously experienced psychosocial trauma, distress or significant disruption may benefit from targeted interventions that seek to provide and link them to social supports (Lyu et al. 2023; Stewart et al. 2015). Nursing and other healthcare practitioners should consider when making referrals to support services, that the support is appropriate and responsive to parents' developmental contexts, which precede the immediate pre‐pregnancy period and span interconception experiences. Practitioners should continue to consider pregnancy planning and lifetime history of ACEs and mental health problems during antenatal assessments. Findings also reinforce that migrant parents, existing parents, and fathers and non‐birthing parents may benefit from targeted interventions that are tailored to their specific support needs and developmental experiences.

## 7 Conclusion

Our findings add to a growing body of literature that identifies parents' preconception developmental life experiences, from as early as childhood and across the gamut of individual and contextual levels, as contributing to outcomes in early parenthood. Parents who experienced disruption to their developmental environment in the preconception or interconception periods may be at particular risk of inadequate social support perinatally. Furthermore, existing parents may be a somewhat forgotten group who are also at risk of experiencing lower levels of support during their subsequent perinatal periods. Nursing and other healthcare practitioners should keep in mind that these parents may benefit from additional support as early as possible and work collaboratively with patients to recommend appropriate supports that are responsive to parents' broader preconception and interconception histories. Our review also documents multiple gaps in the literature; particularly the need for future research utilising high‐quality prospective evidence to understand the relational origins of perinatal social support, and research in Low‐ and Middle‐Income Countries where experiences of social support and individual and contextual vulnerabilities may differ from within high income country settings. Future research could implement causal methodology to identify potential intervention targets in the preconception period to benefit young people at the time and their future social support should they become parents.

## Author Contributions

Conceptualization: all authors, led by E.J.B., J.A.M., E.A.S., C.A.O., C.J.G., P.L., L.M.F., K.A.M., L.G.G. Project coordination: E.J.B. Data collection and curation: all authors, led by E.J.B., J.A.H., M.N.G., S.H., A.S. and S.Y.Z. Formal analyses: Led by EB and supported by C.J.G. Data visualisation and interpretation: all authors, led by E.J.B., J.A.M., E.A.S., C.J.G. and P.L. Writing original draft: Led by E.J.B. and supported by J.A.M., E.A.S., C.J.G. and P.L. All authors contributed to additional drafts of the manuscript. Critically reviewing final manuscript: all authors.

## Funding

E.J.B. was supported by a Deakin University Postgraduate Research Scholarship. E.A.S. and C.J.G. were supported by Deakin University Postdoctoral Research Fellowships. J.A.M. was supported by a Medical Research Future Fund Million Minds Mental Health Research Grant (MRF2026823). C.A.O. was supported by a National Health and Medical Research Council Investigator Grant (APP1175086). Research at the Murdoch Children's Research Institute was supported by the Victorian Government's Operational Infrastructure Program.

## Conflicts of Interest

The authors declare no conflicts of interest.

## Supporting information


**Data S1:** Supporting Information 1.

**Table S1:** Meta‐analytic associations between perinatal social support and history of adverse obstetric outcomes by parent and study characteristics.
**Table S2:** Meta‐analytic associations between perinatal social support and parity by parent and study characteristics.
**Table S3:** Meta‐analytic associations between perinatal social support and previous caesarean section by parent and study characteristics.
**Table S4:** Meta‐analytic associations between perinatal social support and pre‐pregnancy substance use by parent and study characteristics.
**Table S5:** Meta‐analytic associations between perinatal social support and pre‐pregnancy weight/body mass index (BMI) by parent and study characteristics.
**Table S6:** Meta‐analytic associations between perinatal social support and unplanned pregnancy by parent and study characteristics.
**Table S7:** Meta‐analytic associations between perinatal social support and preconception care engagement by parent and study characteristics.
**Table S8:** Meta‐analytic associations between perinatal social support and assisted reproduction by parent and study characteristics.
**Table S9:** Meta‐analytic associations between perinatal social support and history of mental health disorder/s by parent and study characteristics.
**Table S10:** Meta‐analytic associations between perinatal social support and living with diabetes by parent and study characteristics.
**Table S11:** Meta‐analytic associations between perinatal social support and adverse childhood and adolescent experiences by parent and study characteristics.
**Table S12:** Meta‐analytic associations between perinatal social support and migration history by parent and study characteristics.

## Data Availability

The data that support the findings of this study are openly available at https://doi.org/10.17605/OSF.IO/AZVUE.
