# Supplementary material for: Social Support in the Perinatal Period: A Systematic Review and Meta‐Analysis of Life Course Antecedents
Source: J Adv Nurs. 2026 Jul 28;82(12):10567–86. doi: 10.1111/jan.70677 (PMC13752662; doi:10.1111/jan.70677)
Supplement: Supplementary file 1 [file JAN-82-10567-s001.pdf]

Social Support in the Perinatal Period:

A Systematic Review and Meta-analysis of Life Course Antecedents (Biden et al., 2026)

Supplementary Material

Table of Contents

Search strategy 2

Inclusion and exclusion criteria 5

Additional data extraction, synthesis & analysis information 6

Data extraction 6

Preconception antecedent groupings for meta-analysis 6

Moderator coding 7

Social support coding 7

Quality appraisal 9

PRISMA flow diagram 10

Forest plots 11

Funnel plots 12

Reference list of included studies 13

Additional references 27

Search strategy

Concepts - *combined using AND*	Search terms, index search terms, and search syntax for <u>electronic database search</u>
**Social support**	**PsycINFO, Medline Complete & CINAHL Complete**<br>*Title and abstracts searched for:* (support) N2 (social OR network* OR perceived OR received OR emotion* OR informal OR formal OR available OR instrumental OR informational OR apprais* OR structural OR functional OR tangible OR esteem OR companionship OR financial OR material OR practical OR work* OR housing OR childcare OR health* OR access OR psychosocial OR partner* OR spous* OR "significant other" OR husband* OR wife* OR friend* OR family OR peer OR community)<br><br>OR<br><br>PsycINFO – DE "social support"<br><br>Medline Complete – (MH "social support+")<br><br>CINAHL Complete – (MH "psychosocial support")<br><br>**Embase**<br>((support) NEAR/2 (social OR network* OR perceived OR received OR emotion* OR informal OR formal OR available OR instrumental OR informational OR apprais* OR structural OR functional OR tangible OR esteem OR companionship OR financial OR material OR practical OR work* OR housing OR childcare OR health* OR access OR psychosocial OR partner* OR spous* OR 'significant other' OR husband* OR wife* OR friend* OR family OR peer OR community)):ti,ab<br><br>OR<br><br>'social support'/de
**The perinatal period**	**PsycINFO, Medline Complete & CINAHL Complete**<br>*Title and abstracts searched for:* perinatal* OR peri?natal OR neonatal* OR peripartum OR intrapartum OR puerperium OR puerperal* OR postnatal* OR post?natal OR postpart* OR antenatal* OR ante?natal OR antepartum OR prenatal* OR pre?natal OR preg* OR trimester* OR gestation* OR reproduct*<br><br>OR<br><br>PsycINFO – DE ("pregnancy" OR "postnatal period") (explode pregnancy)<br><br>Medline Complete – (MH "pregnancy") OR (MH "postpartum period")<br><br>CINAHL Complete – (MH "pregnancy") OR (MH "postnatal period")<br><br>**Embase**<br>(perinatal* OR peri?natal OR neonatal* OR peripartum OR intrapartum OR puerperium OR puerperal* OR postnatal* OR post?natal OR postpart* OR antenatal* OR ante?natal OR antepartum OR prenatal* OR pre?natal OR preg* OR trimester* OR gestation* OR reproduct*):ti,ab<br><br>OR<br><br>('pregnancy'/exp OR 'perinatal period'/de)
**Parents**	**PsycINFO, Medline Complete & CINAHL Complete**<br>*Title and abstracts searched for:* parent* OR matern* OR patern* OR mother* OR father* OR mom* OR mum* OR dad*<br><br>OR<br><br>PsycINFO – DE "parents" (explode)

	Medline Complete – (MH “parents+”)<br><br>CINAHL Complete – (MH “parents+”)<br><br>**Embase**<br>(parent* OR matern* OR patern* OR mother* OR father* OR mom* OR mum* OR dad*):ti,ab<br><br>OR<br><br>‘parent’/exp
**Preconception antecedents**	**PsycINFO, Medline Complete & CINAHL Complete**<br>*Title and abstracts searched for:* preconception OR pre-conception OR prepregnancy OR pre-pregnancy OR periconception OR predictor* OR predispos* OR antecedent* OR protective OR vulnerabilit* OR risk OR precipitat* OR exposure* OR life* OR “human development*” OR adult* OR adolescen* OR child* OR longitudinal OR prospective OR intergeneration* OR transgeneration* OR cross-generation*<br><br>OR<br><br>PsycINFO – DE (“longitudinal studies” OR “human development”) (explode)<br><br>Medline Complete – (MH “longitudinal studies+”) OR (MH “human development+”)<br><br>CINAHL Complete – (MH “prospective studies+”) OR (MH “human development+”)<br><br>**Embase**<br>(preconception OR pre-conception OR prepregnancy OR pre-pregnancy OR periconception OR predictor* OR predispos* OR antecedent* OR protective OR vulnerabilit* OR risk OR precipitat* OR exposure* OR life* OR ‘human development*’ OR adult* OR adolescen* OR child* or longitudinal OR prospective OR intergeneration* OR transgeneration* OR cross-generation*):ti,ab<br><br>OR<br><br>(‘longitudinal study’/exp OR ‘prospective study’/de OR ‘human development’/exp)
**Limiters**<br>• English<br>• Academic Journals (PsycINFO, Medline Complete & CINAHL Complete)<br><br>• Untick “apply equivalent subjects” (PsycINFO, Medline Complete & CINAHL Complete)<br>• Untick “mapping” (Embase)	

Descriptive summary: To capture various structural and functional aspects of social support, we searched for “support” near key terms such as “social”, “emotion*” and “partner*”. Search terms for the perinatal period included “perinatal*”, “postnatal*”, “antenatal*”, “preg*”, “postpart*”, etc., to cover a range of descriptors commonly used when referring to stages within this period. We used the gender-neutral term “parent*”, as well as gender specific terms “mother*”, “father*”, etc., to narrow the search to our population of interest. To capture a variety of antecedents occurring any time before next generation conception, we used broad terms such as “antecedent*”, developmentally specific terms including “preconception”, “prepregnancy”, “child*”, “adolescen*”, and methodological terms, for example “longitudinal”, “prospective”.

**Concepts -** *combined using AND*	**Search terms, index search terms, and search syntax for <u>grey literature search</u>**
**Social support**	**PsycEXTRA and Open Dissertations**<br>*Title and abstracts searched for:* (support) N2 (social OR network* OR perceived OR received OR emotion* OR informal OR formal OR available OR instrumental OR informational OR apprais* OR structural OR functional OR tangible OR esteem OR companionship OR financial OR material OR practical OR work* OR housing OR childcare OR health* OR access OR psychosocial OR partner* OR spous* OR "significant other" OR husband* OR wife* OR friend* OR family OR peer OR community)<br><br>OR<br><br>PsycEXTRA – DE "social support"
**The perinatal period**	**PsycEXTRA and Open Dissertation**<br>*Title and abstracts searched for:* perinatal* OR peri?natal OR neonatal* OR peripartum OR intrapartum OR puerperium OR puerperal* OR postnatal* OR post?natal OR postpart* OR antenatal* OR ante?natal OR antepartum OR prenatal* OR pre?natal OR preg* OR trimester* OR gestation* OR reproduct*<br><br>OR<br><br>PsycEXTRA – DE ("pregnancy" OR "postnatal period") (explode pregnancy)
**Parents**	**PsycEXTRA and Open Dissertations**<br>*Title and abstracts searched for:* parent* OR matern* OR patern* OR mother* OR father* OR mom* OR mum* OR dad*<br><br>OR<br><br>PsycEXTRA – DE "parents" (explode)
**Preconception antecedents**	**PsycEXTRA and Open Dissertations**<br>*Title and abstracts searched for:* preconception OR pre-conception OR prepregnancy OR pre-pregnancy OR periconception OR predictor* OR predispos* OR antecedent* OR protective OR vulnerabilit* OR risk OR precipitat* OR exposure* OR life* OR "human development*" OR adult* OR adolescen* OR child* OR longitudinal OR prospective OR intergeneration* OR transgeneration* OR cross-generation*<br><br>OR<br><br>PsycEXTRA – DE ("longitudinal studies" OR "human development") (explode)
**Limiters**<br>• Untick "apply equivalent subjects"	

**OPENGREY**<br>("social support" OR "support network") (pregnancy OR antenatal OR prenatal OR postpartum OR postnatal) (parent OR mother OR father) (predictor OR exposure OR preconception OR "life course" OR longitudinal OR prospective)<br><br>lang:"en"
**Google & Google Scholar**<br>("social support" OR "support network") (pregnancy OR antenatal OR prenatal OR postpartum OR postnatal) (parent OR mother OR father) (predictor OR exposure OR preconception OR "life course" OR longitudinal OR prospective)<br><br>Google - ext:pdf

Inclusion and exclusion criteria

Inclusion criteria

1. Available in English
2. Human sample: parents (including non-biological parents)
3. Published literature containing original empirical findings
4. Quantitative data analysing an association between perinatal social support and a preconception antecedent, or reporting sufficient data to determine the correlation between perinatal social support and a preconception antecedent
5. Measures social support (including any aspects of structure/function, perception or actual received support)
    - In mothers, fathers and/or parents (i.e., at the individual level)
    - During or relating to pregnancy (any) or the first 18 months postpartum (inclusive)
    - Including retrospective measures
6. Measures a preconception antecedent
    - Any variable (or level of a variable) clearly measured during or relating to any time before next generation conception
    - Including variables measured during or relating to the interception period/s
    - Including retrospective measures

Exclusion criteria

1. Studies measuring perinatal social support in the context of abortion, miscarriage, difficulty conceiving, loss, bereavement and/or grief relating to the death of the target offspring
2. Studies which measure constructs adjacent to the experience of social support and are beyond the scope of this review such as support needs, expectations, desires, preferences or concerns, simply having someone present during childbirth, receipt or utilisation of healthcare or social services, co-parenting or parental involvement, social isolation, coping or changes in support over time
3. Studies/effects relating to the “provision” of perinatal social support by the focal parent

Additional data extraction, synthesis & analysis information

Data extraction

Data extracted from each study included study characteristics and methods (parent resident country, sampling and participant recruitment information, eligibility criteria, parity of sample, sample size/power justification, attrition statistics, and details of handling missing data if provided); parent gender; antecedent measure, assessment method, and timing; outcome measure, assessment method, and timing; analytic sample size and estimates of association.

During data extraction, information that was missing was coded as not reported (NR). When effect sizes of interest were only reported with adjustments, individual study authors were contacted and asked to provide unadjusted associations for inclusion in the meta-analyses. Authors were given two weeks to respond. Nine authors were contacted and one author supplied the requested data.

Estimates of association that were unadjusted and from the highest level of analysis reported were selected to be extracted and converted to Pearson *r* correlations (if not already reported as a correlation). If studies did not report sufficient information for associations to be converted to Pearson *r* correlations, they were excluded. When applicable, the direction of coefficients were made consistent across studies in each meta-analysis to ensure coherent interpretation (i.e., some effects were “flipped” from negative to positive and vice versa).

When only raw data were reported for ordinal antecedents, the data endpoints were chosen for calculation of Pearson *r* correlations (e.g., for pregnancy intention, planned vs unplanned, not any levels in between). When only raw data were reported for nominal exposures, groups were either collapsed (e.g., for migration history, collapsing country of birth to represent “overseas”) or all possible relationships were calculated (e.g., for history of mental health disorder/s, anxiety and depression separately). When only raw data were reported ordinally for social support, the data end point which represented the most meaningful level of support was selected (e.g., adequate perceived support, no one to talk to, lowest quartile of support).

The relationships between an antecedent and perinatal social support were extracted for all subscales and total social support measures. Effects were only extracted from the item level of support measures if this was the only data available.

Preconception antecedent groupings for meta-analysis

In total, twelve separate meta-analyses were conducted. Predictor constructs were grouped for meta-analysis as follows: **history of adverse obstetric outcomes** including pregnancy complications, miscarriage, stillbirth; **parity** including number of children, births and pregnancies (note that measures were often not

described clearly enough to distinguish between parity and gravidity); **pre-pregnancy substance use** including alcohol, tobacco and concurrent alcohol and tobacco use; **pre-pregnancy weight/body mass index (BMI)** including weight, BMI and history of obesity; **pregnancy intention** including pregnancy intendedness, wanted-ness, planning and timing; **method of conception** including the use of assisted reproductive technology techniques compared to spontaneous conception; **history of mental health disorder/s** including anxiety, depression and comorbid anxiety and depression; **migration history** including country of birth and migrant status; **adverse childhood experiences** (ACEs) including physical, sexual and emotional abuse, physical and emotional neglect, as well as broader household and community challenges (e.g., exposure to parental substance use, death or incarceration, witnessing violence, and witnessing or being involved in accidents, war or natural disasters; as defined in Afifi, 2020; Kubany et al., 2000). Note that ACEs were generally reported as "total" combined measures of ACEs. Although many ACEs have relational components, in combination (as operationalised by most included studies) we deem them to sit at the *contextual level*.

Moderator coding

Parent and study characteristics included: parent gender (mothers, fathers); parent age (continuous); perinatal timing of social support measurement (pregnancy, postpartum, mixed); sample parity (primiparous, multiparous, mixed); sample type (general population, selective/risk); parent resident country income classification (high, upper-middle, lower-middle, low); and quality appraisal score (continuous).

Parent resident country income classification level was coded based on the World Bank Classifications for the 2021-2022 financial year (The World Bank, 2022).

Parent age at perinatal assessment was coded as the mean age (continuous), or if mean was not reported, the midpoint of the age range. If age was only reported as dichotomised categories, we selected the cut point as the mean. If the age range was open ended (i.e. 18+ years), we coded the mean age as "not reported" (NR). If age was reported as group means, we calculated the weighted average.

Sample type was coded as selective if any specific parent/infant demographic characteristics or risk factors were recruited for or were part of the participant eligibility criteria (e.g., under 18 years of age, low income, living in rural or remote areas, pre-existing medical condition, very low birth weight infants). If not, the study sample was coded as representing the general population.

Social support coding

Social support was coded according to both the structural and functional aspects highlighted in the study naming of the variable and measure/subscale/item used. Aspects of social support included: structural

support (partner, family, friends, health professionals, neighbourhood, and network); and functional support (emotional, instrumental, informational, and appraisal).

For structural aspects/sources of support**, partner support** included spouse, husband, significant other, etc. **Family** included kin, parents and in-laws. **Friends** included friends and confidants. **Health professionals** included midwives, maternal and child health nurses, general practitioners and doulas. **Neighbourhood** included specific mention of “neighbourhood”. **Network** included network size and frequency of contact. **Total** included when social support from multiple sources were assessed reflected in a single effect size. For functions of support, **emotional support** included belonging and affectional. **Instrumental support** included tangible, practical, “help”, and childcare specific. **Informational support** included specific mention of “informational”. **Appraisal support** included self-esteem, affirmational, and goal related. **Total** included when multiple functions of social support were reflected in a single effect size. Where sufficient information was available social support was coded for both source and function.

Quality appraisal

We adapted the National Institutes of Health Quality Assessment Tool for Observational Cohort and Cross-Sectional Studies to assess study quality (National Heart Lung and Blood Institute: National Institutes of Health, 2014). We used the original item numbers as a reference for coding the data. We omitted items only relevant to causal research questions and regarding outcome assessor blinding as they were not applicable to this review.

Quality appraisal indicator domain & coding	Quality appraisal indicator items
1	Was the research question or objective in this paper clearly stated?
**Generalisability**	
2	Was the study population clearly specified and defined?
**Selection**	
13	Was loss to follow-up after baseline 20% or less?
3	Was the participation rate of eligible persons at least 50%?
4	Were all the subjects selected or recruited from the same or similar populations (including the same time period)? Were inclusion and exclusion criteria for being in the study prespecified and applied uniformly to all participants?
“Missing”	Does the study mention missing data and account for how they were treated in the analysis? Studies that remove incomplete data from the outset and do not include in the total *N* are considered meeting the criteria for addressing missing data* (Spry et al., 2020; Madigan et al., 2018)
**Measurement**	
6a	Were the exposure/s of interest measured prospectively?*
6b	Were the outcome/s of interest measured prospectively?*
9	Were the exposure measures (independent variables) clearly defined, valid, reliable, and implemented consistently across all study participants?
10	Was the exposure(s) assessed more than once over time?
11	Were the outcome measures (dependent variables) clearly defined, valid, reliable, and implemented consistently across all study participants?
**Power**	
5	Was a sample size justification or power description provided?*

**Adapted items*

PRISMA flow diagram

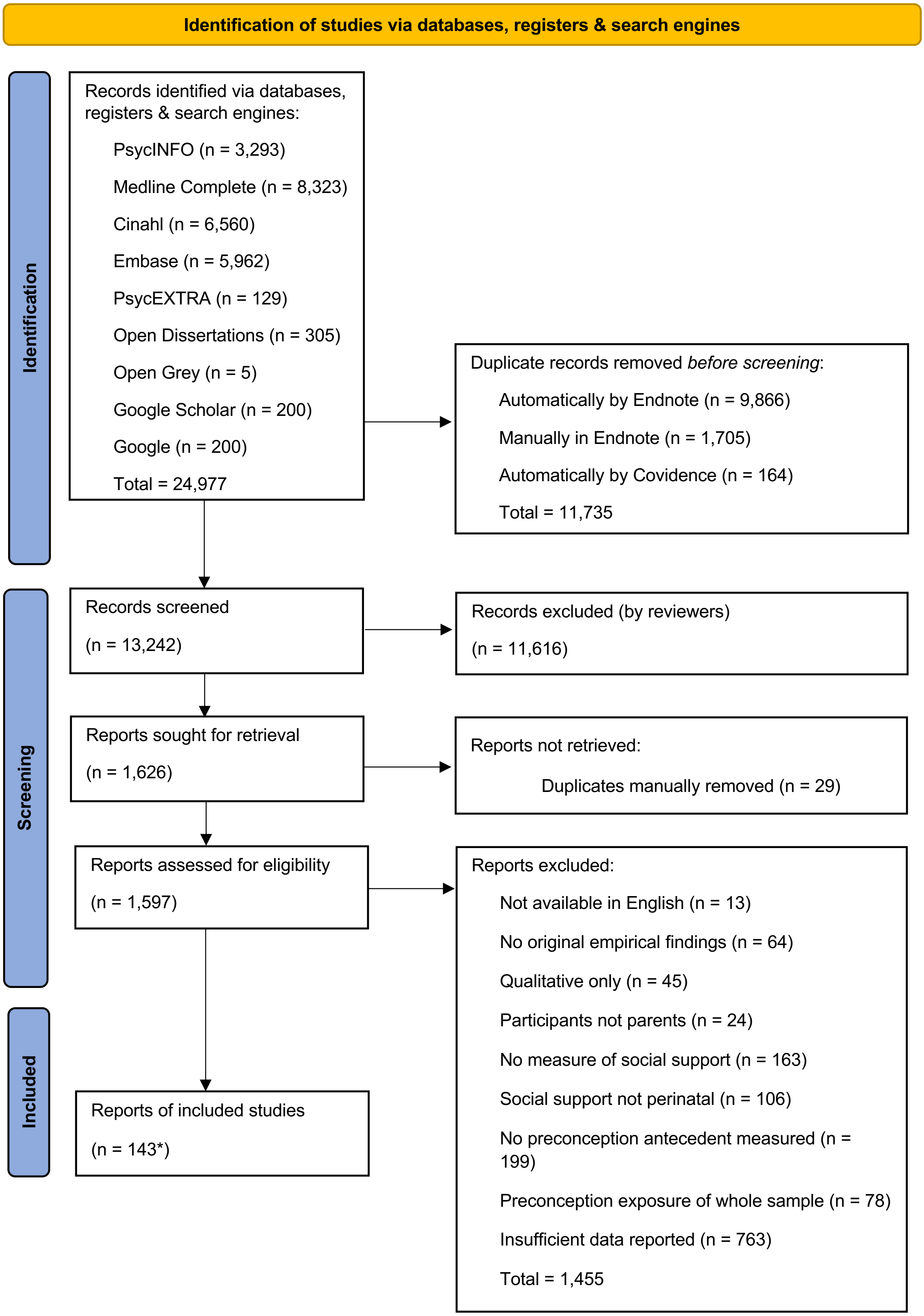

**Note: One study was identified through researcher networks outside of the systematic search*

Forest plots

i. History of adverse obstetric outcomes and perinatal social support
ii. Parity and perinatal social support
iii. Previous caesarean section and perinatal social support
iv. Pre-pregnancy substance use and perinatal social support
v. Pre-pregnancy weight/body mass index (BMI) and perinatal social support
vi. Pregnancy intention (unplanned) and perinatal social support
vii. Preconception care engagement and perinatal social support
viii. Method of conception (assisted reproduction) and perinatal social support
ix. History of mental health disorder/s and perinatal social support
x. Living with diabetes and perinatal social support
xi. Adverse childhood experiences (ACEs) and perinatal social support
xii. Migration history and perinatal social support

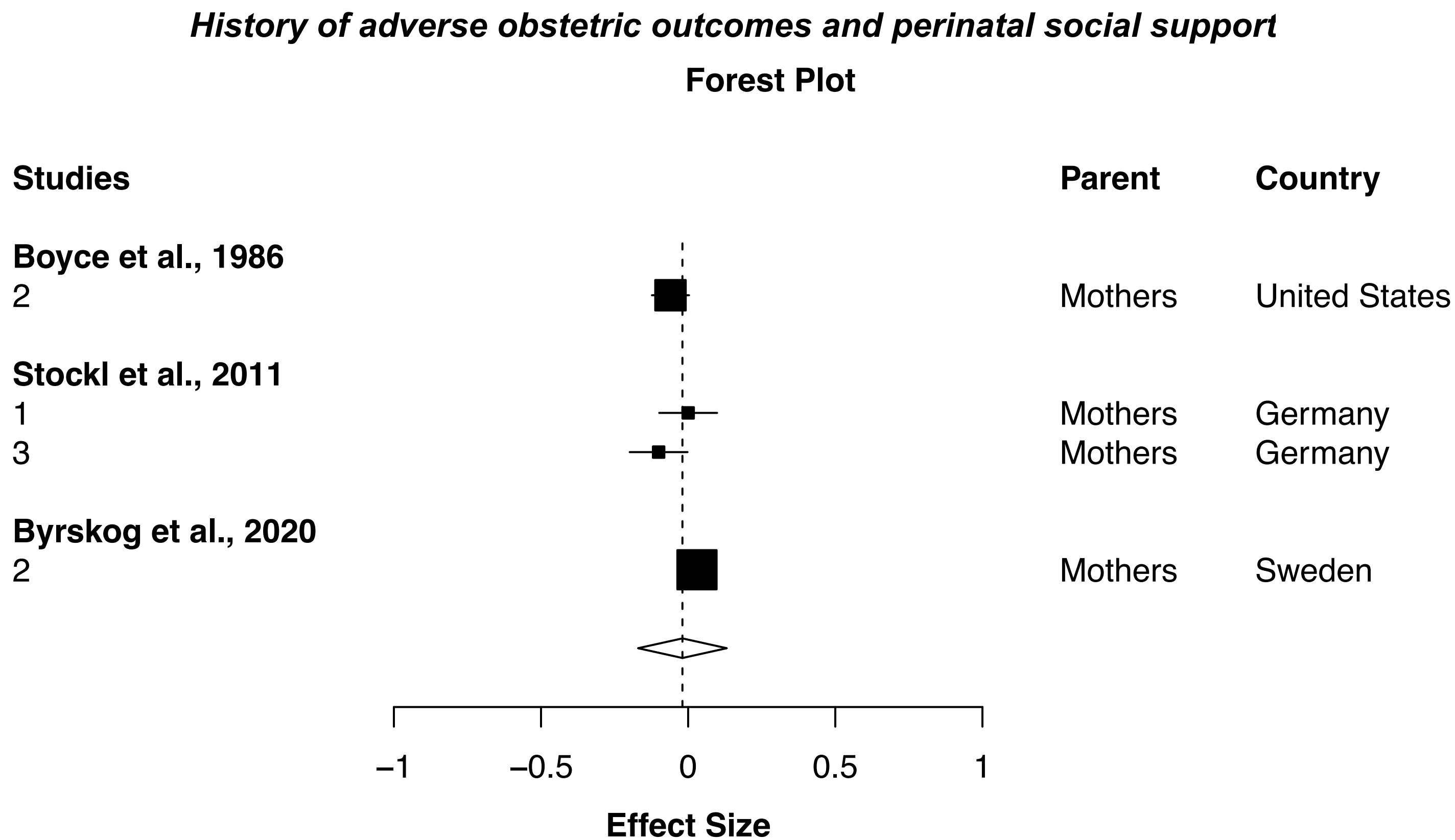
History of adverse obstetric outcomes and perinatal social support
Forest Plot
Studies
Parent
Country
Boyce et al., 1986
2
Mothers
United States
Stockl et al., 2011
1
Mothers
Germany
3
Mothers
Germany
Byrskog et al., 2020
2
Mothers
Sweden
−1
−0.5
0
0.5
1
Effect Size

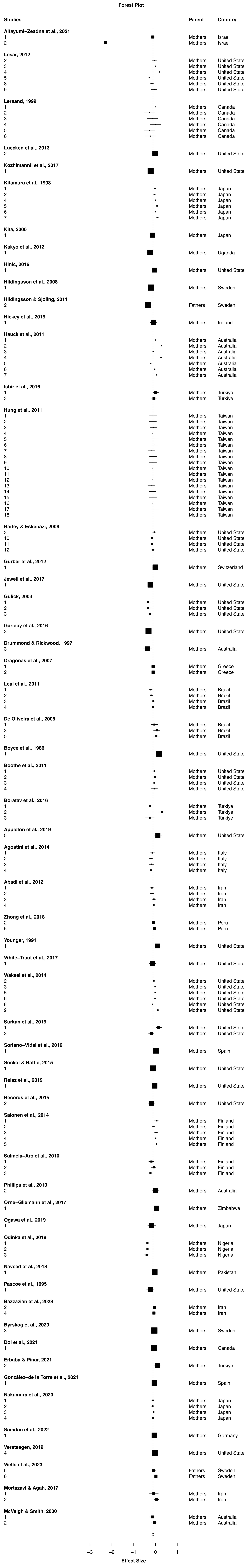
Forest Plot
Studies
Parent
Country
Alfayumi–Zeadna et al., 2021
1 Mothers Israel
2 Mothers Israel
Lesar, 2012
2 Mothers United States
3 Mothers United States
4 Mothers United States
5 Mothers United States
8 Mothers United States
9 Mothers United States
Leraand, 1999
1 Mothers Canada
2 Mothers Canada
3 Mothers Canada
4 Mothers Canada
5 Mothers Canada
6 Mothers Canada
Luecken et al., 2013
2 Mothers United States
Kozhimannil et al., 2017
1 Mothers United States
Kitamura et al., 1998
1 Mothers Japan
2 Mothers Japan
4 Mothers Japan
5 Mothers Japan
6 Mothers Japan
7 Mothers Japan
Kita, 2000
1 Mothers Japan
Kakyo et al., 2012
1 Mothers Uganda
Hinic, 2016
1 Mothers United States
Hildingsson et al., 2008
1 Mothers Sweden
Hildingsson & Sjoling, 2011
2 Fathers Sweden
Hickey et al., 2019
1 Mothers Ireland
Hauck et al., 2011
1 Mothers Australia
2 Mothers Australia
3 Mothers Australia
4 Mothers Australia
5 Mothers Australia
6 Mothers Australia
7 Mothers Australia
Isbir et al., 2016
1 Mothers Türkiye
3 Mothers Türkiye
Hung et al., 2011
1 Mothers Taiwan
2 Mothers Taiwan
3 Mothers Taiwan
4 Mothers Taiwan
5 Mothers Taiwan
6 Mothers Taiwan
7 Mothers Taiwan
8 Mothers Taiwan
9 Mothers Taiwan
10 Mothers Taiwan
11 Mothers Taiwan
12 Mothers Taiwan
13 Mothers Taiwan
14 Mothers Taiwan
15 Mothers Taiwan
16 Mothers Taiwan
17 Mothers Taiwan
18 Mothers Taiwan
Harley & Eskenazi, 2006
3 Mothers United States
10 Mothers United States
11 Mothers United States
12 Mothers United States
Gurber et al., 2012
1 Mothers Switzerland
Jewell et al., 2017
1 Mothers United States
Gulick, 2003
1 Mothers United States
2 Mothers United States
3 Mothers United States
Gariepy et al., 2016
3 Mothers United States
Drummond & Rickwood, 1997
3 Mothers Australia
Dragonas et al., 2007
1 Mothers Greece
2 Mothers Greece
Leal et al., 2011
1 Mothers Brazil
2 Mothers Brazil
3 Mothers Brazil
4 Mothers Brazil
De Oliveira et al., 2006
1 Mothers Brazil
3 Mothers Brazil
5 Mothers Brazil
Boyce et al., 1986
1 Mothers United States
Boothe et al., 2011
1 Mothers United States
2 Mothers United States
3 Mothers United States
4 Mothers United States
Boratav et al., 2016
1 Mothers Türkiye
2 Mothers Türkiye
3 Mothers Türkiye
Appleton et al., 2019
5 Mothers United States
Agostini et al., 2014
1 Mothers Italy
2 Mothers Italy
3 Mothers Italy
4 Mothers Italy
Abadi et al., 2012
1 Mothers Iran
2 Mothers Iran
3 Mothers Iran
4 Mothers Iran
Zhong et al., 2018
2 Mothers Peru
5 Mothers Peru
Younger, 1991
1 Mothers United States
White–Traut et al., 2017
1 Mothers United States
Wakeel et al., 2014
2 Mothers United States
3 Mothers United States
5 Mothers United States
6 Mothers United States
8 Mothers United States
9 Mothers United States
Surkan et al., 2019
1 Mothers United States
3 Mothers United States
Soriano–Vidal et al., 2016
1 Mothers Spain
Sockol & Battle, 2015
1 Mothers United States
Reisz et al., 2019
1 Mothers United States
Records et al., 2015
2 Mothers United States
Salonen et al., 2014
1 Mothers Finland
2 Mothers Finland
3 Mothers Finland
4 Mothers Finland
5 Mothers Finland
Salmela–Aro et al., 2010
1 Mothers Finland
2 Mothers Finland
3 Mothers Finland
Phillips et al., 2010
2 Mothers Australia
Orne–Gliemann et al., 2017
1 Mothers Zimbabwe
Ogawa et al., 2019
1 Mothers Japan
Odinka et al., 2019
1 Mothers Nigeria
2 Mothers Nigeria
3 Mothers Nigeria
Naveed et al., 2018
1 Mothers Pakistan
Pascoe et al., 1995
1 Mothers United States
Bazzazian et al., 2023
2 Mothers Iran
4 Mothers Iran
Byrskog et al., 2020
3 Mothers Sweden
Dol et al., 2021
1 Mothers Canada
Erbaba & Pinar, 2021
2 Mothers Türkiye
González–de la Torre et al., 2021
1 Mothers Spain
Nakamura et al., 2020
1 Mothers Japan
2 Mothers Japan
3 Mothers Japan
4 Mothers Japan
Samdan et al., 2022
1 Mothers Germany
Versteegen, 2019
4 Mothers United States
Wells et al., 2023
5 Fathers Sweden
6 Fathers Sweden
Mortazavi & Agah, 2017
1 Mothers Iran
2 Mothers Iran
McVeigh & Smith, 2000
1 Mothers Australia
2 Mothers Australia
−3
−2
−1
0
1
Effect Size

Previous caesarean section and perinatal social support

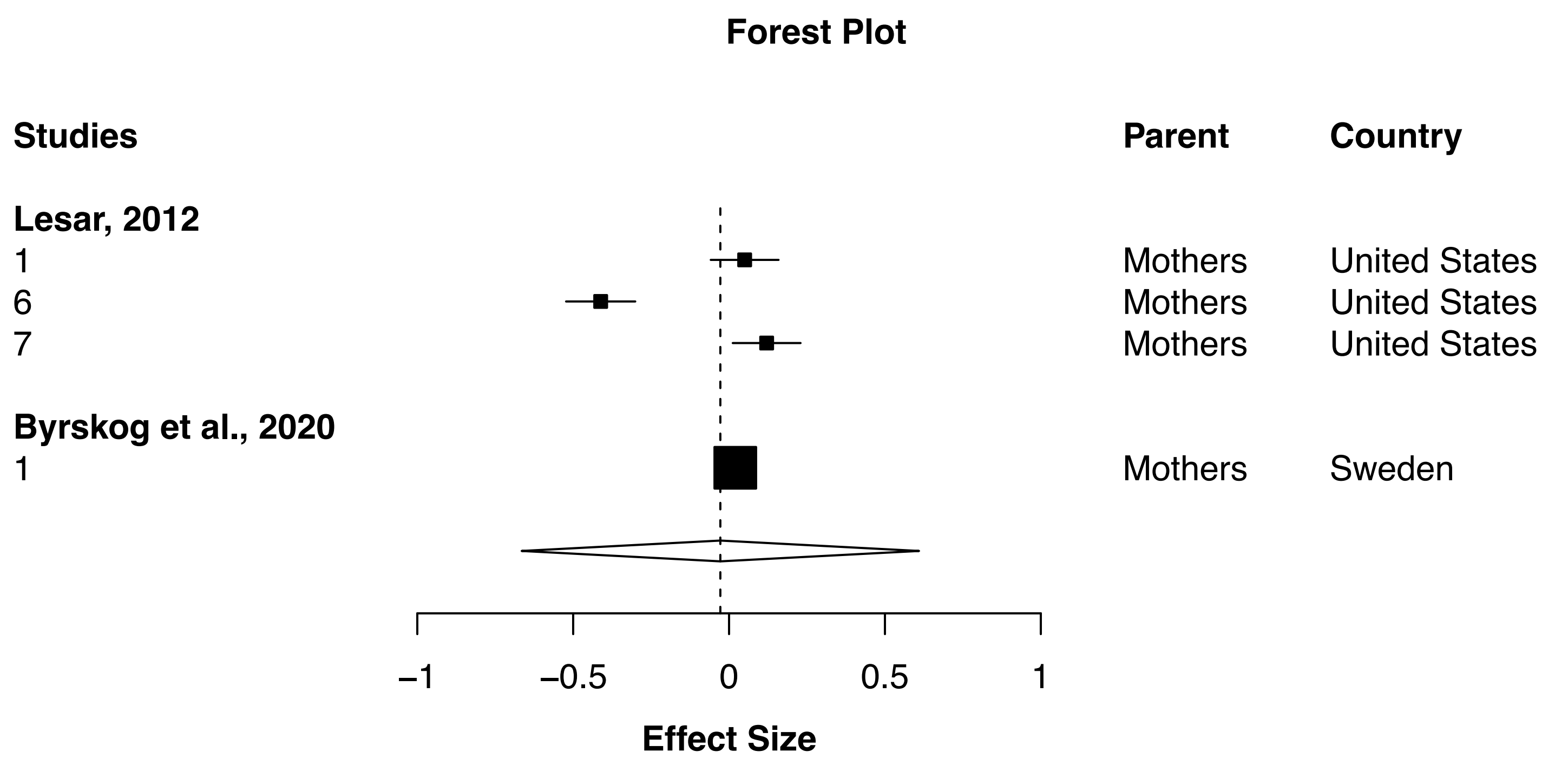

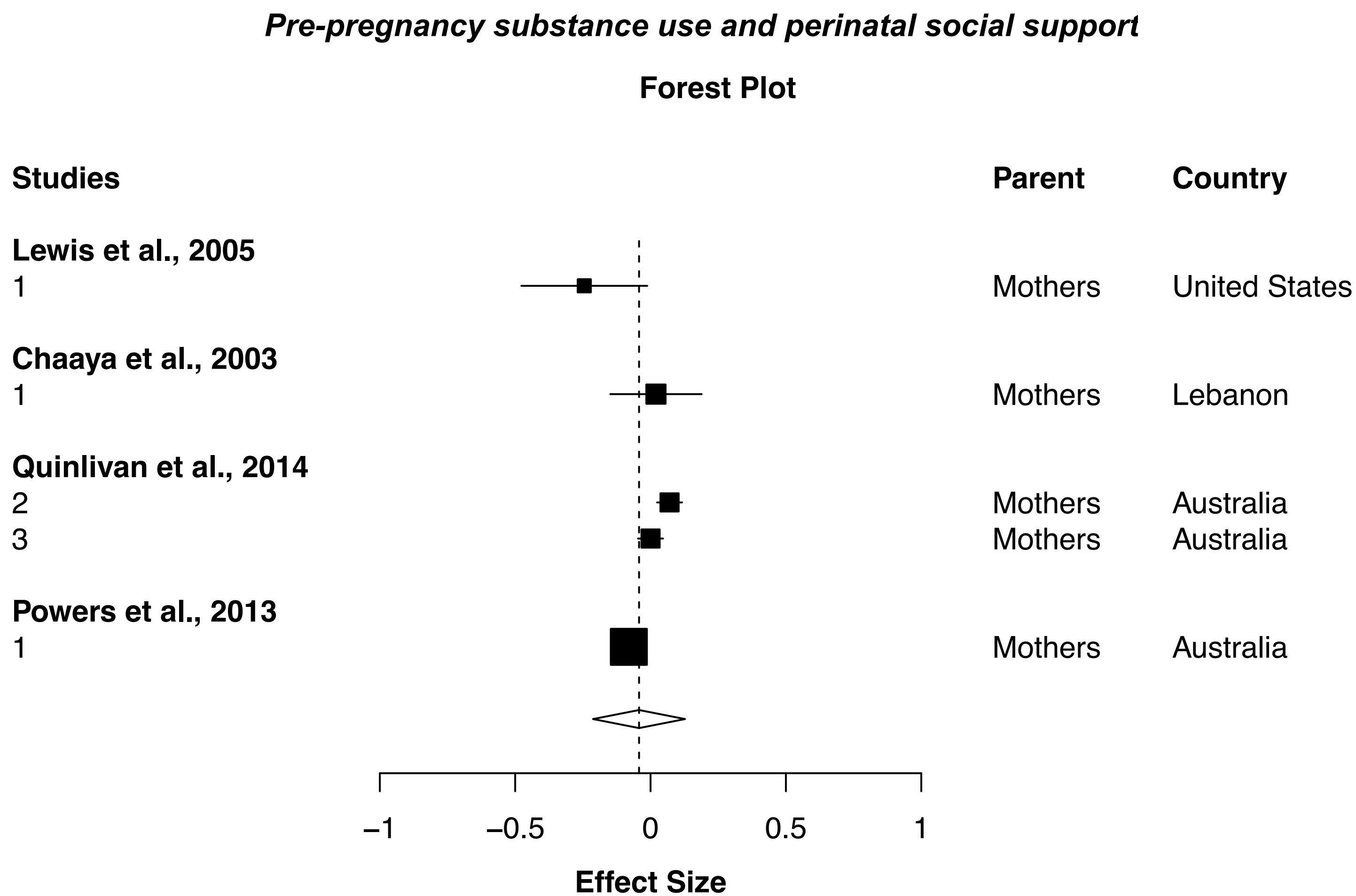

Pre-pregnancy substance use and perinatal social support
Forest Plot
Studies
Parent
Country
Lewis et al., 2005
1
Mothers
United States
Chaaya et al., 2003
1
Mothers
Lebanon
Quinlivan et al., 2014
2
Mothers
Australia
3
Mothers
Australia
Powers et al., 2013
1
Mothers
Australia
−1
−0.5
0
0.5
1
Effect Size

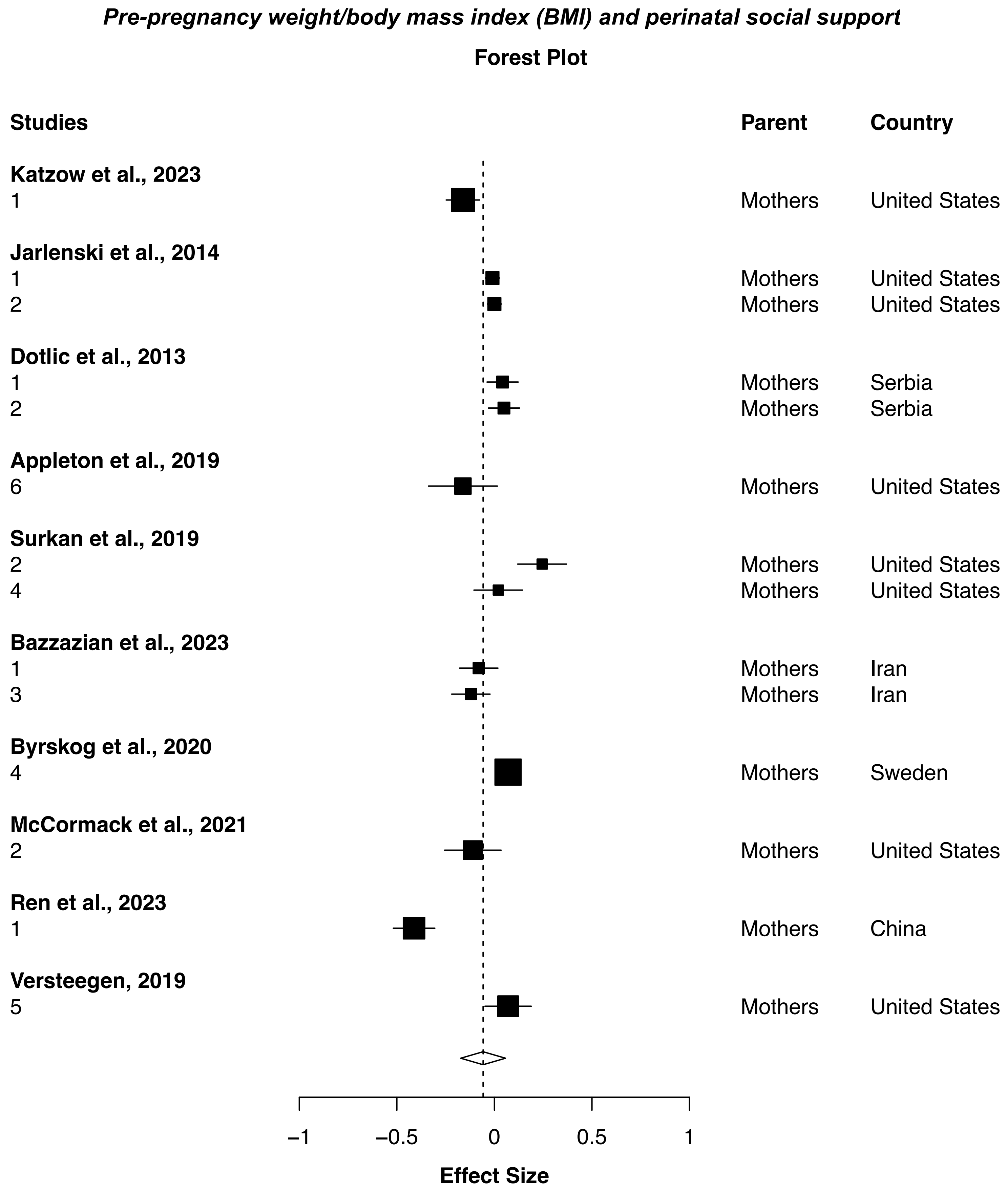
Pre-pregnancy weight/body mass index (BMI) and perinatal social support
Forest Plot
Studies
Parent
Country
Katzow et al., 2023
1
Mothers
United States
Jarlenski et al., 2014
1
Mothers
United States
2
Mothers
United States
Dotlic et al., 2013
1
Mothers
Serbia
2
Mothers
Serbia
Appleton et al., 2019
6
Mothers
United States
Surkan et al., 2019
2
Mothers
United States
4
Mothers
United States
Bazzazian et al., 2023
1
Mothers
Iran
3
Mothers
Iran
Byrskog et al., 2020
4
Mothers
Sweden
McCormack et al., 2021
2
Mothers
United States
Ren et al., 2023
1
Mothers
China
Versteegen, 2019
5
Mothers
United States
–1
–0.5
0
0.5
1
Effect Size

Pregnancy intention (unplanned) and perinatal social support

Forest Plot

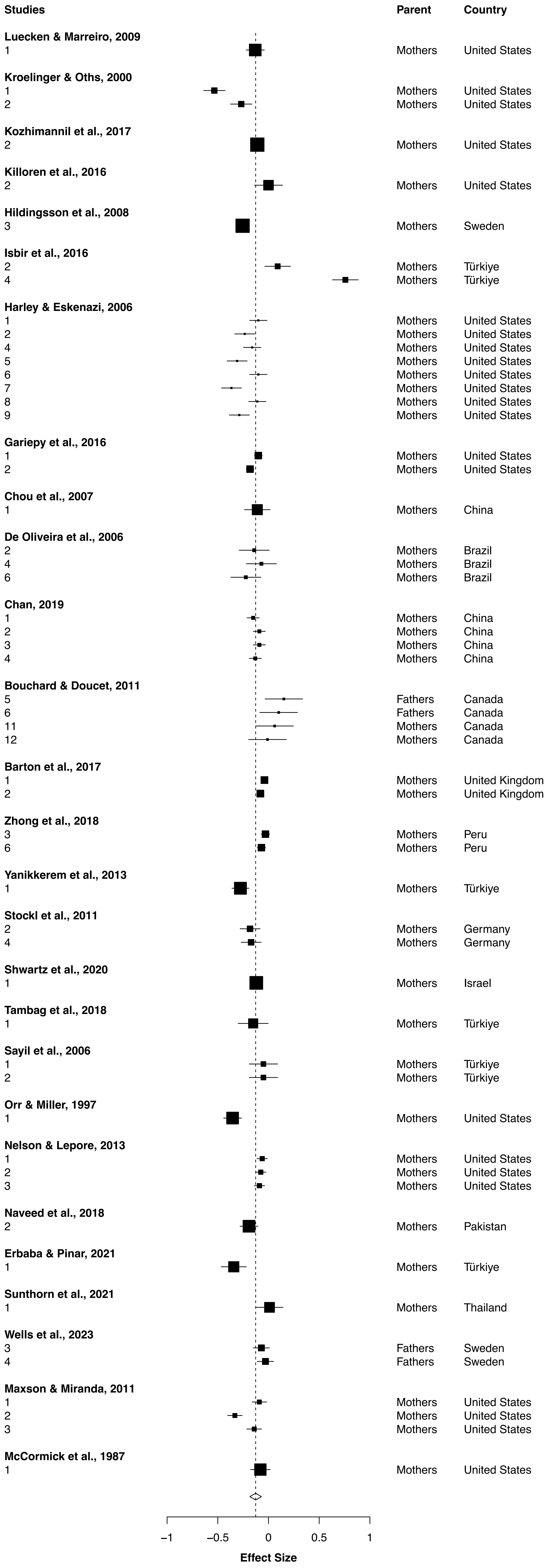

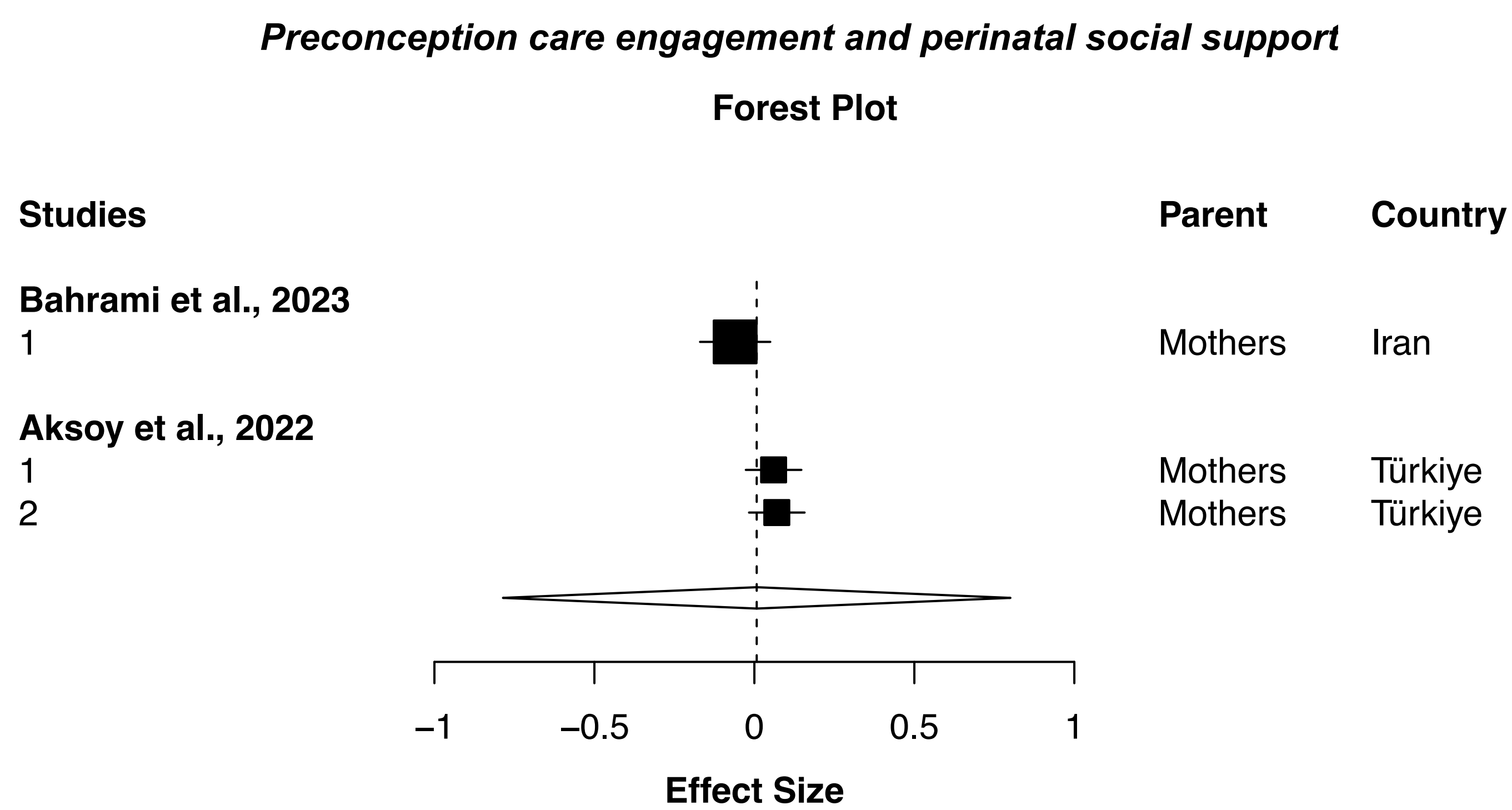

Preconception care engagement and perinatal social support
Forest Plot
Studies
Parent
Country
Bahrami et al., 2023
1
Mothers
Iran
Aksoy et al., 2022
1
Mothers
Türkiye
2
Mothers
Türkiye
−1
−0.5
0
0.5
1
Effect Size

Method of conception (assisted reproduction) and perinatal social support

Forest Plot

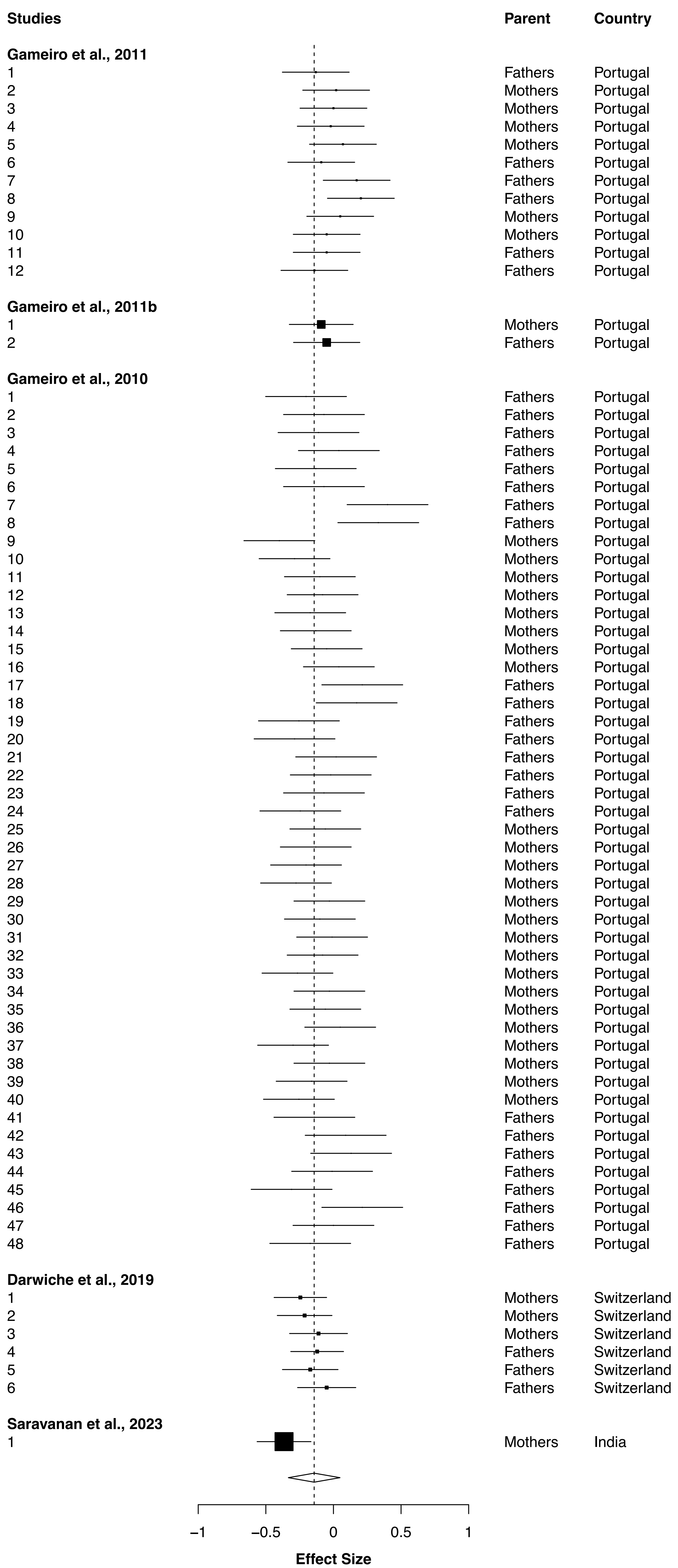

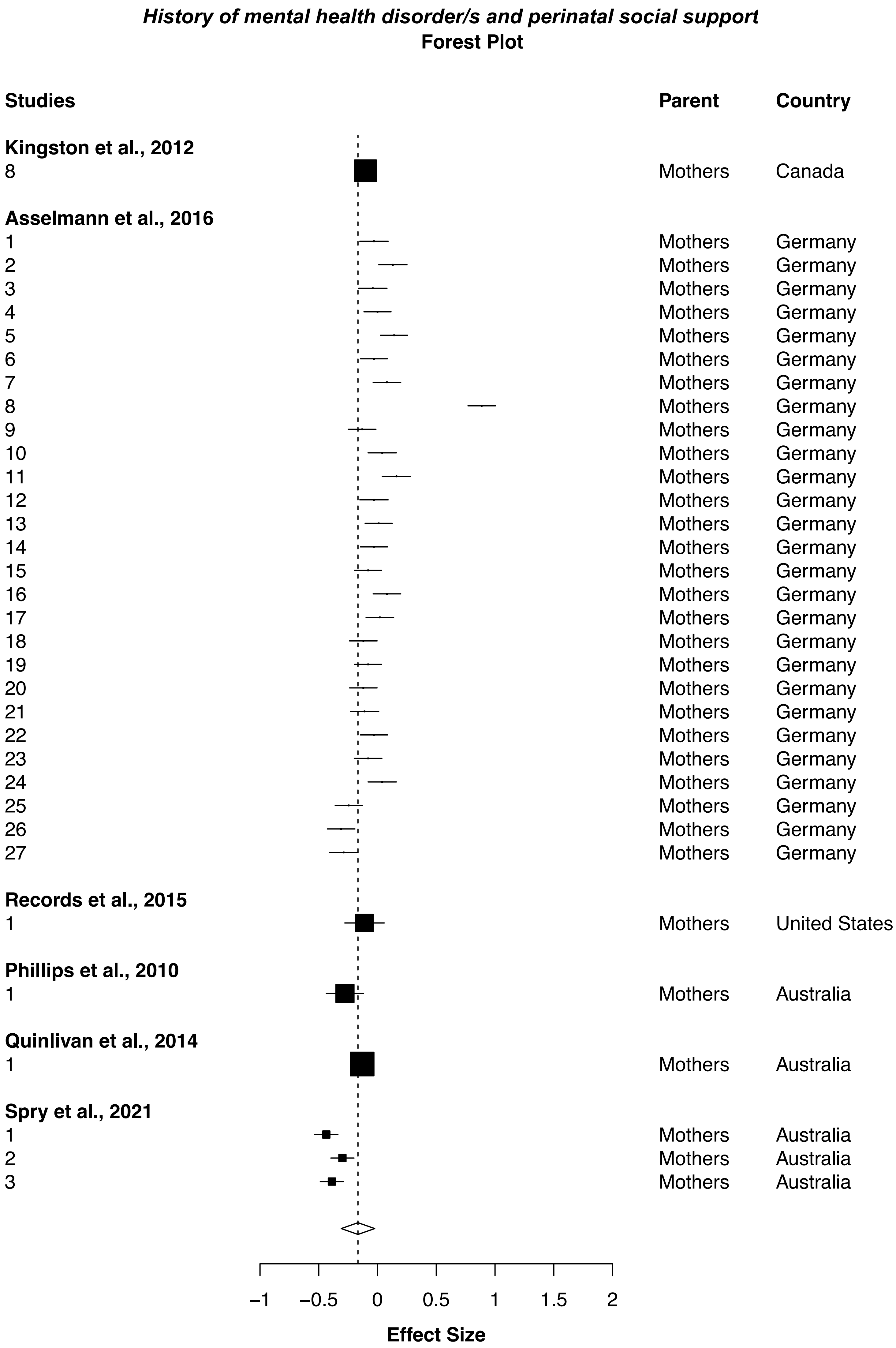
History of mental health disorder/s and perinatal social support
Forest Plot
Studies
Parent
Country
Kingston et al., 2012
8 Mothers Canada
Asselmann et al., 2016
1 Mothers Germany
2 Mothers Germany
3 Mothers Germany
4 Mothers Germany
5 Mothers Germany
6 Mothers Germany
7 Mothers Germany
8 Mothers Germany
9 Mothers Germany
10 Mothers Germany
11 Mothers Germany
12 Mothers Germany
13 Mothers Germany
14 Mothers Germany
15 Mothers Germany
16 Mothers Germany
17 Mothers Germany
18 Mothers Germany
19 Mothers Germany
20 Mothers Germany
21 Mothers Germany
22 Mothers Germany
23 Mothers Germany
24 Mothers Germany
25 Mothers Germany
26 Mothers Germany
27 Mothers Germany
Records et al., 2015
1 Mothers United States
Phillips et al., 2010
1 Mothers Australia
Quinlivan et al., 2014
1 Mothers Australia
Spry et al., 2021
1 Mothers Australia
2 Mothers Australia
3 Mothers Australia
−1
−0.5
0
0.5
1
1.5
2
Effect Size

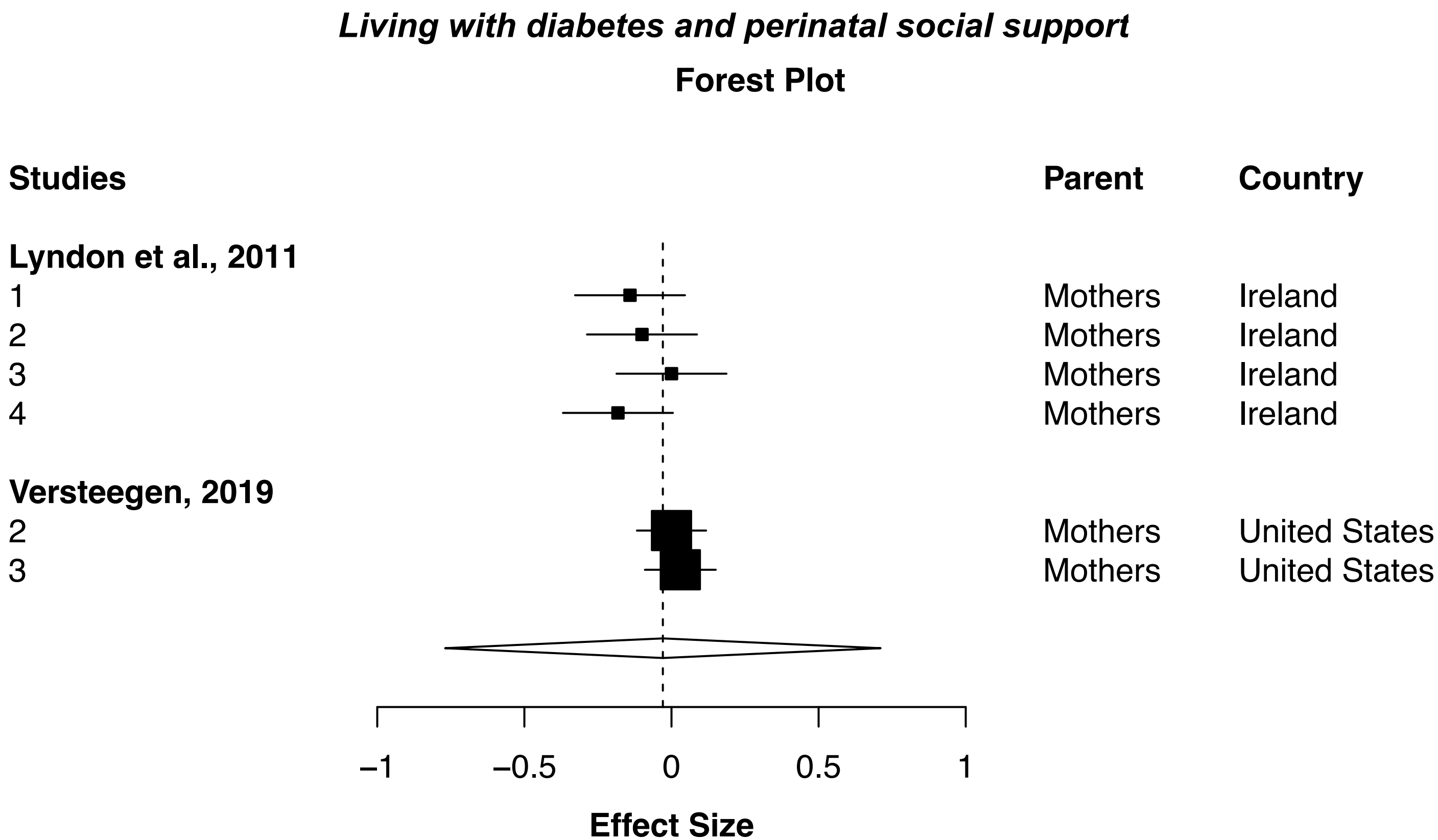

Living with diabetes and perinatal social support
Forest Plot
Studies
Parent
Country
Lyndon et al., 2011
1
2
3
4
Mothers
Mothers
Mothers
Mothers
Ireland
Ireland
Ireland
Ireland
Versteegen, 2019
2
3
Mothers
Mothers
United States
United States
−1
−0.5
0
0.5
1
Effect Size

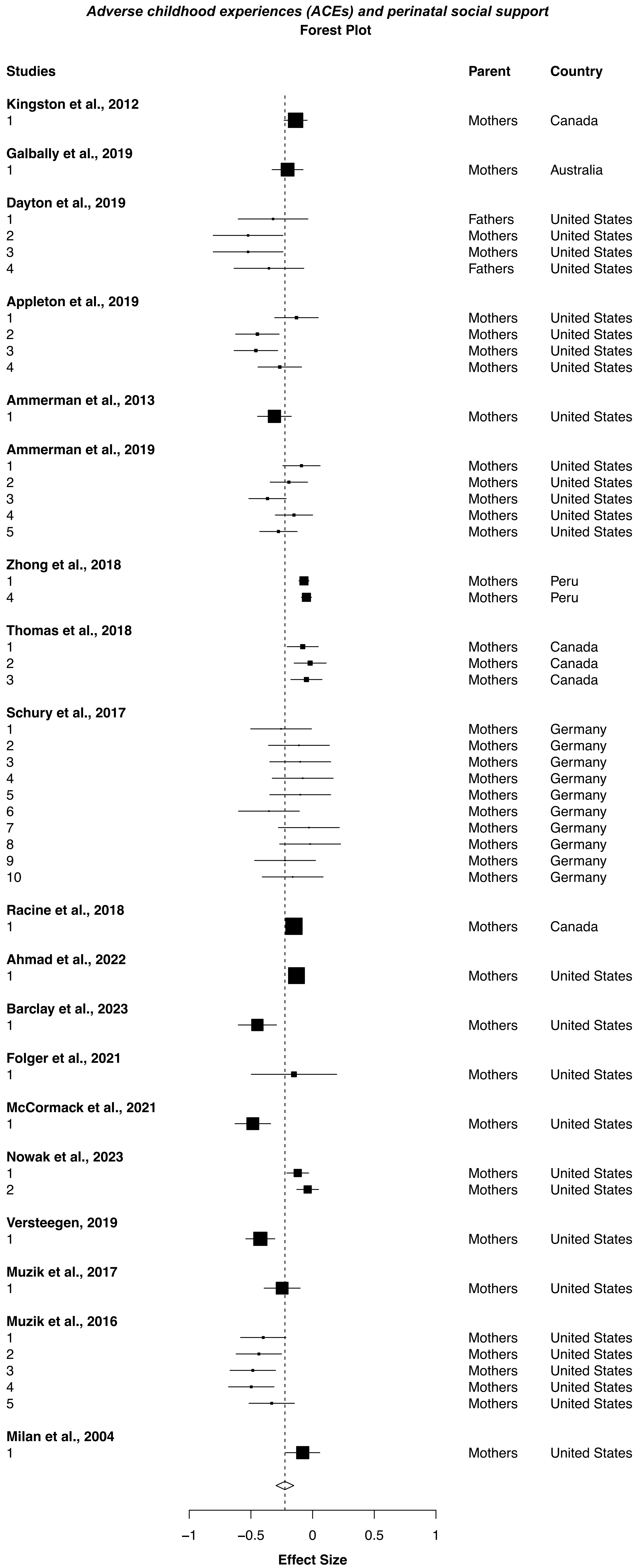
Adverse childhood experiences (ACEs) and perinatal social support
Forest Plot
Studies
Parent
Country
Kingston et al., 2012
1
Mothers
Canada
Galbally et al., 2019
1
Mothers
Australia
Dayton et al., 2019
1
Fathers
United States
2
Mothers
United States
3
Mothers
United States
4
Fathers
United States
Appleton et al., 2019
1
Mothers
United States
2
Mothers
United States
3
Mothers
United States
4
Mothers
United States
Ammerman et al., 2013
1
Mothers
United States
Ammerman et al., 2019
1
Mothers
United States
2
Mothers
United States
3
Mothers
United States
4
Mothers
United States
5
Mothers
United States
Zhong et al., 2018
1
Mothers
Peru
4
Mothers
Peru
Thomas et al., 2018
1
Mothers
Canada
2
Mothers
Canada
3
Mothers
Canada
Schury et al., 2017
1
Mothers
Germany
2
Mothers
Germany
3
Mothers
Germany
4
Mothers
Germany
5
Mothers
Germany
6
Mothers
Germany
7
Mothers
Germany
8
Mothers
Germany
9
Mothers
Germany
10
Mothers
Germany
Racine et al., 2018
1
Mothers
Canada
Ahmad et al., 2022
1
Mothers
United States
Barclay et al., 2023
1
Mothers
United States
Folger et al., 2021
1
Mothers
United States
McCormack et al., 2021
1
Mothers
United States
Nowak et al., 2023
1
Mothers
United States
2
Mothers
United States
Versteegen, 2019
1
Mothers
United States
Muzik et al., 2017
1
Mothers
United States
Muzik et al., 2016
1
Mothers
United States
2
Mothers
United States
3
Mothers
United States
4
Mothers
United States
5
Mothers
United States
Milan et al., 2004
1
Mothers
United States
−1
−0.5
0
0.5
1
Effect Size

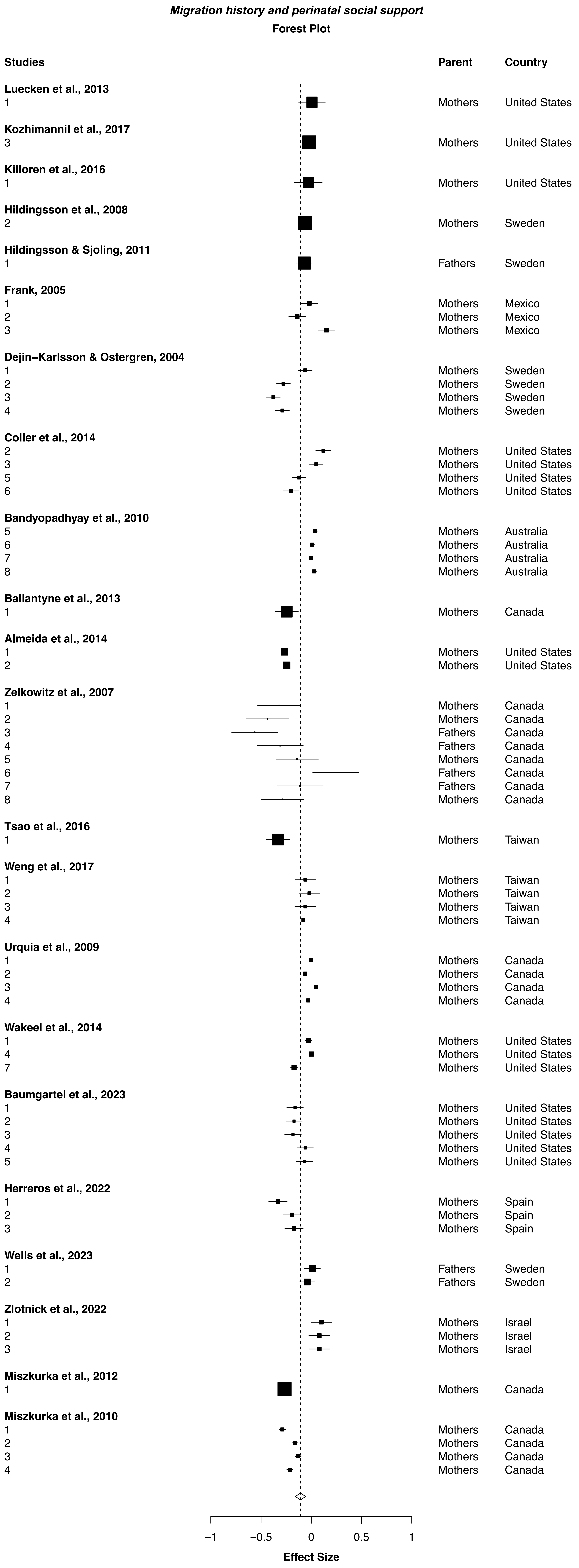

Migration history and perinatal social support
Forest Plot
Studies
Parent
Country
Luecken et al., 2013
1 Mothers United States
Kozhimannil et al., 2017
3 Mothers United States
Killoren et al., 2016
1 Mothers United States
Hildingsson et al., 2008
2 Mothers Sweden
Hildingsson & Sjoling, 2011
1 Fathers Sweden
Frank, 2005
1 Mothers Mexico
2 Mothers Mexico
3 Mothers Mexico
Dejin–Karlsson & Ostergren, 2004
1 Mothers Sweden
2 Mothers Sweden
3 Mothers Sweden
4 Mothers Sweden
Coller et al., 2014
2 Mothers United States
3 Mothers United States
5 Mothers United States
6 Mothers United States
Bandyopadhyay et al., 2010
5 Mothers Australia
6 Mothers Australia
7 Mothers Australia
8 Mothers Australia
Ballantyne et al., 2013
1 Mothers Canada
Almeida et al., 2014
1 Mothers United States
2 Mothers United States
Zelkowitz et al., 2007
1 Mothers Canada
2 Mothers Canada
3 Fathers Canada
4 Fathers Canada
5 Mothers Canada
6 Fathers Canada
7 Fathers Canada
8 Mothers Canada
Tsao et al., 2016
1 Mothers Taiwan
Weng et al., 2017
1 Mothers Taiwan
2 Mothers Taiwan
3 Mothers Taiwan
4 Mothers Taiwan
Urquia et al., 2009
1 Mothers Canada
2 Mothers Canada
3 Mothers Canada
4 Mothers Canada
Wakeel et al., 2014
1 Mothers United States
4 Mothers United States
7 Mothers United States
Baumgartel et al., 2023
1 Mothers United States
2 Mothers United States
3 Mothers United States
4 Mothers United States
5 Mothers United States
Herreros et al., 2022
1 Mothers Spain
2 Mothers Spain
3 Mothers Spain
Wells et al., 2023
1 Fathers Sweden
2 Fathers Sweden
Zlotnick et al., 2022
1 Mothers Israel
2 Mothers Israel
3 Mothers Israel
Miszkurka et al., 2012
1 Mothers Canada
Miszkurka et al., 2010
1 Mothers Canada
2 Mothers Canada
3 Mothers Canada
4 Mothers Canada
−1
−0.5
0
0.5
1
Effect Size

Funnel plots

i. History of adverse obstetric outcomes and perinatal social support
ii. Parity and perinatal social support
iii. Previous caesarean section and perinatal social support
iv. Pre-pregnancy substance use and perinatal social support
v. Pre-pregnancy weight/body mass index (BMI) and perinatal social support
vi. Pregnancy intention (unplanned) and perinatal social support
vii. Preconception care engagement and perinatal social support
viii. Method of conception (assisted reproduction) and perinatal social support
ix. History of mental health disorder/s and perinatal social support
x. Living with diabetes and perinatal social support
xi. Adverse childhood experiences (ACEs) and perinatal social support
xii. Migration history and perinatal social support

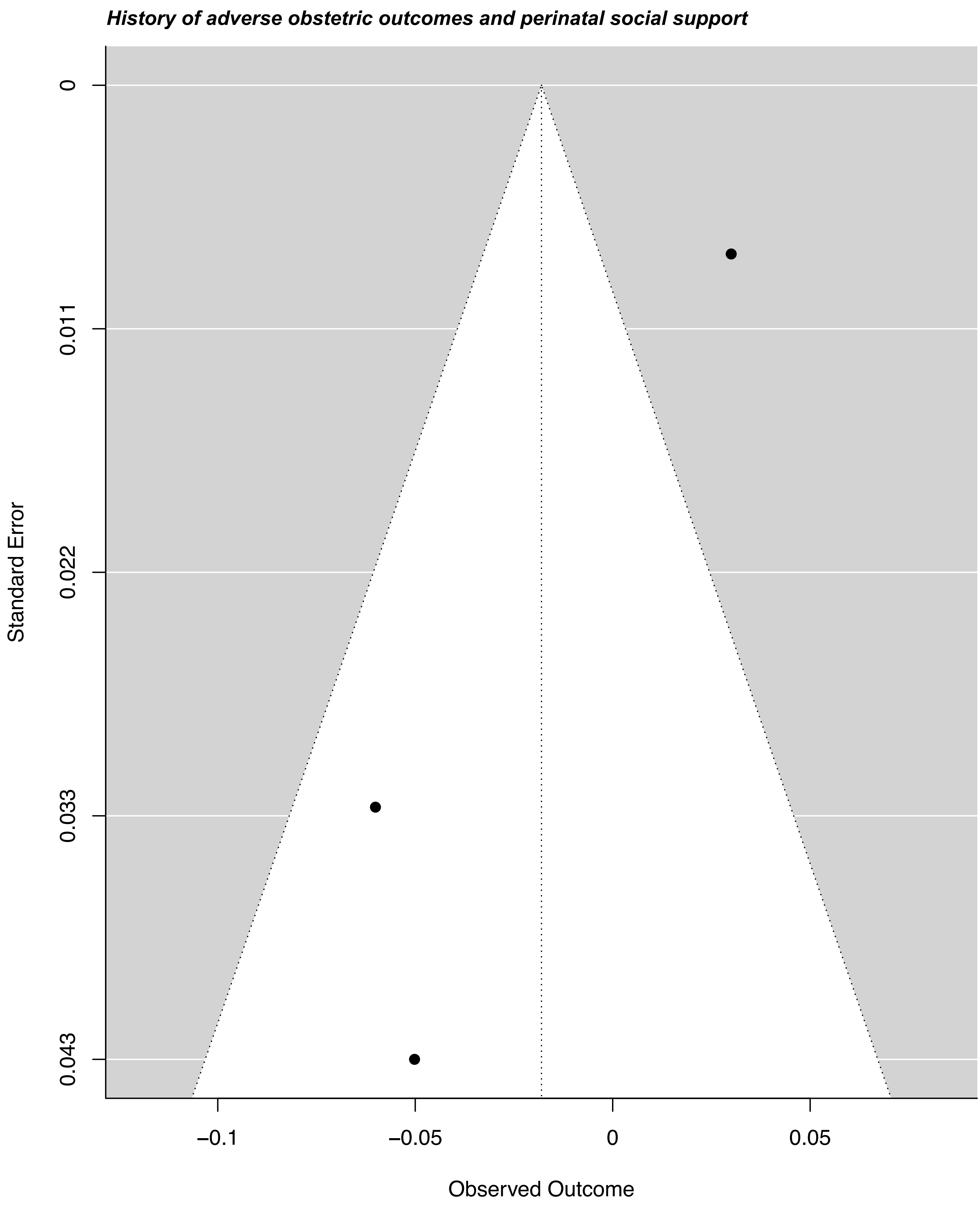
History of adverse obstetric outcomes and perinatal social support
Standard Error
0
0.011
0.022
0.033
0.043
−0.1
−0.05
0
0.05
Observed Outcome

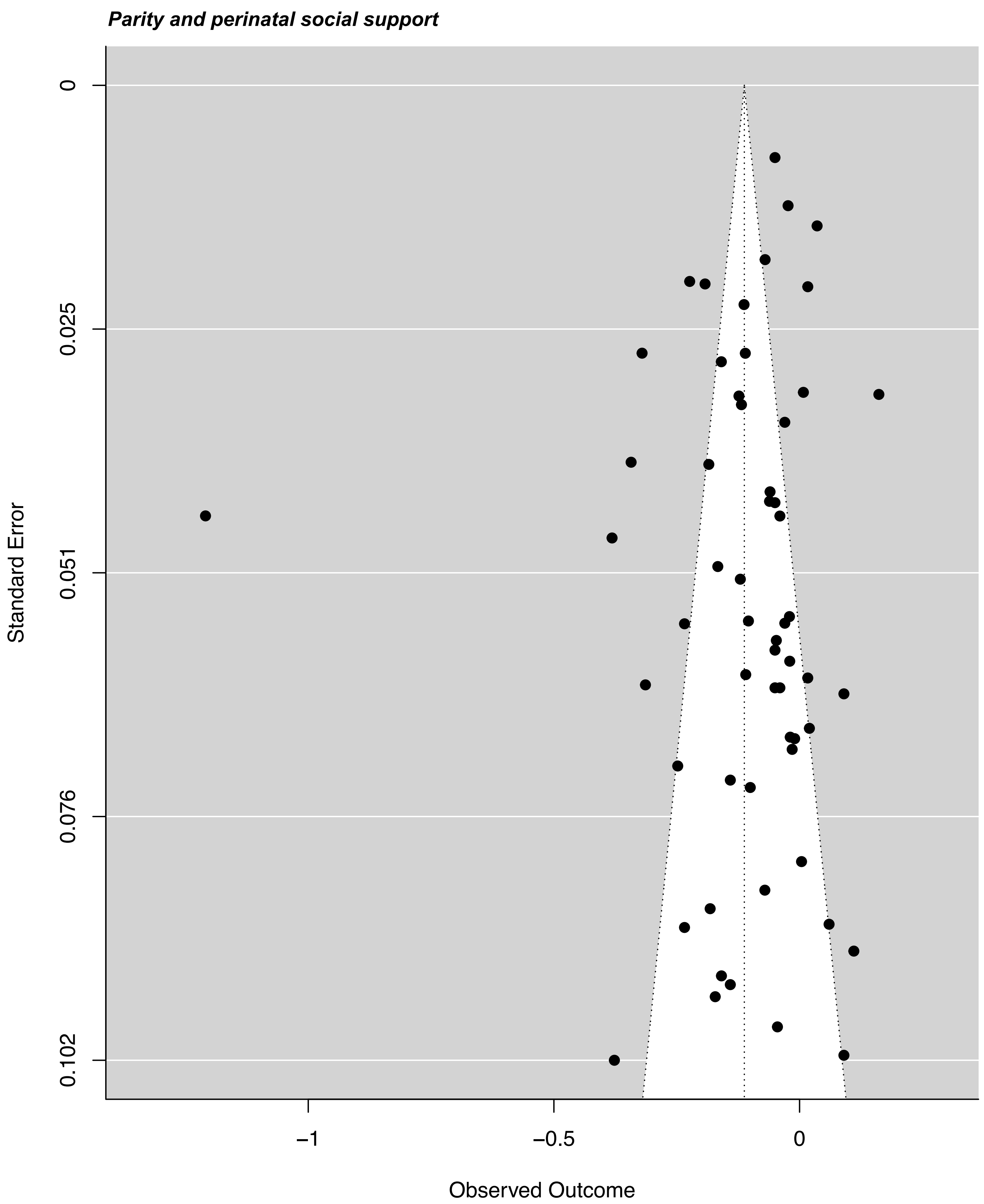

Parity and perinatal social support
Standard Error
0
0.025
0.051
0.076
0.102
−1
−0.5
0
Observed Outcome

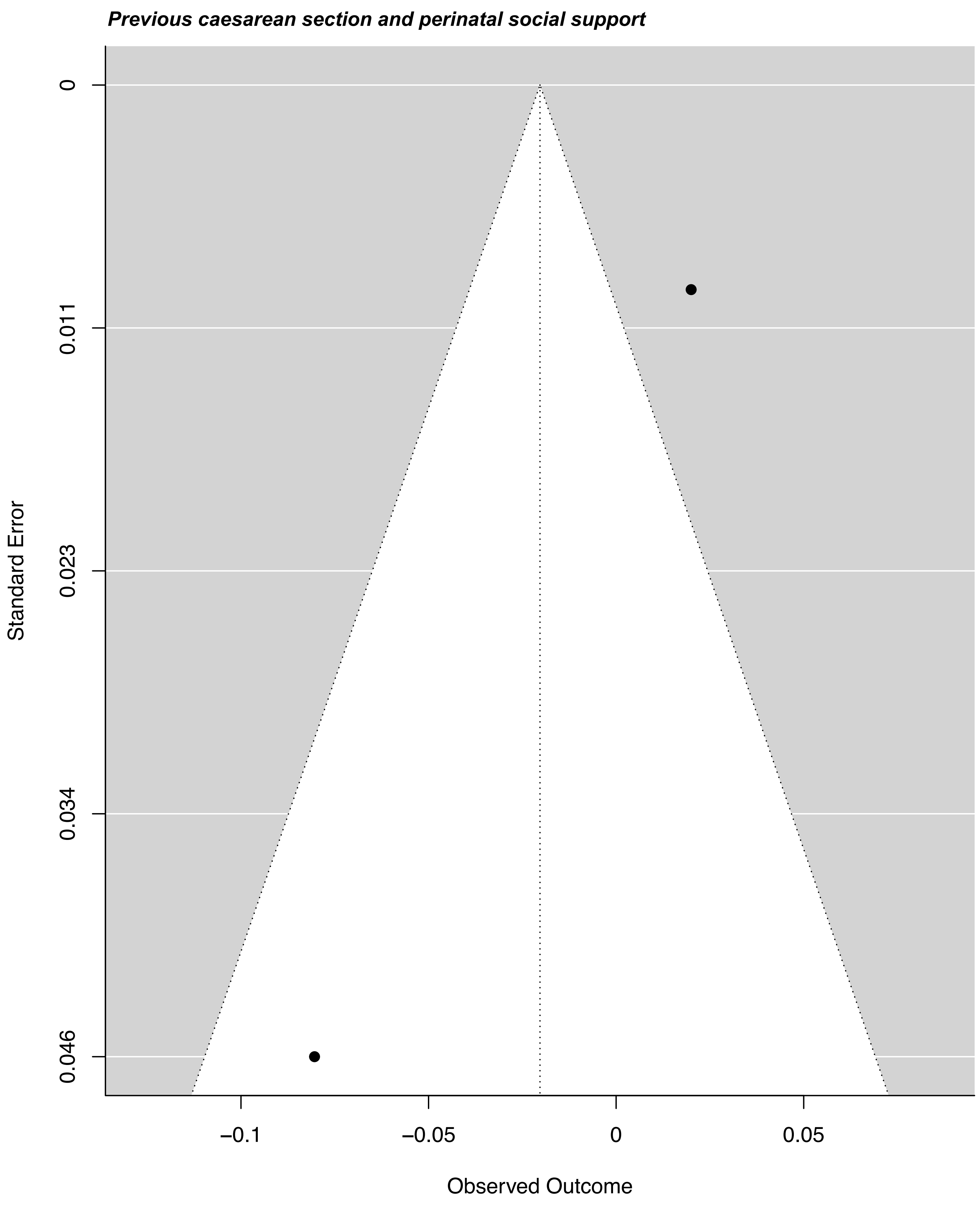
Previous caesarean section and perinatal social support
Standard Error
0
0.011
0.023
0.034
0.046
−0.1
−0.05
0
0.05
Observed Outcome

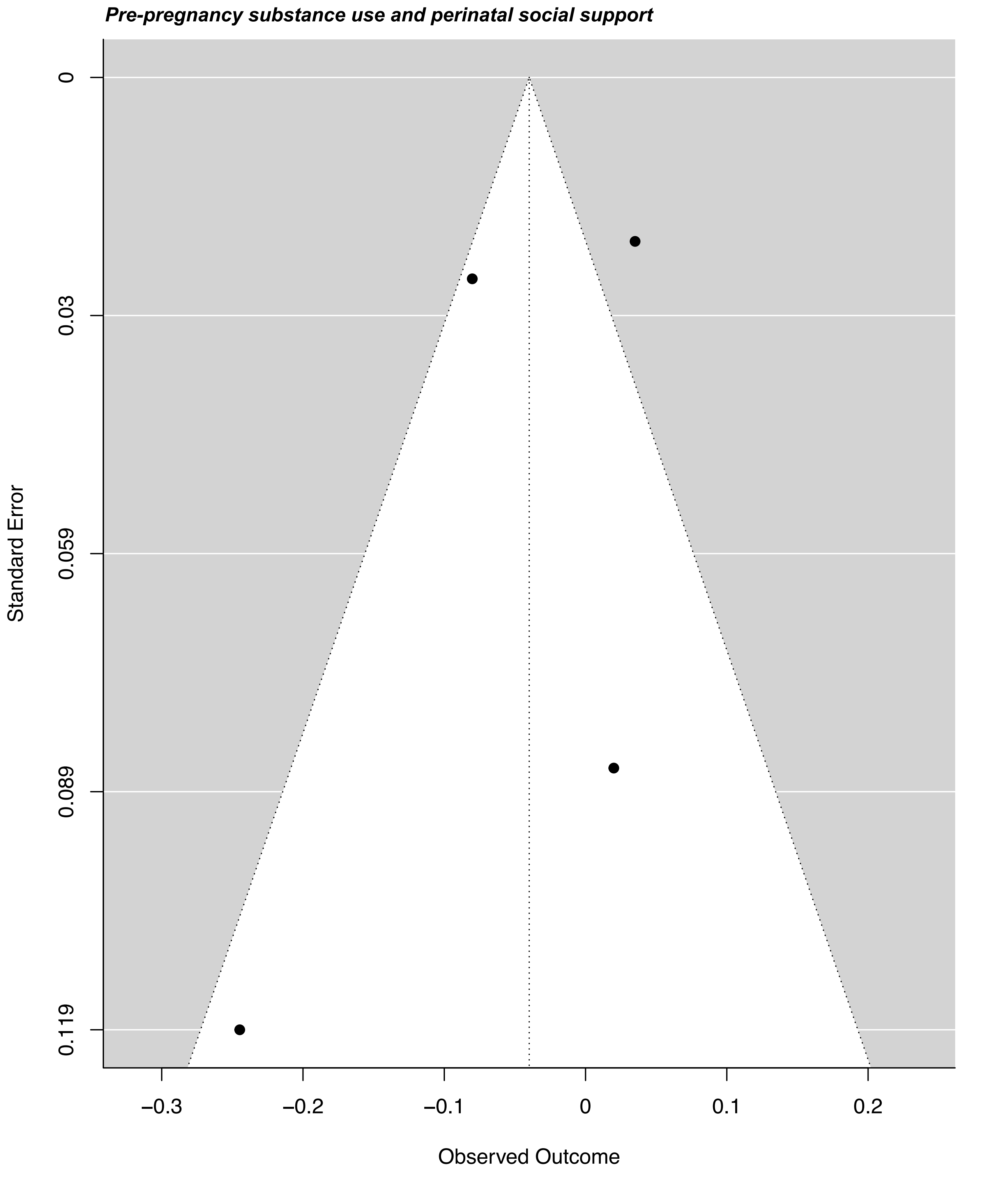
Pre-pregnancy substance use and perinatal social support
Standard Error
0
0.03
0.059
0.089
0.119
−0.3
−0.2
−0.1
0
0.1
0.2
Observed Outcome

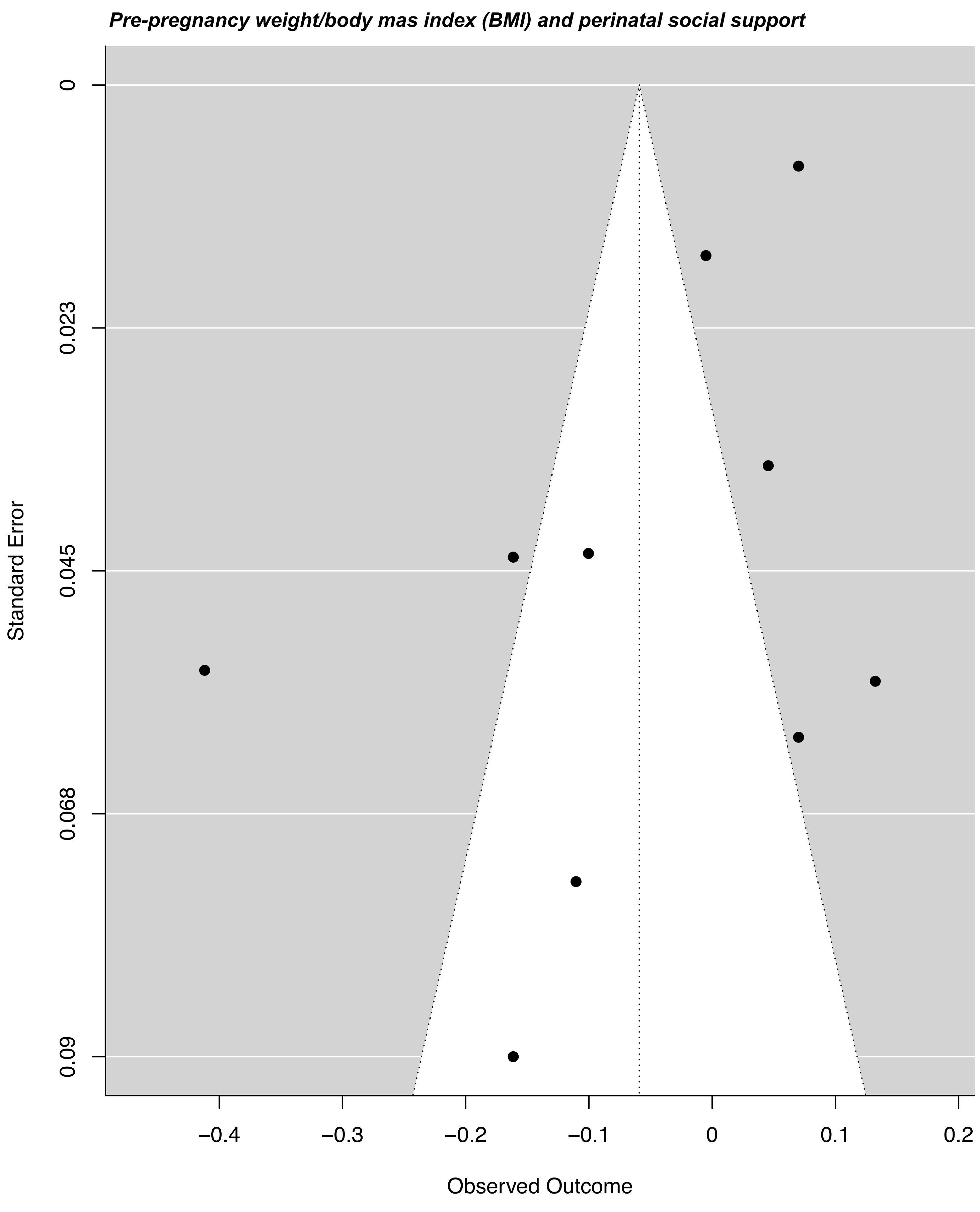
Pre-pregnancy weight/body mas index (BMI) and perinatal social support
Standard Error
0
0.023
0.045
0.068
0.09
Observed Outcome
−0.4
−0.3
−0.2
−0.1
0
0.1
0.2

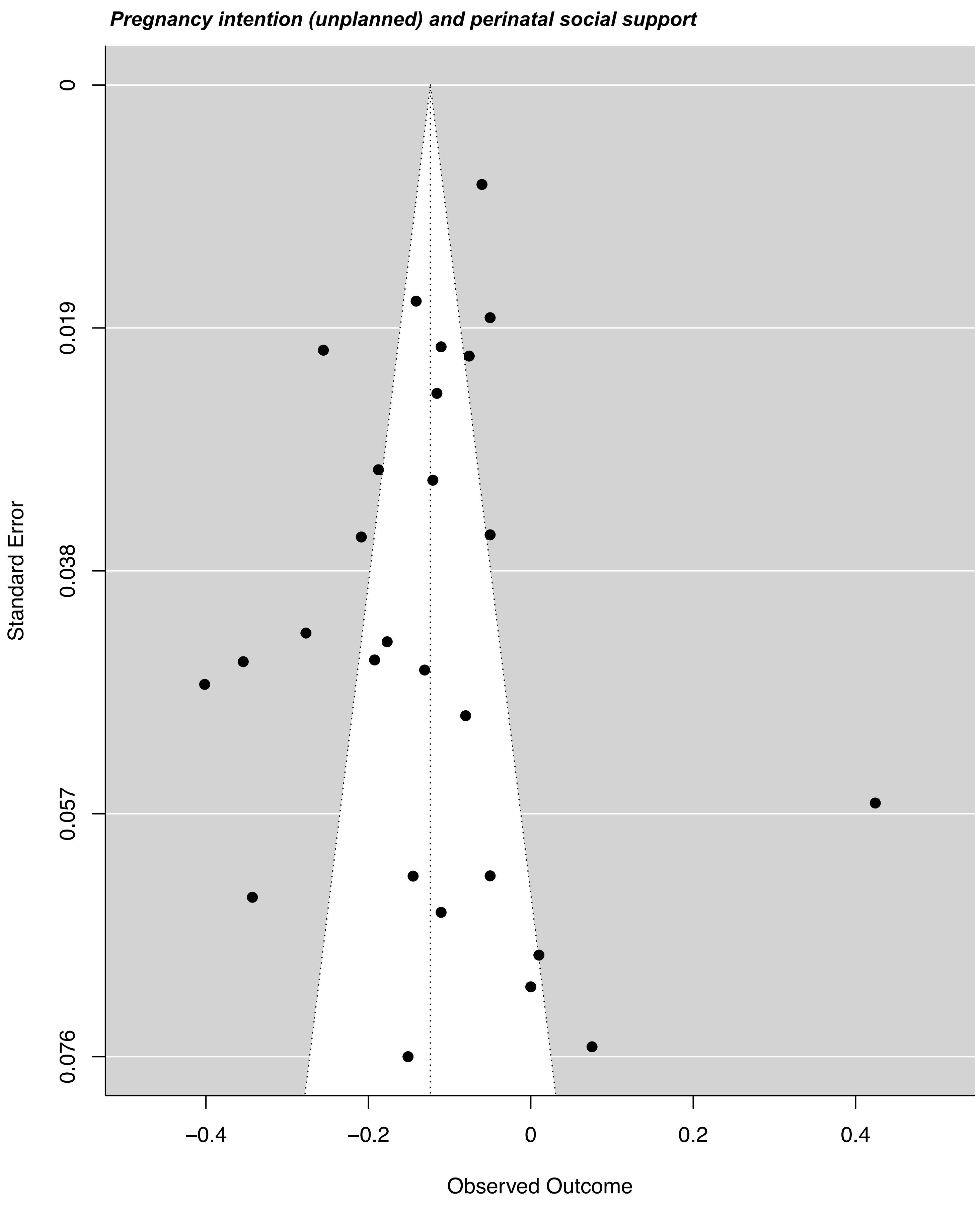

Pregnancy intention (unplanned) and perinatal social support
Standard Error
0
0.019
0.038
0.057
0.076
−0.4
−0.2
0
0.2
0.4
Observed Outcome

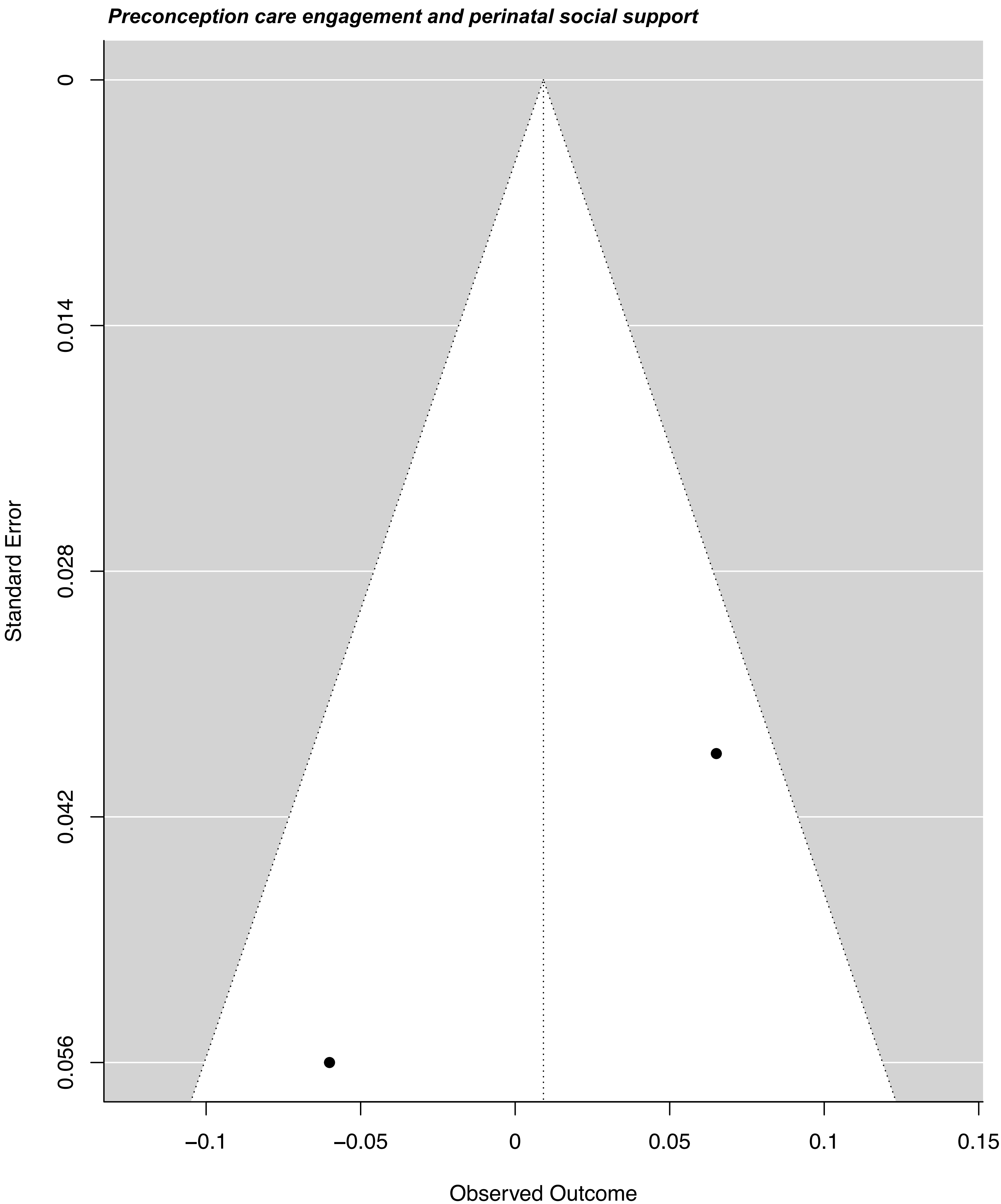

Preconception care engagement and perinatal social support
Standard Error
0
0.014
0.028
0.042
0.056
−0.1
−0.05
0
0.05
0.1
0.15
Observed Outcome

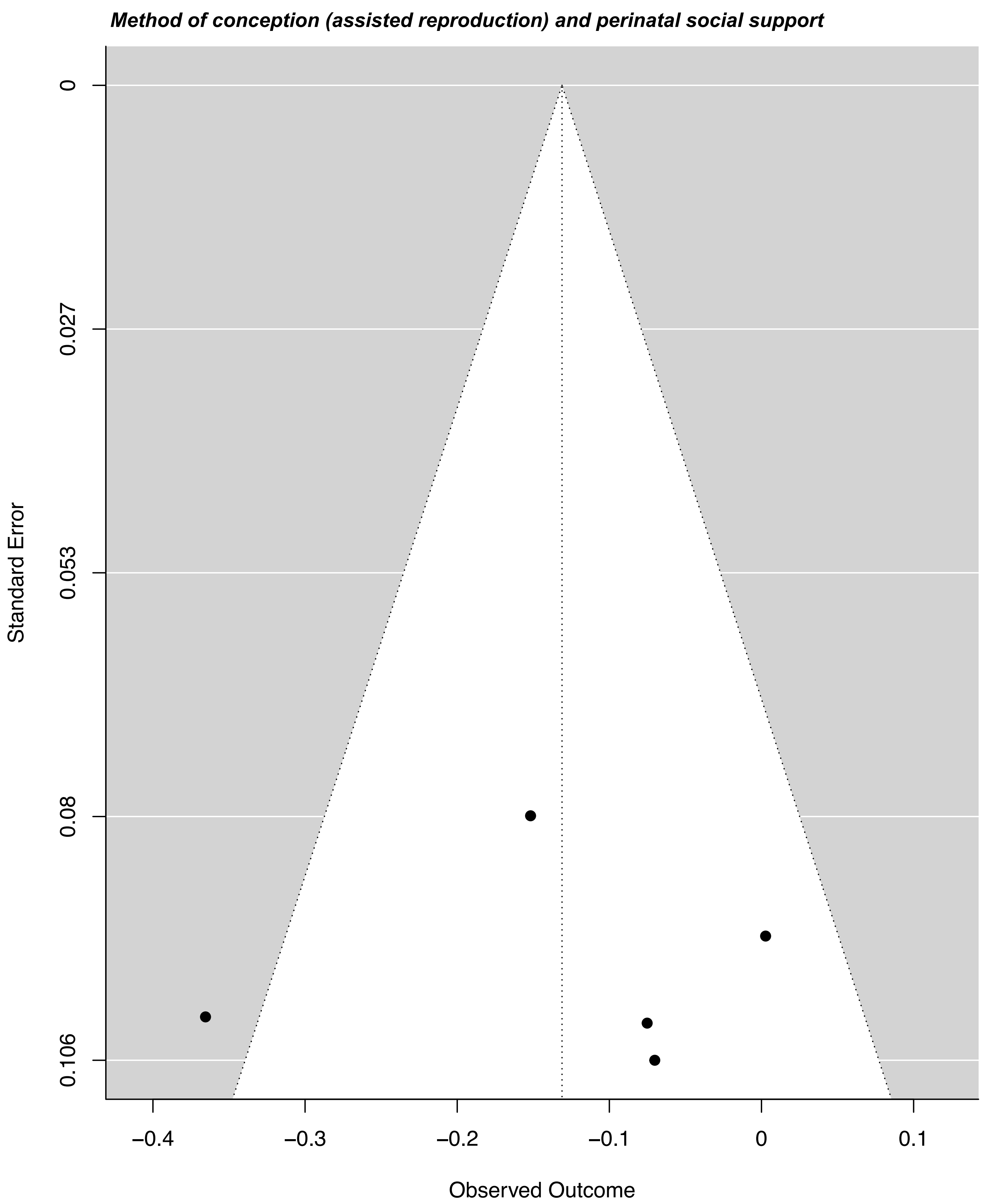

Method of conception (assisted reproduction) and perinatal social support
Standard Error
0
0.027
0.053
0.08
0.106
−0.4
−0.3
−0.2
−0.1
0
0.1
Observed Outcome

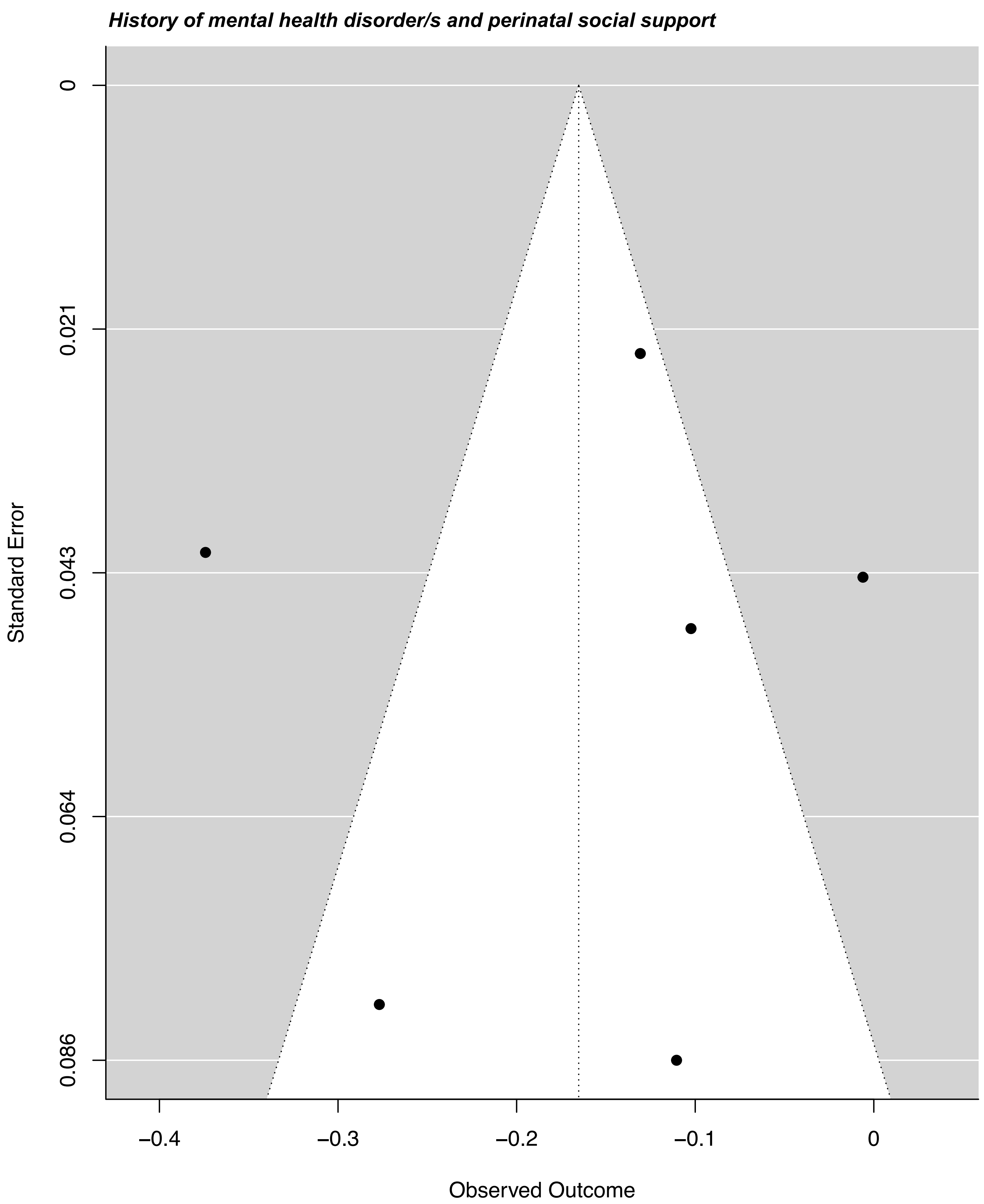

History of mental health disorder/s and perinatal social support
Standard Error
0
0.021
0.043
0.064
0.086
−0.4
−0.3
−0.2
−0.1
0
Observed Outcome

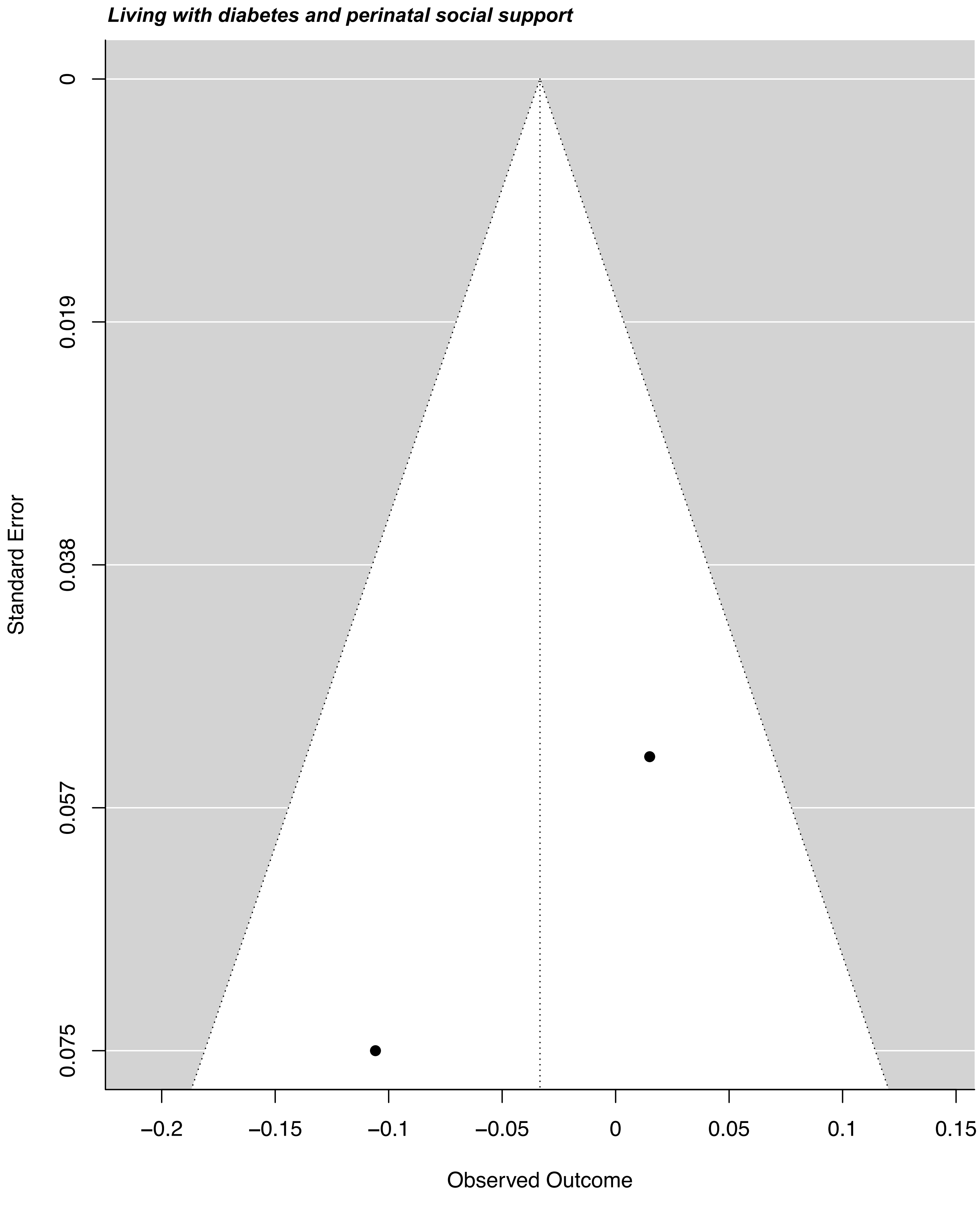

Living with diabetes and perinatal social support
Standard Error
0
0.019
0.038
0.057
0.075
−0.2
−0.15
−0.1
−0.05
0
0.05
0.1
0.15
Observed Outcome

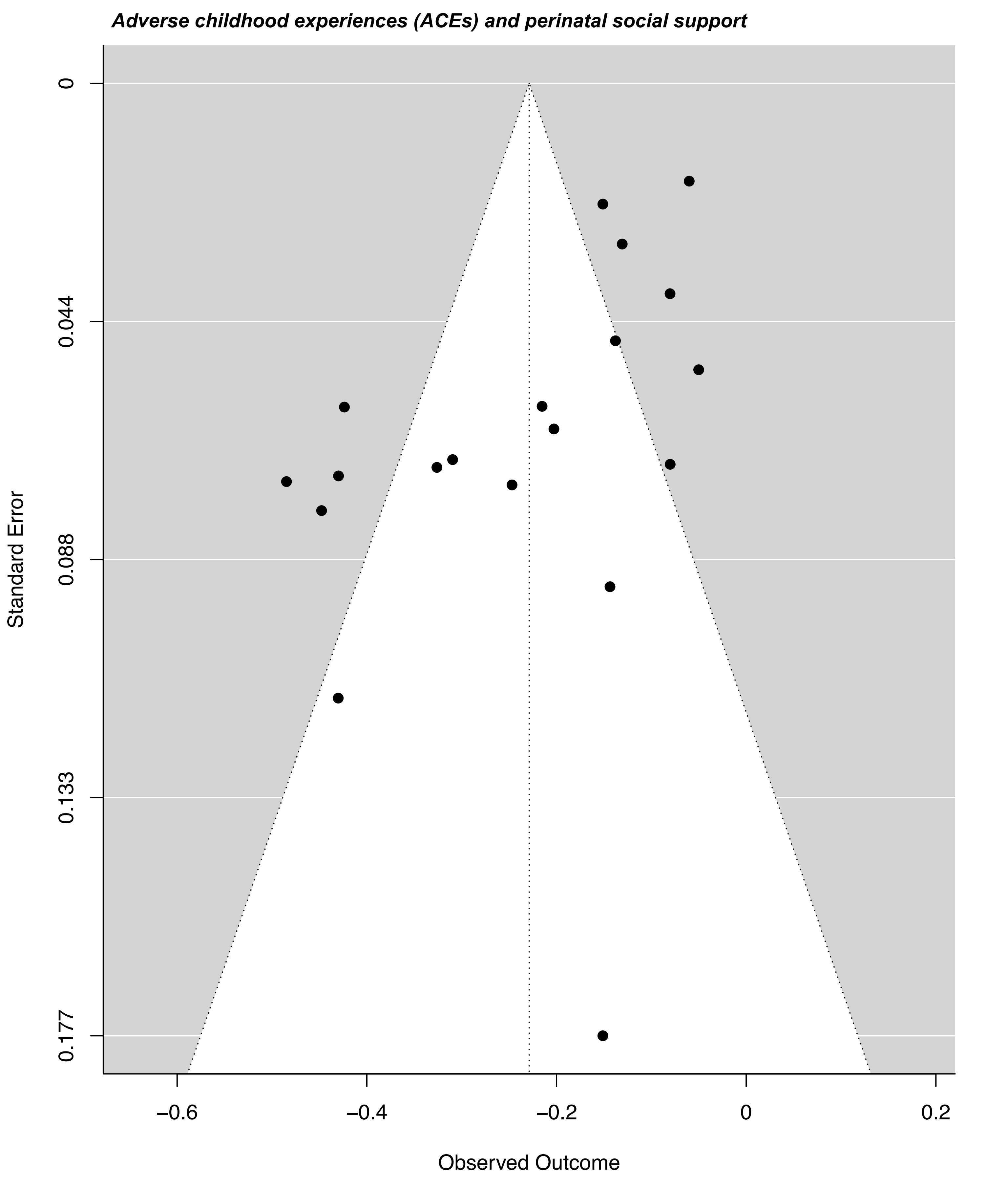

Adverse childhood experiences (ACEs) and perinatal social support
Standard Error
0
0.044
0.088
0.133
0.177
−0.6
−0.4
−0.2
0
0.2
Observed Outcome

Migration history and perinatal social support

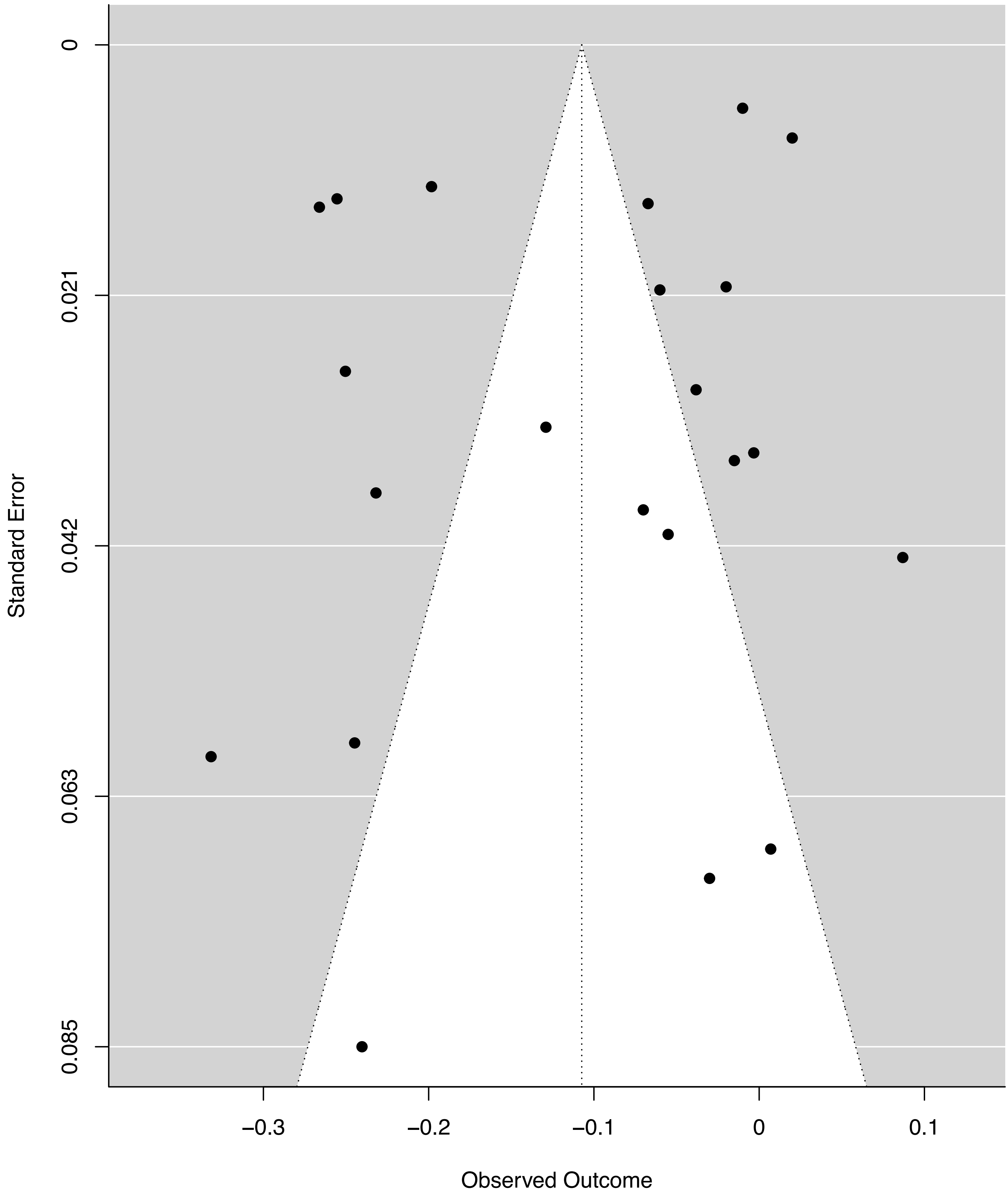

Reference list of included studies

Abadi, M. N. L., Ghazinour, M., Nojomi, M., & Richter, J. (2012). The buffering effect of social support between domestic violence and self-esteem in pregnant women in Tehran, Iran. *Journal of Family Violence, 27*(3), 225-231. doi:10.1007/s10896-012-9420-x

Agostini, F., Monti, F., Paterlini, M., Andrei, F., Palomba, S., & La Sala, G. B. (2018). Effect of the previous reproductive outcomes in subfertile women after in vitro fertilization (IVF) and/or intracytoplasmic sperm injection (ICSI) treatments on perinatal anxious and depressive symptomatology. *Journal of Psychosomatic Obstetrics & Gynecology, 39*(1), 29-37. doi:10.1080/0167482X.2017.1286474

Agostini, F., Neri, E., Salvatori, P., Dellabartola, S., Bozicevic, L., & Monti, F. (2015). Antenatal depressive symptoms associated with specific life events and sources of social support among Italian women. *Maternal and Child Health Journal, 19*(5), 1131-1141. doi:10.1007/s10995-014-1613-x

Ahmad, S. I., Rudd, K. L., LeWinn, K. Z., Mason, W. A., Murphy, L., Juarez, P. D., . . . Bush, N. R. (2022). Maternal childhood trauma and prenatal stressors are associated with child behavioral health. *Journal of developmental origins of health and disease, 13*(4), 483-493. doi:10.1017/S2040174421000581

Alfayumi-Zeadna, S., Froimovici, M., Rourke, N. O., Azbarga, Z., Okby-Cronin, R., Salman, L., . . . Daoud, N. (2021). Direct and indirect determinants of prenatal depression among Arab-Bedouin women in Israel: The role of stressful life events and social support. *Midwifery, 96*, 102937. doi:10.1016/j.midw.2021.102937

Almeida, J., Mulready-Ward, C., Bettegowda, V. R., & Ahluwalia, I. B. (2014). Racial/ethnic and nativity differences in birth outcomes among mothers in New York City: The role of social ties and social support. *Maternal and Child Health Journal, 18*(1), 90-100. doi:10.1007/s10995-013-1238-5

Almeida, L. M., Costa-Santos, C., Caldas, J. P., Dias, S., & Ayres-de-Campos, D. (2016). The impact of migration on women's mental health in the postpartum period. *Revista de Saude Publica, 50*. doi:10.1590/S1518-8787.2016050005617

Ammerman, R. T., Scheiber, F. A., Peugh, J. L., Messer, E. P., Van Ginkel, J. B., & Putnam, F. W. (2019). Interpersonal trauma and suicide attempts in low-income depressed mothers in home visiting. *Child Abuse and Neglect, 97*. doi:10.1016/j.chiabu.2019.104126

Ammerman, R. T., Shenk, C. E., Teeters, A. R., Noll, J. G., Putnam, F. W., & Van Ginkel, J. B. (2013). Multiple mediation of trauma and parenting stress in mothers in home visiting. *Infant Mental Health Journal, 34*(3), 234-241. doi:10.1002/imhj.21383

Appleton, A. A., Kiley, K., Holdsworth, E. A., & Schell, L. M. (2019). Social support during pregnancy modifies the association between maternal adverse childhood experiences and infant birth size. *Maternal & Child Health Journal, 23*(3), 408-415. doi:10.1007/s10995-018-02706-z

Asselmann, E., Wittchen, H. U., Erler, L., & Martini, J. (2016). Peripartum changes in social support among women with and without anxiety and depressive disorders prior to pregnancy: a prospective-longitudinal study. *Archives of Women's Mental Health, 19*(6), 943-952. doi:10.1007/s00737-016-0608-6

Bahrami, N., Allen, K. A., & Alimoardi, Z. (2023). Predictors of preconception care reception in a sample of Iranian pregnant women referred to Qazvin health centers. *Current Women's Health Reviews, 19*(3), 108-119. doi:10.2174/1573404819666220620091743

Bales, M., Pambrun, E., Maguet, C., van der Waerden, J., Glangeaud-Freudenthal, N., Charles, M.-A., . . . Sutter-Dallay, A.-L. (2023). Pathways between Risk/Protective Factors and Maternal Postnatal Depressive Symptoms: The ELFE Cohort. *Journal of Clinical Medicine, 12*(9). doi:10.3390/jcm12093204

Ballantyne, M., Benzies, K. M., & Trute, B. (2013). Depressive symptoms among immigrant and Canadian born mothers of preterm infants at neonatal intensive care discharge: a cross sectional study. *BMC Pregnancy & Childbirth, 13*(1), S11-S11. doi:10.1186/1471-2393-13-S1-S11

Bandyopadhyay, M., Small, R., Watson, L. F., & Brown, S. (2010). Life with a new baby: how do immigrant and Australian-born women's experiences compare? *Australian and New Zealand Journal of Public Health, 34*(4), 412-421. doi:10.1111/j.1753-6405.2010.00575.x

Barclay, M. E., Rinne, G. R., Somers, J. A., Lee, S. S., Coussons-Read, M., & Dunkel Schetter, C. (2022). Maternal early life adversity and infant stress regulation: Intergenerational associations and mediation by maternal prenatal mental health. *Research on child and adolescent psychopathology*, *51*, 1839-1855. doi:10.1007/s10802-022-01006-z

Barton, K., Redshaw, M., Quigley, M. A., & Carson, C. (2017). Unplanned pregnancy and subsequent psychological distress in partnered women: a cross-sectional study of the role of relationship quality and wider social support. *BMC Pregnancy & Childbirth, 17*, 1-9. doi:10.1186/s12884-017-1223-x

Baumgartel, K., Saint Fleur, A., Prescott, S., Fanfan, D., Elliott, A., Yoo, J. Y., . . . Groer, M. W. (2023). Social determinants of health among pregnant Hispanic women and associated psychological outcomes. *Journal of racial and ethnic health disparities*. doi:10.1007/s40615-023-01852-7

Bazzazian, S., Ozgoli, G., Riazi, H., Mahmoodi, Z., Vafa, M., & Nasiri, M. (2023). The relationship between social determinants of health and postpartum weight retention based on the World Health

Organization model: path analysis. *BMC public health, 23*(1), 323. doi:10.1186/s12889-023-15207-8

Bolak Boratav, H., Toker, Ö., & Küey, L. (2016). Postpartum depression and its psychosocial correlates: A longitudinal study among a group of women in Turkey. *Women & Health, 56*(5), 502-521. doi:10.1080/03630242.2015.1101737

Boothe, A. S., Brouwer, R. J. N., Carter-Edwards, L., & Østbye, T. (2011). Unmet social support for healthy behaviors among overweight and obese postpartum women: results from the active mothers postpartum study. *Journal of Women's Health, 20*(11), 1677-1685. doi:10.1089/jwh.2010.2509

Bouchard, G., & Doucet, D. (2011). Parental divorce and couples' adjustment during the transition to parenthood: The role of parent-adult child relationships. *Journal of Family Issues, 32*(4), 507-527. doi:10.1177/0192513X10389304

Boyce, W. T., Schaefer, C., Harrison, H. R., Haffner, W. H., Lewis, M., & Wright, A. L. (1986). Social and cultural factors in pregnancy complications among Navajo women. *American Journal of Epidemiology, 124*(2), 242-253.

Byrskog, U., Small, R., & Schytt, E. (2020). Community-based bilingual doulas for migrant women in labour and birth – findings from a Swedish register-based cohort study. *BMC Pregnancy and Childbirth, 20*(1). doi:10.1186/s12884-020-03412-x

Chaaya, M., Awwad, J., Campbell, O. M. R., Sibai, A., & Kaddour, A. (2003). Demographic and psychosocial profile of smoking among pregnant women in Lebanon: public health implications. *Maternal & Child Health Journal, 7*(3), 179-186.

Chan, K. L. (2019). The role of father involvement and intimate partner violence on postnatal depression among women with unintended pregnancy. *Journal of Interpersonal Violence*, *36*(17-18), 9864-9884. doi:10.1177/0886260519862274

Chou, F., Avant, K. C., Kuo, S., & Fetzer, S. J. (2008). Relationships between nausea and vomiting, perceived stress, social support, pregnancy planning, and psychosocial adaptation in a sample of mothers: A questionnaire survey. *International Journal of Nursing Studies, 45*(8), 1185-1191. https://doi.org/10.1016/j.ijnurstu.2007.08.004

Coller, K. M., Chao, S. M., Lu, M. C., & Strobino, D. (2014). Unintended births among adult immigrant and U.S.-born Mexican women in the Los Angeles Mommy and Baby (LAMB) survey. *Women's Health Issues, 24*(4), e365-e372. doi:10.1016/j.whi.2014.03.005

Dadi, A. F., Miller, E. R., Woodman, R., Bisetegn, T. A., & Mwanri, L. (2020). Antenatal depression and its potential causal mechanisms among pregnant mothers in Gondar town: application of structural equation model. *BMC Pregnancy & Childbirth, 20*(1), 168-168. doi:10.1186/s12884-020-02859-2

Darwiche, J., Milek, A., Antonietti, J.-P., & Vial, Y. (2019). Partner support during the prenatal testing period after assisted conception. *Women & Birth, 32*(2), e264-e271. doi:10.1016/j.wombi.2018.07.006

Dayton, C. J., Brown, S., Goletz, J., Hicks, L., Barron, C., Sperlich, M., & Smith-Darden, J. (2019). Pathways to parenting: predictors of prenatal bonding in a sample of expectant mothers and fathers exposed to contextual risk. *Journal of Child & Family Studies, 28*(4), 1134-1144. doi:10.1007/s10826-019-01343-6

de Oliveira, E. A., Barros, F. C., da Silva Anselmi, L. D., & Piccinini, C. A. (2006). The quality of home environment in Brazil: An ecological model. *Journal of Child and Family Studies, 15*(5), 633-644. doi:10.1007/s10826-006-9063-8

Dejin-Karlsson, E., & Östergren, P. O. (2004). Country of origin, social support and the risk of small for gestational age birth. *Scandinavian Journal of Public Health, 32*(6), 442-449. doi:10.1080/14034940410028172

Do Leal, M. C., Lamarca, G. A., Pereira, A. P. E., & Vettore, M. V. (2012). The relationship between social capital, social support and the adequate use of prenatal care. *International Journal of Gynecology and Obstetrics, 119*, S701. doi:10.1016/S0020-7292(12)61716-0

Dol, J., Richardson, B., Grant, A., Aston, M., McMillan, D., Tomblin Murphy, G., & Campbell-Yeo, M. (2021). Influence of parity and infant age on maternal self-efficacy, social support, postpartum anxiety, and postpartum depression in the first six months in the Maritime Provinces, Canada. *Birth: Issues in Perinatal Care, 48*(3), 438-447. doi:10.1111/birt.12553

Dotlic, J., Terzic, M., Babic, D., Vasiljevic, N., Janosevic, S., Janosevic, L., & Pekmezovic, T. (2014). The influence of body mass index on the perceived quality of life during pregnancy. *Applied Research in Quality of Life, 9*(2), 387-399. doi:10.1007/s11482-013-9224-z

Dragonas, T., Petrogiannis, K., & Adam, H. (1997). Working women, their emotional well-being and pregnancy in Greece. *Journal of Reproductive and Infant Psychology, 15*(3-4), 239-256. doi:10.1080/02646839708404547

Drummond, J., & Rickwood, D. (1997). Childbirth confidence: validating the childbirth self-efficacy inventory (CBSEI) in an Australian sample. *Journal of Advanced Nursing (Wiley-Blackwell), 26*(3), 613-622. doi:10.1046/j.1365-2648.1997.t01-24-00999.x

Durkin, S., Morse, C., & Buist, A. (2001). The factor structure of prenatal psychological and psychosocial functioning in first-time expectant parents. *Journal of Reproductive & Infant Psychology, 19*(2), 121-134.

Erbaba, H., & Pinar, G. (2021). Association of perceived social support and maternal adaptation with postpartum depression in mothers of infants hospitalized in neonatal intensive care units. *Journal of Neonatal Nursing, 27*(4), 251-256. doi:10.1016/j.jnn.2020.11.005

Erkal Aksoy, Y., Bay, H., & Dereli Yilmaz, S. (2022). Factors affecting primiparous women's breastfeeding self-fffficacy levels. *Clinical Lactation, 13*(2), 100-111. doi:10.1891/CL-2021-0016

Folger, A. T., Nidey, N., Ding, L., Ji, H., Yolton, K., Ammerman, R. T., & Bowers, K. A. (2022). Association between maternal adverse childhood experiences and neonatal SCG5 DNA methylation - effect modification by prenatal home visiting. *American Journal of Epidemiology, 191*(4), 636-645. doi:10.1093/aje/kwab270

Frank, R. (2005). International migration and infant health in Mexico. *Journal of Immigrant Health, 7*(1), 11-22.

Galbally, M., Watson, S. J., Boyce, P., & Lewis, A. J. (2019). The role of trauma and partner support in perinatal depression and parenting stress: An Australian pregnancy cohort study. *International Journal of Social Psychiatry, 65*(3), 225-234. doi:10.1177/0020764019838307

Gameiro, S., Boivin, J., Canavarro, M. C., Moura-Ramos, M., & Soares, I. (2010). Social nesting: Changes in social network and support across the transition to parenthood in couples that conceived spontaneously or through assisted reproductive technologies. *Journal of Family Psychology, 24*(2), 175-187. doi:10.1037/a0019101

Gameiro, S., Moura-Ramos, M., Canavarro, M. C., & Soares, I. (2011). Network support and parenting in mothers and fathers who conceived spontaneously or through assisted reproduction. *Journal of Reproductive & Infant Psychology, 29*(2), 170-182. doi:10.1080/02646838.2011.553950

Gameiro, S., Canavarro, M. C., Boivin, J., Moura-Ramos, M., Soares, I., & Almeida Santos, T. (2011b). Parental investment in couples who conceived spontaneously or with assisted reproductive techniques. *Human Reproduction, 26*(5), 1128-1137. doi:10.1093/humrep/der031

Gariepy, A. M., Lundsberg, L. S., Miller, D., Stanwood, N. L., & Yonkers, K. A. (2016). Are pregnancy planning and pregnancy timing associated with maternal psychiatric illness, psychological distress and support during pregnancy? *Journal of Affective Disorders, 205*, 87-94. doi:10.1016/j.jad.2016.06.058

González-de la Torre, H., Miñarro-Jiménez, S., Palma-Arjona, I., Jeppesen-Gutierrez, J., Berenguer-Pérez, M., & Verdú-Soriano, J. (2021). Satisfacción percibida por las mujeres durante el parto en el Hospital Universitario Materno-Infantil de Canarias a través del Childbirth Experience Questionnaire (CEQ-E). *Enfermeria clinica, 31*(1), 21-30. doi:10.1016/j.enfcli.2020.05.022

Gulick, E. E. (2003). Adaptation of the postpartum support questionnaire for mothers with multiple sclerosis. *Research in Nursing & Health, 26*(1), 30-39.

Gürber, S., Bielinski-Blattmann, D., Lemola, S., Jaussi, C., von Wyl, A., Surbek, D., . . . Stadlmayr, W. (2012). Maternal mental health in the first 3-week postpartum: The impact of caregiver support and the

subjective experience of childbirth—A longitudinal path model. *Journal of Psychosomatic Obstetrics & Gynecology, 33*(4), 176-184. doi:10.3109/0167482X.2012.730584

Harley, K., & Eskenazi, B. (2006). Time in the United States, social support and health behaviors during pregnancy among women of Mexican descent. *Social Science & Medicine, 62*(12), 3048-3061.

Hauck, Y. L., Fenwick, J., Dhaliwal, S. S., Butt, J., & Schmied, V. (2011). The association between women's perceptions of professional support and problems experienced on breastfeeding cessation: a Western Australian study. *Journal of human lactation: Official Journal of International Lactation Consultant Association, 27*(1), 49-57. doi:10.1177/0890334410386956

Hickey, G., McGilloway, S., Leckey, Y., Furlong, M., Leavy, S., Stokes, A., . . . Donnelly, M. (2019). Mothers' well-being, parenting attitudes, and home environment: Cumulative risk and parity in early motherhood. *Child: Care, Health & Development, 45*(4), 523-530. doi:10.1111/cch.12677

Hildingsson, I., & Sjöling, M. (2011). Fathers' experiences of support during pregnancy and the first year following childbirth – Findings from a Swedish regional survey. *Journal of Men's Health, 8*(4), 258-266. doi:10.1016/j.jomh.2011.03.007

Hildingsson, I., Tingvall, M., & Rubertsson, C. (2008). Partner support in the childbearing period -- a follow up study. *Women and Birth : Journal of the Australian College of Midwives, 21*(4), 141-148. doi:10.1016/j.wombi.2008.07.003

Hinic, K. (2016). Predictors of breastfeeding confidence in the early postpartum period. *JOGNN: Journal of Obstetric, Gynecologic & Neonatal Nursing, 45*(5), 649-660. doi:10.1016/j.jogn.2016.04.010

Hitzler, M., Behnke, A., Gündel, H., Ziegenhain, U., Kindler, H., Kolassa, I. T., & Zimmermann, J. (2022). Sources of social support for postpartum women with a history of childhood maltreatment: Consequences for perceived stress and general mental health in the first year after birth. *Child Abuse and Neglect, 134*. doi:10.1016/j.chiabu.2022.105911

Hung, C.-H., Yu, C.-Y., Chang, S.-J., & Stocker, J. (2011). Postpartum psychosocial changes among experienced and inexperienced mothers in Taiwan. *Journal of Transcultural Nursing, 22*(3), 217-224. doi:10.1177/1043659611404432

Huth-Bocks, A. C., Levendosky, A. A., Bogat, G. A., & Von Eye, A. (2004). The impact of maternal characteristics and contextual variables on infant–mother attachment. *Child Development, 75*(2), 480-496. https://doi.org/10.1111/j.1467-8624.2004.00688.x

İsbİr, G. G., İncİ, F., Bektaş, M., Yıldız, P. D., & Ayers, S. (2016). Risk factors associated with post-traumatic stress symptoms following childbirth in Turkey. *Midwifery, 41*, 96-103. doi:10.1016/j.midw.2016.07.016

Jarlenski, M., McManus, J., Diener-West, M., Schwarz, E. B., Yeung, E., & Bennett, W. L. (2014). Association between support from a health professional and breastfeeding knowledge and practices among

obese women: Evidence from the Infant Practices Study II. *Women's Health Issues, 24*(6), 641-648. doi:10.1016/j.whi.2014.08.002

Jewell, S. L., Letham-Hamlett, K., Hanna Ibrahim, M., Luecken, L. J., & MacKinnon, D. P. (2017). Family support and family negativity as mediators of the relation between acculturation and postpartum weight in low-income Mexican-origin women. *Annals of Behavioral Medicine, 51*(6), 856-867. doi:10.1007/s12160-017-9909-x

Kakyo, T. A., Muliira, J. K., Mbalinda, S. N., Kizza, I. B., & Muliira, R. S. (2012). Factors associated with depressive symptoms among postpartum mothers in a rural district in Uganda. *Midwifery, 28*(3), 374-379. doi:10.1016/j.midw.2011.05.001

Katzow, M. W., Messito, M. J., Mendelsohn, A. L., Scott, M. A., & Gross, R. S. (2023). Protective effect of prenatal social support on the intergenerational transmission of obesity in low-income Hispanic families. *Child Obes, 19*(6), 382-390. doi:10.1089/chi.2021.0306

Killoren, S., Zeiders, K., Updegraff, K., & Umaña-Taylor, A. (2016). The sociocultural context of Mexican-origin pregnant adolescents' attitudes toward teen pregnancy and links to future outcomes. *Journal of Youth & Adolescence, 45*(5), 887-899. doi:10.1007/s10964-015-0387-9

Kingston, D., Sword, W., Krueger, P., Hanna, S., & Markle-Reid, M. (2012). Life course pathways to prenatal maternal stress. *Journal of Obstetric, Gynecologic, & Neonatal Nursing: Clinical Scholarship for the Care of Women, Childbearing Families, & Newborns, 41*(5), 609-626. doi:10.1111/j.1552-6909.2012.01381.x

Kita, A. (2000). Quality of social network for pregnant women in Japan with focus on parity and family structure. *The Kobe journal of medical sciences, 46*(3), 125-136.

Kitamura, T., Toda, M. A., Shima, S., Sugawara, K., & Sugawara, M. (1998). Social support and pregnancy: I. Factorial structure and psychosocial correlates of perceived social support. *Psychiatry and Clinical Neurosciences, 52*(1), 29-36. doi:10.1111/j.1440-1819.1998.tb00969.x

Kozhimannil, K. B., Attanasio, L. B., Jou, J., Joarnt, L. K., Johnson, P. J., & Gjerdingen, D. K. (2014). Potential benefits of increased access to doula support during childbirth. *The American journal of managed care, 20*(8), e340-e352.

Kroelinger, C. D., & Oths, K. S. (2000). Partner support and pregnancy wantedness. *Birth: Issues in Perinatal Care, 27*(2), 112-119.

Leraand, G. G. (1999). *Social support and risk of depression at eight weeks postpartum in mothers in rural Alberta*. [Unpublished master dissertation]. University of Alberta.

Lesar, M. D. (2012). *Health and Behavior Among Latina Women and Their Children: Associations with Social Support, Perception of Safety, and Neighborhood Disorder.* [Doctoral Dissertation, University of California, Berkeley]. https://escholarship.org/uc/item/4pp810f3

Lewis, M. W. (2006). Relationship of prior custody loss to maternal-fetal bonding in a subsequent pregnancy. *Children and Youth Services Review, 28*(10), 1169-1180. doi:10.1016/j.childyouth.2005.11.011

Lewis, M. W., Lanzara, B. L., Stein, J. L., & Hasin, D. S. (2005). Maternal drinking patterns and drug use increase impact of terrorism among pregnant women attending prenatal care. *Journal of Prenatal & Perinatal Psychology & Health, 19*(4), 275-288.

Luecken, L. J., Lin, B., Coburn, S. S., MacKinnon, D. P., Gonzales, N. A., & Crnic, K. A. (2013). Prenatal stress, partner support, and infant cortisol reactivity in low-income Mexican American families. *Psychoneuroendocrinology, 38*(12), 3092-3101. doi:10.1016/j.psyneuen.2013.09.006

Luecken, L. J., Purdom, C. L., & Howe, R. (2009). Prenatal care initiation in low-income Hispanic women: Risk and protective factors. *American Journal of Health Behavior, 33*(3), 264-275. doi:10.5993/AJHB.33.3.5

Lydon, K., McGuire, B., Owens, L., Sarma, K., Avalos, G., O'Connor, C., . . . Dunne, F. (2011). Atlantic DIP: Diabetes and stress during pregnancy-A comparative psychological study. *Diabetes, 60*, A637. doi:10.2337/db11-2185-2431

Martínez Herreros, M. C., Rodríguez Muñoz, M. F., Izquierdo Méndez, N., & Olivares Crespo, M. E. (2022). Psychological, psychosocial and obstetric differences between Spanish and immigrant mothers: Retrospective observational study. *International Journal of Environmental Research and Public Health, 19*(18). doi:10.3390/ijerph191811782

McCormack, C., Lauriola, V., Spann, M., Berry, O., Lee, S., Mitchell, A., . . . Monk, C. (2020). Maternal childhood maltreatment, lifestyle factors, and immune activation during pregnancy. *Psychosomatic Medicine, 82*(6), A128. doi:10.1097/PSY.0000000000000835

McCormick, M. C., Brooks-Gunn, J., Shorter, T., Wallace, C. Y., Holmes, J. H., & Heagarty, M. C. (1987). The planning of pregnancy among low-income women in central Harlem. *American Journal of Obstetrics and Gynecology, 156*(1), 145-149.

McFarland, K. V., Hefelfinger, L. M., Mendez, C., DeFranco, E. A., & Kelly, E. (2022). Social determinants of health among Black people during pregnancies following a short interpregnancy interval. *American Journal of Obstetrics and Gynecology, 226*(1), S249. doi:10.1016/j.ajog.2021.11.421

McVeigh, C., & Smith, M. (2000). A comparison of adult and teenage mother's self-esteem and satisfaction with social support. *Midwifery, 16*(4), 269-276.

Milan, S., Lewis, J., Ethier, K., Kershaw, T., & Ickovics, J. R. (2004). The impact of physical maltreatment history on the adolescent mother-infant relationship: mediating and moderating effects during the transition to early parenthood. *Journal of Abnormal Child Psychology, 32*(3), 249-261. doi:10.1023/B:JACP.0000026139.01671.fd

Miranda, M. L., & Maxson, P. (2011). Pregnancy intention, demographic differences, and psychosocial health. *Journal of Women's Health, 20*(8), 1215-1223. doi:10.1089/jwh.2010.2379

Miszkurka, M., Goulet, L., Zunzunegui, M. V., Miszkurka, M., Goulet, L., & Zunzunegui, M. V. (2010). Contributions of immigration to depressive symptoms among pregnant women in Canada. *Canadian Journal of Public Health, 101*(5), 358-364.

Miszkurka, M., Goulet, L., Zunzunegui, M. V., Miszkurka, M., Goulet, L., & Zunzunegui, M. V. (2012). Antenatal depressive symptoms among Canadian-born and immigrant women in Quebec: differential exposure and vulnerability to contextual risk factors. *Social Psychiatry & Psychiatric Epidemiology, 47*(10), 1639-1648. doi:10.1007/s00127-011-0469-2

Mortazavi, F., & Agah, J. (2018). Childbirth fear and associated factors in a sample of pregnant Iranian women. *Oman Medical Journal, 33*(6), 497-505. doi:10.5001/omj.2018.91

Muzik, M., Brier, Z., Menke, R. A., Davis, M. T., & Sexton, M. B. (2016). Longitudinal suicidal ideation across 18-months postpartum in mothers with childhood maltreatment histories. *Journal of Affective Disorders, 204*, 138-145. doi:10.1016/j.jad.2016.06.037

Muzik, M., Umarji, R., Sexton, M. B., & Davis, M. T. (2017). Family social support modifies the relationships between childhood maltreatment severity, economic adversity and postpartum depressive symptoms. *Maternal and Child Health Journal, 21*(5), 1018-1025. doi:10.1007/s10995-016-2197-4

Nakamura, Y., Okada, T., Morikawa, M., Yamauchi, A., Sato, M., Ando, M., & Ozaki, N. (2020). Perinatal depression and anxiety of primipara is higher than that of multipara in Japanese women. *Scientific reports, 10*(1), 17060. doi:10.1038/s41598-020-74088-8

Naveed, S., Lashari, U. G., Waqas, A., Bhuiyan, M., & Meraj, H. (2018). Gender of children and social provisions as predictors of unplanned pregnancies in Pakistan: a cross-sectional survey. *BMC research notes, 11*(1), 587. doi:10.1186/s13104-018-3696-8

Nelson, D. B., & Lepore, S. J. (2013). The role of stress, depression, and violence on unintended pregnancy among young urban women. *Journal of Women's Health (15409996), 22*(8), 673-680. doi:10.1089/jwh.2012.4133

Nowak, A. L., Braungart-Rieker, J. M., & Kuo, P. X. (2021). Social support moderates the relation between childhood trauma and prenatal depressive symptoms in adolescent mothers. *Journal of Reproductive and Infant Psychology*. doi:10.1080/02646838.2021.1938980

Noy, A., Taubman – Ben-Ari, O., Morag, I., & Kuint, J. (2020). Intergenerational relations, circumstances, and changes in mothers' marital quality during two years following childbirth. *Health Care for Women International, 41*(1), 101-120. doi:10.1080/07399332.2019.1590358

Odinka, P., Odinka, J., Ezeme, M., Ndukuba, A., Amadi, K., Muomah, R., . . . Nduanya, U. (2019). Socio-demographic correlates of postpartum psychological distress among apparently healthy mothers in two tertiary hospitals in Enugu, South-East Nigeria. *African Health Sciences, 19*(3), 2515-2525. doi:10.4314/ahs.v19i3.27

Ogawa, M., Hashimoto, T., Tanaka, M., Tachibana, M., Seki, R., Sato, A., . . . Iyo, M. (2019). The effect of grandmothers' presence on the provision of multidisciplinary perinatal support for pregnant and postpartum women with psychosocial problems. *Journal of Multidisciplinary Healthcare, 12*, 1033-1041. doi:10.2147/JMDH.S228320

Orne-Gliemann, J., Font, H., Maphosa, T., Kangwende, A., Rusakaniko, S., Magezi, V., . . . Foster, G. (2017). Patterns of attendance at mother support groups in Zimbabwe. the EPAZ trial (2014-2016). *Journal of Acquired Immune Deficiency Syndromes, 75*, S216-S223. doi:10.1097/QAI.0000000000001348

Orr, S. T., & Miller, C. A. (1997). Unintended pregnancy and the psychosocial well-being of pregnant women. *Women's Health Issues, 7*(1), 38-46.

Parker, G., & Barnett, B. (1988). Perceptions of parenting in childhood and social support in adulthood. *The American Journal of Psychiatry, 145*(4), 479-482. doi:10.1176/ajp.145.4.479

Pascoe, J. M., Kokotailo, P. K., & Broekhuizen, F. F. (1995). Correlates of multigravida women's binge drinking during pregnancy: A longitudinal study. *Archives of Pediatrics and Adolescent Medicine, 149*(12), 1325-1329.

Phillips, J., Sharpe, L., Matthey, S., Charles, M., Phillips, J., Sharpe, L., . . . Charles, M. (2010). Subtypes of postnatal depression? A comparison of women with recurrent and de novo postnatal depression. *Journal of Affective Disorders, 120*(1-3), 67-75. doi:10.1016/j.jad.2009.04.011

Powers, J. R., McDermott, L. J., Loxton, D. J., & Chojenta, C. L. (2013). A prospective study of prevalence and predictors of concurrent alcohol and tobacco use during pregnancy. *Maternal and Child Health Journal, 17*(1), 76-84. doi:10.1007/s10995-012-0949-3

Quinlivan, J., Petersen, I., Gibson, R., McPhee, A., & Makrides, M. (2014). Antenatal support and depressive symptomatology at six weeks and six months postpartum. *Journal of Paediatrics and Child Health, 50*, 103.

Racine, N., Madigan, S., Plamondon, A., Hetherington, E., McDonald, S., & Tough, S. (2018). Maternal adverse childhood experiences and antepartum risks: the moderating role of social support. *Archives of Women's Mental Health, 21*(6), 663-670. doi:10.1007/s00737-018-0826-1

Raghuveer, P., & Haleema, M. (2023). Perceived social support among pregnant women attending the antenatal clinic of a tertiary care hospital. *Journal of Education and Health Promotion, 12*, 88. doi:10.4103/jehp.jehp_1176_22

Records, K., Keller, C., Coonrod, D., Ainsworth, B., Todd, M., Belyea, M., . . . Lopez, S. V. (2015). Correlates of depressive symptoms after birth for Latinas who are overweight or obese. *Health Care for Women International, 36*(3), 356-374. doi:10.1080/07399332.2014.973956

Reisz, S., Brennan, J., Jacobvitz, D., & George, C. (2019). Adult attachment and birth experience: importance of a secure base and safe haven during childbirth. *Journal of Reproductive & Infant Psychology, 37*(1), 26-43. doi:10.1080/02646838.2018.1509303

Ren, Z., Zhang, A., Fan, X., Feng, J., & Xia, H. (2023). Utility of the capability, opportunity, and motivation behaviour (COM-B) model in explaining the negative association between pre-pregnancy body mass index and exclusive breastfeeding at six weeks postpartum. *Appetite, 188*, 106631. doi:10.1016/j.appet.2023.106631

Salmela-Aro, K., Nurmi, J.-E., Saisto, T., & Halmesmäki, E. (2010). Spousal support for personal goals and relationship satisfaction among women during the transition to parenthood. *International Journal of Behavioral Development, 34*(3), 229-237. doi:10.1177/0165025409350956

Salonen, A. H., Oommen, H., & Kaunonen, M. (2014). Primiparous and multiparous mothers' perceptions of social support from nursing professionals in postnatal wards. *Midwifery, 30*(4), 476-485. doi:10.1016/j.midw.2013.05.010

Samdan, G., Reinelt, T., Kiel, N., Mathes, B., & Pauen, S. (2022). Maternal self-efficacy development from pregnancy to 3 months after birth. *Infant Mental Health Journal*. doi:10.1002/imhj.22018

Saravanan, V., Desai, G., & Satyanarayana, V. A. (2023). Antenatal predictors of postnatal maternal attachment and competence after assisted conception-a prospective cohort study in South India. *Archives of Women's Mental Health, 26*(4), 549-560. doi:10.1007/s00737-023-01340-1

Sayil, M., Güre, A., & Uçanok, Z. (2006). First time mothers' anxiety and depressive symptoms across the transition to motherhood: associations with maternal and environmental characteristics. *Women & Health, 44*(3), 61-77.

Schury, K., Zimmermann, J., Umlauft, M., Hulbert, A. L., Guendel, H., Ziegenhain, U., & Kolassa, I. T. (2017). Childhood maltreatment, postnatal distress and the protective role of social support. *Child Abuse and Neglect, 67*, 228-239. doi:10.1016/j.chiabu.2017.02.021

Shwartz, N., O'Rourke, N., & Daoud, N. (2020). Pathways linking intimate partner violence and postpartum depression among Jewish and Arab women in Israel. *Journal of Interpersonal Violence*, *37*(1-2), 301-321. doi:10.1177/0886260520908022

Sockol, L. E., & Battle, C. L. (2015). Maternal attitudes, depression, and anxiety in pregnant and postpartum multiparous women. *Archives of Women's Mental Health, 18*(4), 585-593. doi:10.1007/s00737-015-0511-6

Soriano-Vidal, F. J., Oliver-Roig, A., Cabrero-García, J., Congost-Maestre, N., Dencker, A., & Richart-Martínez, M. (2016). The Spanish version of the Childbirth Experience Questionnaire (CEQ-E): reliability and validity assessment. *BMC Pregnancy & Childbirth, 16*, 372-372. doi:10.1186/s12884-016-1100-z

Spry, E. A., Moreno-Betancur, M., Middleton, M., Howard, L. M., Brown, S. J., Molyneaux, E., . . . Patton, G. C. (2021). Preventing postnatal depression: a causal mediation analysis of a 20-year preconception cohort. *Philosophical transactions of the Royal Society of London. Series B, Biological sciences, 376*: 20200028. doi:10.1098/rstb.2020.0028

Spry, E. A., Olsson, C. A., Aarsman, S. R., Mohamad Husin, H., Macdonald, J. A., Dashti, S. G., . . . Patton, G. C. (2023). Parental personality and early life ecology: a prospective cohort study from preconception to postpartum. *Sci Rep, 13*(1), 3332. doi:10.1038/s41598-023-29139-1

Stöckl, H., Hertlein, L., Himsl, I., Delius, M., Hasbargen, U., Friese, K., . . . Stöckl, D. (2012). Intimate partner violence and its association with pregnancy loss and pregnancy planning. *Acta Obstetricia et Gynecologica Scandinavica, 91*(1), 128-133. doi:10.1111/j.1600-0412.2011.01264.x

Surkan, P. J., Hong, X., Zhang, B., Nawa, N., Ji, H., Tang, W.-Y., . . . Wang, X. (2019). Can social support during pregnancy affect maternal DNA methylation? Findings from a cohort of African-Americans. *Pediatric Research*. doi:10.1038/s41390-019-0512-7

Szabó, N., Dubas, J. S., Volling, B. L., & van Aken, M. A. G. (2017). The effect of paternal and alloparental support on the interbirth interval among contemporary North American families. *Evolutionary Behavioral Sciences, 11*(3), 272-280. doi:10.1037/ebs0000093

Tambag, H., Turan, Z., Tolun, S., & Can, R. (2018). Perceived social support and depression levels of women in the postpartum period in Hatay, Turkey. *Nigerian journal of clinical practice, 21*(11), 1525-1530. doi:10.4103/njcp.njcp_285_17

Teyhan, A., Wijedasa, D., & MacLeod, J. (2018). Adult psychosocial outcomes of men and women who were looked-after or adopted as children: Prospective observational study. *BMJ Open, 8*(2). doi:10.1136/bmjopen-2017-019095

Thananowan, N. (2008). Intimate partner violence (IPV), risk factors, and health outcomes in adult pregnant women. *Thai Journal of Nursing Research, 12*(4), 310-323.

Thomas, J. C., Letourneau, N., Campbell, T. S., & Giesbrecht, G. F. (2018). Social buffering of the maternal and infant HPA axes: Mediation and moderation in the intergenerational transmission of adverse childhood experiences. *Development and Psychopathology, 30*(3), 921-939. doi:10.1017/S0954579418000512

Urquia, M. L., O'Campo, P. J., & Heaman, M. I. (2012). Revisiting the immigrant paradox in reproductive health: The roles of duration of residence and ethnicity. *Social Science and Medicine, 74*(10), 1610-1621. doi:10.1016/j.socscimed.2012.02.013

Versteegen, M., Bozlak, C., Larkin, H., & Appleton, A. (2021). Risk factors for gestational diabetes in upstate New York women & implications for practice. *Paediatric and Perinatal Epidemiology, 35*(SUPPL 1), 60-61. doi:10.1111/ppe.6-12779

Wakeel, F., Witt, W. P., Wisk, L. E., Lu, M. C., & Chao, S. M. (2014). Racial and ethnic disparities in personal capital during pregnancy: Findings from the 2007 Los Angeles Mommy and Baby (LAMB) study. *Maternal and Child Health Journal, 18*(1), 209-222. doi:10.1007/s10995-013-1256-3

Waraphorn, S., Darawan, T., Sirirat, P., & Piyanut, X. (2021). Factors Explaining Postpartum Depression among Thai Adolescent Mothers. *Pacific Rim International Journal of Nursing Research, 25*(1), 48-59.

Way, N., & Leadbeater, B. J. (1999). Pathways toward educational achievement among African American and Puerto Rican adolescent mothers: Re-examining the role of social support from families. *Development and Psychopathology, 11*(2), 349-364. doi:10.1017/S0954579499002096

Wells, M. B., Gedaly, L. R., & Aronson, O. (2023). Midwives and child health nurses' support is associated with positive coparenting for fathers of infants: A cross-sectional analysis. *Journal of clinical nursing, 32*(7-8), 1443-1454. doi:10.1111/jocn.16329

Weng, S. S., Lin, C. L., Tai, C. J., & Chien, L. Y. (2017). Differences between native and immigrant women in Taiwan in factors associated with caesarean section: A prospective observational study. *Women & Health, 57*(10), 1178-1192. doi:10.1080/03630242.2016.1243606

White-Traut, R., Rankin, K., Fabiyi, C., Liu, L., Cheung, I., & Norr, K. (2017). Maternal characteristics associated with social support in at-risk mothers of premature infants. *JOGNN: Journal of Obstetric, Gynecologic & Neonatal Nursing, 46*(6), 824-833. doi:10.1016/j.jogn.2017.07.008

Yanikkerem, E., Ay, S., & Piro, N. (2013). Planned and unplanned pregnancy: Effects on health practice and depression during pregnancy. *Journal of Obstetrics and Gynaecology Research, 39*(1), 180-187. doi:10.1111/j.1447-0756.2012.01958.x

Ying, T., Creedy, D. K., & Gamble, J. (2016). A comparison of life stress and depressive symptoms in pregnant Taiwanese and immigrant women. *Journal of Nursing Research, 24*(3), 272-280. doi:10.1097/jnr.0000000000000137

Young, R., Lane, W. G., Stephens, S. B., Mayden, B. W., & Fox, R. E. (2018). Psychosocial factors associated with healthy and unhealthy interpregnancy intervals. *Health equity, 2*(1), 22-29. doi:10.1089/heq.2017.0017

Younger, J. B. (1991). A model of parenting stress. *Research in Nursing & Health, 14*(3), 197-204.

Zain, N. M., Low, W. Y., & Othman, S. (2015). Factors associated with pregnancy among unmarried women in Malaysia. *The Southeast Asian Journal of Tropical Medicine and Public Health, 46*(3), 526-538.

Zelkowitz, P., Bardin, C., & Papageorgiou, A. (2007). Anxiety affects the relationship between parents and their very low birth weight infants. *Infant Mental Health Journal, 28*(3), 296-313. doi:10.1002/imhj.20137

Zhong, Q.-Y., Gelaye, B., VanderWeele, T. J., Sanchez, S. E., & Williams, M. A. (2018). Causal model of the association of social support with antepartum depression: A marginal structural modeling approach. *American Journal of Epidemiology, 187*(9), 1871-1879. doi:10.1093/aje/kwy067

Zlotnick, C., Manor-Lavon, I., & Leahy-Warren, P. (2023). Relationship between social support and postpartum depression in migrant and non-migrant first-time mothers. *Journal of Clinical Nursing, 32*(7/8), 1316-1326. doi:10.1111/jocn.16297

Additional references

Afifi, T. O. (2020). Considerations for expanding the definition of ACEs. In *Adverse Childhood Experiences: Using Evidence to Advance Research, Practice, Policy, and Prevention* (pp. 35–44). Elsevier. https://doi.org/10.1016/B978-0-12-816065-7.00003-3

Kubany, E. S., Haynes, S. N., Leisen, M. B., Owens, J. A., Kaplan, A. S., Watson, S. B., Burns, K., Blake, D., Fairbank, J., Foa, E., Green, B., Norris, F., Scurfield, R., Weathers, F., & Kubany, S. (2000). Development and Preliminary Validation of a Brief Broad-Spectrum Measure of Trauma Exposure: The Traumatic Life Events Questionnaire National Center for PTSD and HOPE Domestic Violence Consultants. *Psychological Assessment*, *12*(1), 210–224. https://doi.org/10.1037/1040-3590.I2.2.210

National Heart Lung and Blood Institute: National Institutes of Health. (2014). Quality Assessment Tool for Observational Cohort and Cross-Sectional Studies. https://www.nhlbi.nih.gov/health-topics/study-quality-assessment-tools

The World Bank. (2022). *The world by income and region.* https://datatopics.worldbank.org/world-development-indicators/the-world-by-income-and-region.html
